# The Efficacy of Lapatinib in Metastatic Breast Cancer with HER2 Non-Amplified Primary Tumors and EGFR Positive Circulating Tumor Cells: A Proof-Of-Concept Study

**DOI:** 10.1371/journal.pone.0062543

**Published:** 2013-05-07

**Authors:** Justin Stebbing, Rachel Payne, Justine Reise, Adam E. Frampton, Miranda Avery, Laura Woodley, Angelo Di Leo, Marta Pestrin, Jonathan Krell, R. Charles Coombes

**Affiliations:** 1 Department of Oncology, Imperial College Healthcare National Health Service Trust, Charing Cross Hospital, London, United Kingdom; 2 Sandro Pitigliani Medical Oncology Unit, Istituto Tosacano Tumori, Prato, Italy; National University of Ireland Galway, Ireland

## Abstract

**Background:**

Analysis of circulating tumor cells (CTCs) provides real-time measures of cancer sub-populations with potential for CTC-directed therapeutics. We examined whether lapatinib which binds both HER2 and EGFR could induce depletion of the EGFR-positive pool of CTCs, which may in turn lead to clinical benefits.

**Patients and Methods:**

Patients with metastatic breast cancer and HER2 non-amplified primary tumors with EGFR-positive CTCs were recruited and lapatinib 1500 mg daily was administered, in a standard two step phase 2 trial.

**Results:**

There were no responses leading to termination at the first analysis with 16 patients recruited out of 43 screened. In 6 out of 14 (43%) individuals eligible for the efficacy analysis, a decrease in CTCs was observed with most of these having a greater decrease in their EGFR-positive CTC pool.

**Conclusions:**

This is one of the first studies of CTC-directed therapeutics and suggests that lapatinib monotherapy is not having any demonstrable clinical effects by reducing the EGFR-positive pool of CTCs in HER2 non-amplified primary tumors. Our attempt to expand the pool of patients eligible for a targeted therapy was unsuccessful; the role of clonal populations in cancer biology and therapeutic strategies to control them will require extensive evaluation in years to come.

**Trial Registration:**

Clinical trials.gov NCT00820924

## Introduction

Despite recent progress in gene-expression profiling studies, the underlying biology of the various patterns of metastasis observed in different tumor types remains unclear. The detection and characterization of CTCs in cancer patients has provided important new information about the progression of metastatic events, information that has relevant implications for cancer prognosis and therapy.

Currently the use of targeted therapies, such as anti-HER2 directed treatment, is based on the view that metastatic cells are linear descendants of primary tumor cells and have conserved biologic features. However, a hallmark of most cancers is their genetic instability [1]. It appears that, despite the advent of targeted therapies, we pay insufficient regard to the expression of targets, the clonal selection process including the clonal expansion of cells which do not necessarily express the target. Indeed, CTCs may show different properties from primary tumor cells and biological characterization of CTCs may lead to the identification of appropriate treatments for advanced breast cancer patients [2], [3]. In one study for example, 9 out of 24 (38%) advanced breast cancer patients whose primary tumor was HER-2 FISH negative acquired HER-2 gene amplification in their CTCs. Of note, 4 of the 9 patients were treated with trastuzumab-based therapies and 3 of these treated patients had a clinical response despite being heavily pre-treated for advanced disease [4].

As there is no known ligand for HER2, some have suggested that the primary role of HER2 is to modulate signals after ligand binding to other HER-family receptors and ErbB2 containing heterodimers exert potent proliferative effects [5], [6]. Lapatinib acts as a dual inhibitor of both EGFR and ErbB2 tyrosine kinase activity. A number of clinical studies have however demonstrated that lapatinib lacks efficacy in individuals without HER2-amplified primary tumors although in the largest randomized study here effects were dependent on hormone receptor status, thought in turn to be a surrogate for EGFR and/or HER2 dependency [7], [8].

CTCs are well known to be predictive of progression-free and overall survival [9], [10]; they may also be a more reliable indicator of progression than traditional imaging methods [11]. An important finding from studies performed on CTCs is they provide a potential early opportunity to predict a response to systemic therapies within weeks from treatment initiation, potentially leading to changes in decision-making for patients with metastatic disease [12]–[14]. We have previously demonstrated that EGFR measurements on CTCs are reproducible and reliable over time [15]. The role of lapatinib in those patients in whom there is evidence of EGFR expression in the absence of HER-2 amplification is poorly understood. Although there have been reports of EGFR positive and HER-2-negative patients responding to gefitinib or lapatinib [16]–[20], these are infrequent, appear restricted to the small subset of tumors that co-express ER and EGFR, and produce conflicting results.

We therefore performed an open-label single-arm two step phase 2 study to investigate the potential clinical activity and safety of lapatinib in advanced breast cancer patients with HER2 non-amplified primary tumors with EGFR positive CTCs, thereby attempting to expand treatment options in patients who would not normally be considered for these therapies.

## Materials and Methods

### Ethics Statement

The protocol for this trial and supporting CONSORT checklist are available as supporting information; see Checklist S1 and Protocol S1. This study was approved by our institutional review board and obtained clinical trial approval status from our national governing body (National Research Ethics Service, London- Surrey Borders Research Ethics Committee) Clinical trials.gov identifier: NCT00820924. Informed written consent was obtained for all patients and all clinical investigation was conducted according to the principles expressed in the Declaration of Helsinki.

This study was performed alongside an Italian Study Group trial [21] examining the effects of lapatinib in advanced breast cancer patients with HER2 non-amplified primary tumors and HER2 positive CTCs. The UK Study Group examined effects in patients with HER2 non-amplified primary tumors and EGFR positive CTCs, with a primary endpoint of the overall response rate (ORR) according to RECIST criteria [22], [23]. Secondary endpoints included clinical benefit rate (SD or a PR or CR), time to tumor progression, safety (according to according to CTCAE v. 3.0), and the biologic effects of lapatinib on EGFR positive CTCs, and whether changes in their numbers correlated with clinical outcomes.

Patients with pre-treated metastatic breast cancer and with HER2 negative primary tumors (IHC 0 or 1+, or IHC2+ and no amplification on FISH as per published guidelines [24]), progressing on systemic therapy with radiologically assessable disease were screened for evidence of EGFR positive CTCs using our previously published methods [15]. Individuals with brain metastases were excluded and life expectancy >12 weeks and a normal cardiac ejection fraction was required; standard trial hematologic, hepatic and renal parameters applied. Concurrent bisphosphonates were allowed, and those with previous treatments with anti-HER2 or anti-EGFR therapies were excluded. Those with at least one EGFR positive CTC were invited to participate in the study in which patients received 1500 mg daily oral lapatinib daily for 4 weeks. Clinical assessments were performed every 4 weeks and the planned scanning schedule was 12-weekly, including measurement of ejection fraction (echocardiography). Recruitment commenced in June 2009 and ended in February 2012.

The purpose of this phase II trial was to reject lapatinib from further studies in metastatic breast cancer patients with a HER2 negative primary tumor and EGFR positive CTCs if insufficient activity was detected, and conversely, to accept lapatinib for further studies in this cohort if activity was evident. ‘Activity’ was defined as tumor response according to RECIST criteria. The clinical trial followed a two-stage design with an inactivity cut-off chosen equal to 5%, an activity cut-off chosen equal to 20%, a type I error (i.e. the probability of accepting an insufficiently active treatment) of 10%, and a type II error (i.e. the probability of rejecting an active treatment) of 5%. The first stage was designed to recruit 16 patients. In the case of no objective responses, the trial would be stopped for futility. In the case of at least 4 responses, the trial would be stopped for efficacy. If 1 to 3 objective responses were observed, the trial would progress to the second stage, in which 15 more patients would be enrolled. Of the 31 patients in total, objective responses in 3 patients would be considered inconclusive, while objective responses in less than 3 patients would be described as a negative trial.

Subjects were treated until disease progression or withdrawal from study due to unacceptable toxicity or consent withdrawal. The original LAP105594 protocol did not include serial CTC measurement but these were investigated to provide additional data. While CTC measurement is now licensed as part of routine clinical practice in metastatic breast cancer, additional loco-regional ethics committee approval and consents were obtained.

CTCs and their EGFR levels were measured as previously described in our methods paper indicating that measurement of the EGFR on CTCs was reliable and reproducible over time [15]; analysis of CTCs was undertaken during most patient’s follow up visits. Briefly, a 7.5 mL blood sample was taken in a CellSave preservative tube, kept at room temperature and processed within 72 hours. The system enriched for EpCAM (epithelial cell adhesion molecule) positive epithelial cells by incubating the sample with ferrofluid conjugated to anti-EpCAM antibodies. Cells were stained with the following fluorescent labelled monoclonal proprietary antibodies: EGFR-FITC or HER2-FITC (CellSearch HER2 Tumor Phenotyping Reagent), CD45-APC to distinguish the CTCs from leukocytes and pan-cytokeratin 8, 18 and 19 (CK-PE) to stain epithelial cells. Nucleic acids were stained using 4,6-diamidino-2-phenylindole. Samples were then scanned on the CellTracks analyzer II fluorescent microscope for analysis.

## Results

**Figure 1:**
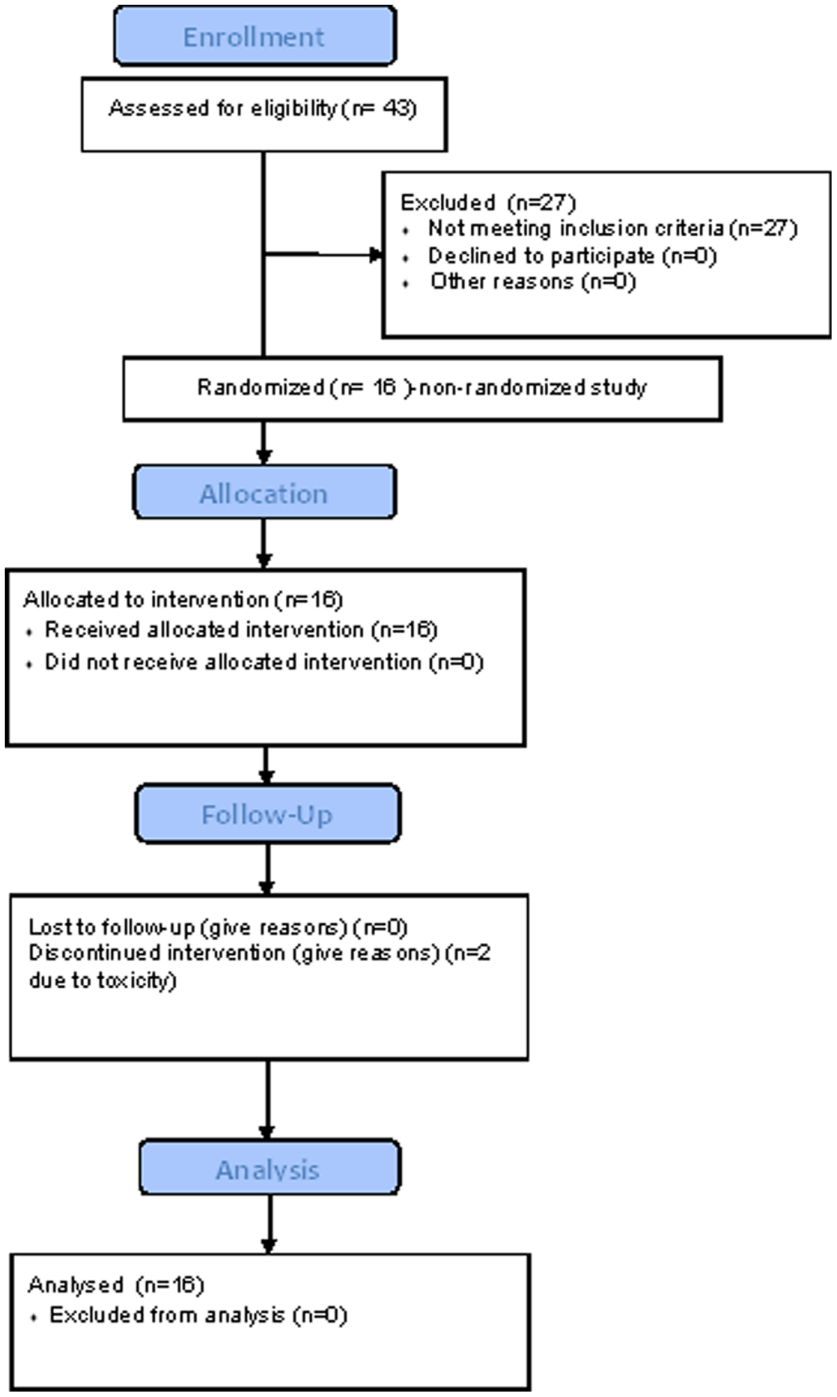
CONSORT 2010 flow diagram for patients enrolled in this study.

A total of 43 individuals were screened for EGFR positive CTCs (Figure 1). Of these, 16 patients (37%) were recruited to the study; 23 of the remaining 27 (85%) individuals had no EGFR positive CTCs. The remaining 4 patients had liver function tests that became too elevated in between screening and commencing drug. Of the 16 individuals who were recruited, no responses were observed and all patients progressed on study. Two individuals withdrew within 2 weeks after commencing lapatinib due to toxicity (both grade 3 diarrhea) and thus the per protocol primary efficacy analysis includes 14 patients, all of whom progressed within 12 weeks of entering the study (these 2 individuals also had rapidly progressive disease). There were no instances of stable disease, and thus the clinical benefit rate was zero. No patients died during the study although one developed brain metastases and died shortly afterwards.

Patient characteristics including baseline CTC levels for all 16 patients initially commenced on lapatinib are shown in Table 1. In 12 patients an extra blood tube was taken for measurement of HER2 levels on CTCs, as this was considered to be a potential confounding factor in the study (ie. patients could have responded by virtue of their HER2 positivity and we note that measurement of HER2 positive CTCs was not repeated following the initial test). Patients were extensively pre-treated with a median of 3 prior chemotherapeutic regimens and in those who had estrogen receptor positive (14 out of 16 patients) tumors, a median of 3 prior lines of hormonal therapy. Of the 16 patients included in the toxicity analysis, a total of 8 individuals received one cycle of lapatinib, 4 patients received 2 cycles and 4 individuals went on to receive 3 cycles prior to stopping due to disease progression. There were no cases of grade 3 or 4 hematologic toxicities; there were 2 cases of grade 3 AST/ALT elevations, 6 cases of grade 3 hyperbilirubinemia and 4 cases of grade 4 hyperbilirubinemia, all considered related to hepatic metastatic progression. Grade 3 diarrhea affected 2 individuals who immediately discontinued the study. The remaining side effects, notably rash and diarrhea were all considered grade I or II and manageable. There were no clinically significant decreases in ejection fraction.

**Table 1:** Primary tumor characteristics, prior treatments and CTC screening measurements of patients with advanced breast cancer.

Patient Number	Age	ECOG^a^status	Primary tumor characteristics (All HER2 negative)				Previous therapies (number of lines)			CTCs^c^ (% EGFR positive)	CTCs^c^ (% HER2 positive)
			TYPE^b^	ER	PgR	EGFR	CHEMO	HORMONAL	RADIO		
1	47	1	IDC	POS	N/K	N/K	X3	X4	X1	3/5 (60%)	–
2	52	1	ILC	POS	N/K	NEG	X4	X3	X2	2/8 (25%)	5/5 (100%)
3	63	2	IDC	POS	POS	N/K	X5	X5	X2	16/49 (33%)	6/8 (75%)
4	72	2	IDC	POS	NEG	N/K	X2	X2	–	2/13 (15%)	32/62 (52%)
5	68	1	ILC	POS	POS	NEG	X2	X3	–	9/34 (26%)	28/33 (85%)
6	67	1	IDC	POS	NEG	N/K	X3	X3	X1	37/221 (17%)	334/513 (65%)
7	45	1	IDC	POS	POS	NEG	X2	X3	–	1/12 (8%)	15/18 (83%)
8	39	2	IDC	NEG	NEG	N/K	X3		X5	44/66 (67%)	26/41 (63%)
9	61	1	IDC	POS	POS	N/K	X3	X4	X3	1/6 (12%)	14/26 (54%)
10	52	0	IDC	POS	POS	N/K	X2	X3	X1	3/14 (21%)	–
11	48	0	IDC	POS	POS	N/K	X3	X5	X2	1/13 (8%)	7/12 (58%)
12	51	0	IDC	POS	NEG	N/K	X1	X2	X3	5/62 (8%)	30/76 (39%)
13	64	0	PAP	NEG	NEG	N/K	X4	X1	X2	1/5 (20%)	–
14	64	1	IDC	POS	POS	NEG	X2	X2	X1	2/41 (5%)	–
15	50	0	IDC	POS	POS	NEG	X6	X3	X2	2/7 (29%)	0/4 (0%)
16	63	1	IDC	POS	POS	N/K	X1	X4	X1	1/2 (50%)	0/0 (0%)

Sixteen patients were recruited to the trial of a daily dose of lapatinib monotherapy. All patients had one or more EGFR positive CTCs. Twelve patients were assessed for HER2 positivity in their CTCs, and of the eleven assessable, ten (91%) had a proportion that were HER2 positive.

a Eastern Cooperative Oncology Group Status. 0 - Fully active, able to carry on all pre-disease performance without restriction, 1-Restricted in physically strenuous activity but ambulatory and able to carry out work of a light or sedentary nature, e.g., light house work, office work, 2 - Ambulatory and capable of all self-care but unable to carry out any work activities. Up and about more than 50% of waking hours.

b IDC – Invasive ductal carcinoma, ILC – Invasive lobular carcinoma, PAP - Papillary.

c CTCs present in 7.5 ml blood at screening or baseline as assessed by the CellSearch system.

NK = not known, the assessment of EGFR on the primary tumor being made according to our published methods [25].

**Figure 2:**
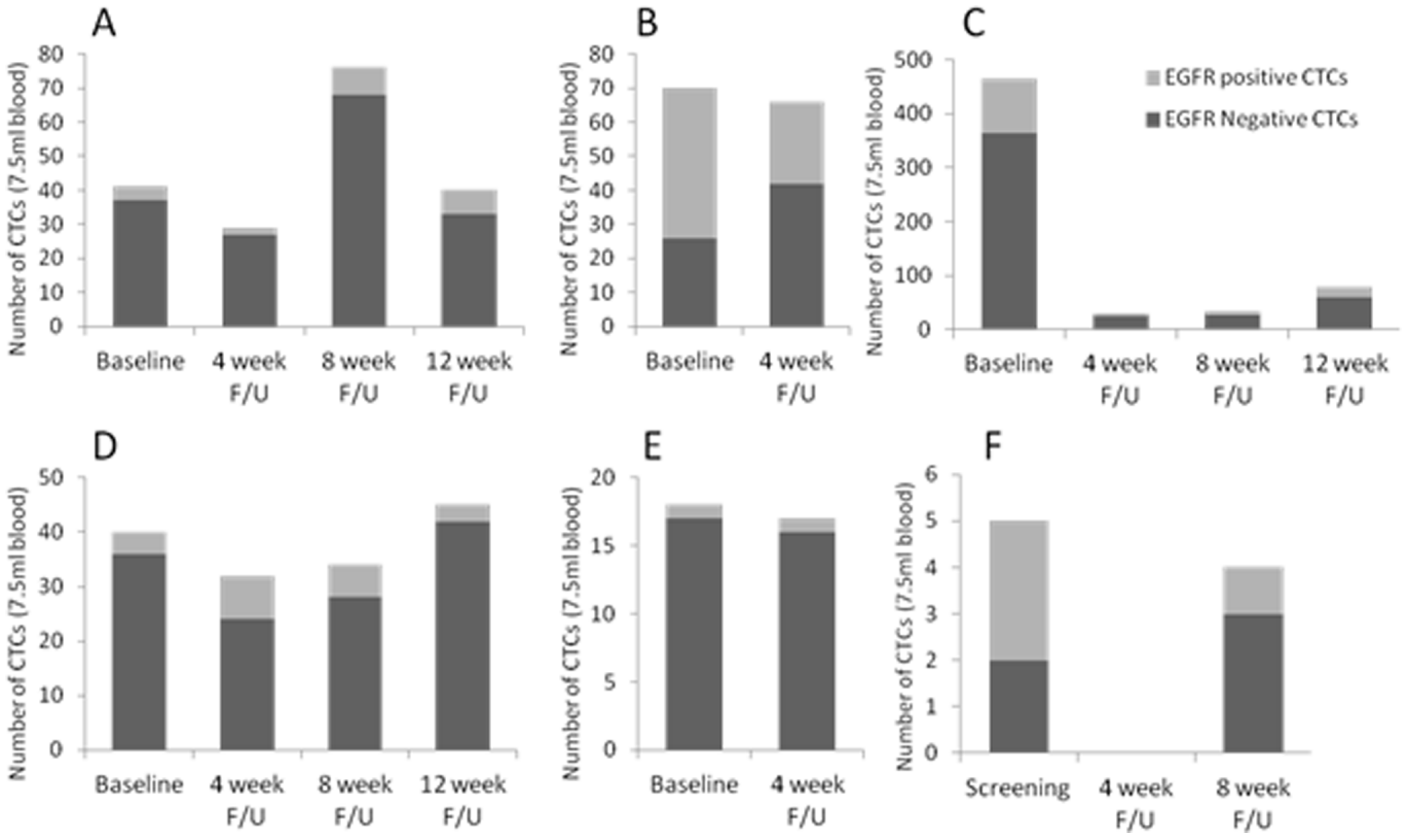
Six out of fourteen evaluable advanced breast cancer patients demonstrated a decrease in the quantity of CTCs in 7.5 ml blood following the first cycle of Lapatinib treatment. Graphs show CTC measurements and EGFR positivity at screening or baseline and at follow-up after Lapatinib treatment in each patient (A–F). Four patients also showed a decrease in EGFR positive CTCs after lapatinib treatment (A, B, C and F). Serial CTCs were taken with ethical approval (07/Q0401/20) and additional consent.

**Figure 3:**
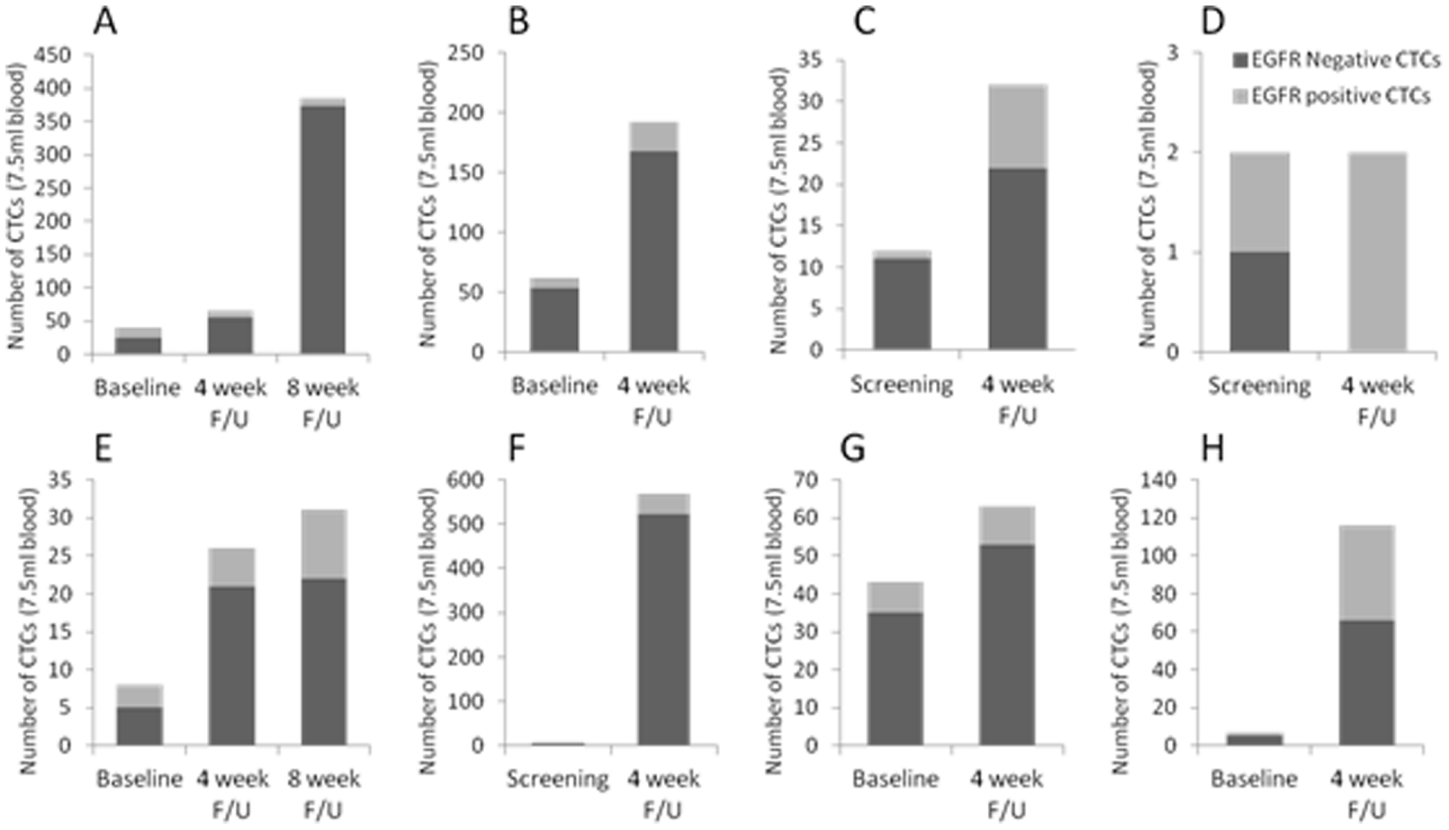
Eight out of fourteen evaluable advanced breast cancer patients demonstrated a consistent or increased quantity of CTCs in 7.5 ml blood following the first cycle of Lapatinib treatment. Graphs show CTC measurements and EGFR positivity at screening or baseline and at follow-up after Lapatinib treatment in each patient (A – H). Seven patients also showed an increase in EGFR positive CTCs after Lapatinib treatment (B, C, D, E, F, G and H). Serial CTCs were taken with ethical approval (07/Q0401/20) and additional consent.

Figures 2 and 3 demonstrate the changes in CTCs in those patients who had a decrease or increase in their CTCs following a 1^st^ cycle of lapatinib therapy, respectively. In those individuals who had a decrease in CTCs (Figure 2), 4 patients (A, B, C and F) had a decrease in their proportion of EGFR positive CTCs. Interestingly, in those patients who had an increase, 7 out of 8 patients (88%) had an increase in their EGFR positive CTCs after lapatinib therapy (B, C, D, E, F, G and H).

## Discussion

We wished to establish a role for targeted therapies based on a real time assessment of tumor sub-populations, and therein therapy directed at one of those sub-populations. In several patients we observed depletion of the EGFR positive pool of CTCs that were in some cases greater than decreases in their CTC population as a whole, we presume due to a direct consequence of lapatinib targeting this cellular group. This depletion however had no clinically discernable benefits in this trial setting and the study was stopped at the first analysis as no patients responded clinically. In aggregate, there was no evidence of response or clinical benefit with lapatinib in extensively pre-treated patients with HER2 non-amplified primary tumors and EGFR positive CTCs, regardless of effects on those CTC sub-populations. As expected toxicity was generally manageable although the 2 patients who withdrew rapidly with grade 3 diarrhea also had clinical evidence of rapid progression with a concomitant decline in their performance status.

We have recently drawn attention to issues with EGFR testing in breast cancer tumor specimens: in 810 patients less than 10% had breast cancers that stained positively [25]. Measurement of EGFR on CTCs appears more consistent and reproducible [15], thus lending itself to this study. Cancer is characterized by extensive heterogeneity at the cellular and molecular levels. There is a high degree of diversity between and within tumors as well as among cancer-bearing individuals, and all of these factors together determine the risk of disease progression and therapeutic resistance [26]. This insidious feature arises inevitably in almost all metastatic tumors and has broad significance for the outcome of the both the malignancy and the patient, because it confounds our understanding of the disease and intrinsically contributes to the tumor’s aggressiveness while posing an obstacle to the design of effective therapies. The classic view that heterogeneity arises as the result of a tumor’s ‘genetic chaos’ [27] and the more contemporary cancer stem cell (CSC) hypothesis tend to identify a single cell population as the therapeutic target [28]: the prevailing clone over time in the first case and the CSC in the latter [29]. However, there are increasing data that different tumor cell populations are unlikely to be simple bystanders [30], [31]. Rather, they can establish a complex network of interactions between each other and with the tumor microenvironment that eventually strengthens tumor growth and increases chances to escape therapy [32], [33].

While CTCs have the potential to be useful for patient selection, their specific targeting in distinction to the targeting of established metastatic tumor sites, is not known to be effective. Hence, the assessment of lapatinib's activity through a change in the level of the surrogate (CTC) could be considered by some of limited utility even if efficacy was observed. Other limitations here include choosing appropriate cut-offs in terms of the percentage pool of CTCs that were EGFR positive, and the fact that some patients had CTCs that were HER2 positive. The consistency of our data in patients with HER2 non-amplified tumors however (progression in all patients within 12 weeks) including 3 out of 14 (21%) patients who had no HER2 positive CTCs indicates that lapatinib monotherapy is not having any clinical benefits via either mechanism here. It is worthwhile mentioning that patients were recruited with at least one EGFR-positive CTC and perhaps a study recruiting higher levels in this population may enrich the data. Little attention has been paid thus far to understanding the role of tumor cell heterogeneity in therapeutic resistance and there is a lack of scientific studies to decipher how this heterogeneity is actively maintained through interclonal co-operativity [34]. It is important to understand the nature of this co-operativity, as abrogating such intercellular communication may represent a provocative tool in our arsenal to treat malignant tumors.

Large ongoing studies (eg. DETECT III, clinicaltrials.gov identifier NCT01619111) will help address the relevance of anti-HER2 targeted therapy (lapatinib versus standard of care) in patients with HER2-positive CTCs though we note here there were no responses to lapatinib even in patients with HER2 positive CTCs. This proof of concept study, in conjunction with the open label study from the Italian Study Group focusing on HER2 positive CTCs [21] should not be used to broadly suggest that CTC directed therapeutics is likely to fail, or that anti-HER2 treatments cannot be used in patients with metastatic breast cancer and HER2-negative primary tumors. Studies using trastuzumab are ongoing in similar settings. Both extracellular domain testing data showing increased conversion to positive serum HER2/*neu* status as disease progresses [35], [36] and CTC data suggests that HER2 status in individuals with metastatic breast cancer may change with time, especially in those who have received previous therapies for advanced disease [2], [4]. Future work will focus our attention on cellular sub-populations within an individual, to maximize benefit of targeted therapies.

## Supporting Information

Checklist S1 **CONSORT checklist of information for the trial.**
(DOC)

Protocol S1 **Protocol for the trial (Clinical trials.gov identifier: NCT00820924).**
(PDF)
