# Supplementary material for: The Efficacy of Lapatinib in Metastatic Breast Cancer with HER2 Non-Amplified Primary Tumors and EGFR Positive Circulating Tumor Cells: A Proof-Of-Concept Study
Source: PLoS One. 2013 May 7;8(5):e62543. doi: 10.1371/journal.pone.0062543 (PMC3647066; doi:10.1371/journal.pone.0062543)
Supplement: Protocol S1 — Protocol for the trial (Clinical trials.gov identifier: NCT00820924). (PDF) [file pone.0062543.s002.pdf]

Division: Worldwide Development
Retention Category: GRS019
Information Type: Protocol Amendment

**Title:**	A phase II open label, multicenter study to evaluate the efficacy and safety of daily dose of Lapatinib in advanced breast cancer patients with HER-2 non-amplified primary tumours and HER-2 positive circulating tumour cells or EGFR positive circulating tumor cells

Compound Number: GW572016

Effective Date: 02-OCT-2008

Protocol Amendment Number: 03

Description:

This multicenter open-label study is designed as a two-stage three-outcome phase II trial. The aim is to evaluate the efficacy and safety of daily dose of Lapatinib in advanced breast cancer patients with HER-2 non-amplified primary tumours and HER-2 or EGFR positive circulating tumour cells. Evaluation of HER-2 and EGFR *status* on circulating tumour cells will be performed by the means of the CellSearch equipment (Immunicon, Huntingdon Valley, PA, USA) and FISH method (PathVysion Kit -Abbott Laboratories).

Subject:

Breast Cancer; HER-2; EGFR, Circulating Tumour Cell (CTC); Lapatinib (GW572016)

Author:

Prof Angelo Di Leo
Oncologia Medica, Ospedale Misericordia e Dolce, Prato, Italy

Dr Valeria Quintily
Clinical Research Leader, CROM s.r.l, Verona, Italy

Dr Marc Buyse
Statistician, International Drug Development Institute, S.A., Louvain-la-Neuve, Belgium

Prof R C Coombes
Imperial College, 8th Floor, Cyclotron Building, Du Cane Road, London W12 ONN, UK

Copyright 2008 the GlaxoSmithKline group of companies. All rights reserved.
Unauthorised copying or use of this information is prohibited

Revision Chronology:

GM2007/00215/00	2007-APR-18	Original
GM2007/00215/01	2007-NOV-23	Amendment No.: 01<br><br>Country-specific: applicable only in United Kingdom
GM2007/00215/02	2008-FEB-28	Amendment No.: 02<br><br>Country-specific: applicable only in Italy
GM2007/00215/03	2008-OCT-02	Amendment No.: 03<br><br>Global amendment to incorporate hepatotoxicity information and protocol changes for monitoring, plus consolidate changes from previous country-level amendments.

CONFIDENTIAL GM2007/00215/03
LAP105594

**Sponsor Signatory:**	**Signature:**	**Date:**
**Dr Cristina Oliva**<br>Director, Oncology<br>Medicine Development<br>Centre GlaxoSmithKline	Cristina Oliva	October 2nd, 2008

SPONSOR INFORMATION PAGE

Clinical Study Identifier: 105594

Sponsor
GlaxoSmithKline
Stockley Park West
Uxbridge
Middlesex
UB11 1BT
Tel. +44(0)20 8990 9000
Fax. +44(0)208990 4321

Medical Monitor
Dr. Cristina Oliva
Stockley Park West
Uxbridge
Middlesex
UB11 1BT
Tel. +44 (0)20 8587 5453
Mobile +44 (0)7920567726
E mail: cristina.r.oliva@gsk.com

Regulatory Agency Identifying Number(s): EudraCT Number 2007-002155-17

INVESTIGATOR AGREEMENT PAGE

I confirm agreement to conduct the study in compliance with the protocol, as amended by this protocol amendment.

Investigator Name: ______________________________

____ ______________________

Investigator Signature Date

TABLE OF CONTENTS

	Page
ABBREVIATIONS	10
PROTOCOL SUMMARY	12
1. ENDPOINTS	14
2. INTRODUCTION	15
2.1. Background	15
2.2. Rational: Lapatinib and Breast Cancer	17
2.2.1. Rationale for Starting Dose in Monotherapy	22
3. OBJECTIVE(S)	23
3.1. Primary Objective	23
3.2. Secondary Objectives	23
3.3. Translational Research Objective	23
4. ENDPOINTS	23
4.1. Primary Endpoint	23
4.2. Secondary Endpoints	23
4.3. Translational Research Endpoints	24
5. INVESTIGATIONAL PLAN	24
5.1. Study Design	24
6. SUBJECT SELECTION AND WITHDRAWAL CRITERIA	27
6.1. Strata	27
6.2. Number of Subjects	27
6.3. Inclusion Criteria	27
6.4. Exclusion Criteria	29
6.5. Withdrawal Criteria	30
7. STUDY TREATMENTS	30
7.1. Investigational Product	30
7.2. Dosage and Administration	30
7.3. Dose Adjustment, Delays and Dose Modifications	31
7.3.1. Criteria for Evaluating Asymptomatic Cardiac events	33
7.3.2. Liver Chemistry Stopping and Follow Up Criteria	35
7.4. Packaging and Labeling	37
7.5. Handling and Storage	37
7.6. Treatment Assignment	37

7.7. Product Accountability 37
7.8. Treatment Compliance 38
7.9. Concomitant Medications and Non-Drug Therapies 38
7.9.1. Permitted Medications and Non-Drug Therapies 38
7.9.2. Prohibited Medications and Non-Drug Therapies 38
7.10. Treatment after the End of the Study 40
7.11. Treatment of Investigational Product Overdose 40

8. STUDY ASSESSMENTS AND PROCEDURES 40
8.1. Critical Screening Assessments 42
8.1.1. Procedures for blood and tumour tissue samples shipment to the central laboratory 43
8.2. Clinical Visit Schedules and Assessment during Study treatment and Follow up Periods 45
8.3. Efficacy 45
8.3.1. Methods, Scope and Schedules of Disease Assessment 45
8.4. Safety 49
8.4.1. Physical Examination 49
8.4.2. Vital Signs 49
8.4.3. ECOG PS 49
8.4.4. Clinical Laboratory Assessment 49
8.4.5. Criteria for Evaluating Asymptomatic Cardiac events 49
8.4.6. Toxicity Evaluation 50
8.4.7. 12 Lead Electrocardiogram and ECHO or MUGA 50
8.5. Positron Emission Tomography (PET) Sub-study 50
8.6. Long term remission (related to Amendment 01 – applicable only in UK) 50

9. ADVERSE EVENTS (AE) AND SERIOUS ADVERSE EVENTS (SAE) . . . 51
9.1. Definition of an AE: 51
9.2. Definition of a SAE 52
9.3. Disease-Related Events and/or Disease-Related Outcomes Not Qualifying as SAEs 54
9.4. Lack of Efficacy 54
9.5. Expected side effects 54
9.6. Time Period and Frequency of Detecting AEs and SAEs 55
9.7. Pregnancy 55
9.7.1. Time period for collecting pregnancy information 56
9.7.2. Action to be taken if pregnancy occurs 56

9.7.3. Prompt Reporting of Serious Adverse Events and Other Events to GSK 56
9.7.4. Regulatory Reporting requirements for SAEs 57

10. DATA MANAGEMENT 57

11. DATA ANALYSIS AND STATISTICAL CONSIDERATIONS 58
11.1. Hypotheses 58
11.2. Study Design Considerations 58
11.2.1. Sample Size Assumptions 58
11.2.2. Sample Size Sensitivity 59
11.2.3. Sample Size Re-estimation 59
11.3. Data Analysis Considerations 59
11.3.1. Analysis Populations 59
11.3.2. Analysis Data Sets 60
11.3.3. Treatment Comparisons 60
11.4. Interim Analysis 60
11.5. Key Elements of Analysis Plan 60
11.5.1. Efficacy Analyses 61
11.5.2. Safety Analyses 62

12. STUDY CONDUCT CONSIDERATIONS 63
12.1. Quality Control (Study Monitoring) 63
12.2. Quality Assurance 63
12.3. Study and Site Closure 64
12.4. Records Retention 64
12.5. Provision of Study Results and Information to Investigators 65
12.6. Provision of Study Results and Information to Investigator 65

13. PHARMACOGENETICS RESEARCH 65
13.1. Pharmacogenetics – Background 65
13.2. Scope of PGx Analysis 66
13.3. Eligibility Criteria Considerations for PGx Participation 66
13.4. Pharmacogenetics Samples 67
13.5. Confidentiality of Subject's PGx Data 67
13.6. Subject Withdrawal 68
13.7. PGx Data Management 68
13.8. Pharmacogenetics Analyses 68

14. REFERENCES 71

15. APPENDICES 74

15.1. Appendix 1: ECOG Performance Status Scale 74
15.2. Appendix 2: Cockcroft and Gault Method for Calculated Creatinine Clearance 75
15.3. Appendix 3: MATERIAL and METHOD 76
15.4. Appendix 4: PET Scan Sub-Study 81
15.5. Appendix 5: Liver Chemistry Stopping and Follow up Criteria 84
15.6. Appendix 6: Changes to the protocol 85

PROTOCOL AMENDMENT 03 88

ABBREVIATIONS

AE	Adverse Event
Akt	A proto-oncogene; also known as Protein Kinase B (PKB)
ALP	Alkaline phosphatase
ALT (SGPT)	Alanine Aminotransferase
AST (SGOT)	Aspartate Aminotransferase
ATP	Adenosine Triphosphate
BC	Breast Cancer
BID	Twice daily dose
BT474	Breast Ductal Tumour overexpressing ErbB2 cell line
CAP-CT	Chest, Abdomen and Pelvis- Computed Tomography
CBR	Clinical Benefit Rate
CNS	Central Nervous System
CR	Complete Response
CRF	Case Report Form
CT	Computed Tomography
CTC	Circulating Tumour Cells
CTC AE	Common Terminology Criteria for Adverse Event
DNA	Deoxyribonucleic Acid
DoCB	Duration of Clinical Benefit
DoR	Duration of Response
ECD	Extra Cellular Domain
ECHO	Echocardiogram
ECOG	Eastern Cooperative Oncology Group
EGF	Epidermal Growth Factor
EGFR	Epidermal Growth Factor Receptor
ER	Estrogen Receptor
ErbB1	EGFR; c-ErbB1; tyrosine kinase-type cell surface receptor HER1
ErbB2	EGFR; c-ErbB2; tyrosine kinase-type cell surface receptor HER-2
ErbB3	Tyrosine kinase-type cell surface receptor HER3
ErbB4	Tyrosine kinase-type cell surface receptor HER4
FISH	Fluorescence in situ Hybridization
GCP	Good Clinical Practice
GI	Gastro Intestinal
GSK	GlaxoSmithKline
HN5	Head and Neck ErbB1-overexpressing tumour cell line
IB	Investigator's Brochure
ICF	Informed Consent Form
IEC	Independent Ethics Committee
IHC	Immunohistochemistry
INR	International Normalized Ratio
INR/PT	Prothrombin time
IP	Interstitial Pneumonitis

ITT	Intent To Treat
LD	Longest Diameter
LVEF	Left Ventricular Ejection Fraction
MAPK	Mitogen-Activated Protein Kinase, also known as Erk1/2
MeDRA	Medical Dictionary of Regulatory Activities
MBC	Metastatic Breast Cancer
MR	Minor Response(s)
MRI	Magnetic Resonance Imaging
MUGA	Multiple Gated Acquisition
MSDS	Material Safety Data Sheet
NCI	National Cancer Institute
NSCLC	Non-Small Cell Lung Cancer
ORR	Overall Response Rate
OS	Overall Survival
pAkt	Phosphorylated Akt
pErk	Phosphorylated Erk
PD	Progression Disease
PFS	Progression-Free Survival
PI3K	PhosphotidylInositol-3-Kinase
PP	Per Protocol
PR	Partial Response
QD	Once daily dose
RECIST	Response Evaluation Criteria In Solid Tumours
RR	Response Rates
SAE	Serious Adverse Event(s)
SD	Stable Disease
TBR	Time to Best Response
TTP	Time To Progression
ULN	Upper of Normal Limit

Trademark Information

Trademarks of the GlaxoSmithKline group of companies
NONE

Trademarks not owned by the GlaxoSmithKline group of companies
AutoPrep
CellSave
CellSearch
CellSearch equipment
CellTracks
Immunicon
Iressa
MagNest
Tarceva
Veridex

PROTOCOL SUMMARY

Rationale: Despite recent progress in gene-expression profiling studies, the underlying biology of the various patterns of metastasis observed in different tumour types remains unclear. The detection and characterization of circulating tumour cells in cancer patients has provided important new information about the progression of metastatic events. This information has important implications for cancer prognosis and therapy.

This open label, multicenter phase II study is designed to evaluate overall tumour response rate in advanced breast cancer patients with HER-2 non amplified primary tumours with positive HER-2 or EGFR circulating tumour cells treated with the dual tyrosine kinase inhibitor GW572016 (Lapatinib).

The patients will be allocated into one of the following two strata:

Stratum 1) Italian study group: Advanced breast cancer patients with HER-2 non-amplified primary tumours and HER-2 positive circulating tumour cells

Stratum 2) UK study group: Advanced breast cancer patients with HER-2 non-amplified primary tumours and EGFR positive circulating tumour cells.

All of these patients will be treated with GW572016 (Lapatinib) which targets both HER-2 or EGFR receptors. A subgroup of patients enrolled into stratum 2 will be asked to participate in an additional sub-study using Positron Emission Tomography (PET) to quantify the potential early response to lapatinib treatment.

Study Design:

This study will be a multicenter open-label, phase II study to evaluate the efficacy and safety of daily dose of Lapatinib in advanced breast cancer patients with HER-2 non amplified primary tumours and positive HER-2 or EGFR circulating tumour cells.

Patients enrolled in this study will be treated with oral Lapatinib at the dose of 1,500 mg daily on day 1 to 28 every 4 weeks (q 4 weeks).

Patients will carefully be instructed on drug administration as far as Lapatinib oral intake is concerned. A daily dose of Lapatinib is six 250 mg tablets taken approximately at the same time each day. Lapatinib must be taken either at least 1 hour before or after meal.

Dose adjustment, modification and delays are permitted according to procedures described in Section 7.3.

Initially 16 patients in each stratum will be treated; if 1 to 3 responses are observed, 15 additional patients will be treated, up to a total of 62 subjects with 31 subjects in each stratum.

Objectives:

The primary objective of this study is to evaluate the efficacy of a daily dose of Lapatinib in advanced breast cancer patients with HER-2 non-amplified primary tumours and HER-2 or EGFR positive circulating tumour cell (CTC).

The secondary objectives of the study are:

- To evaluate antitumour activity of Lapatinib
- To determine the early response of Lapatinib on proliferation and the MAP kinase cascade by PET in a substudy in patients with EGFR positive CTCs only.
- To correlate response to Lapatinib with HER-2 and EGFR protein levels and amplification on CTCs as part of the tranaslational research
- To evaluate the safety of Lapatinib

1. ENDPOINTS

The primary endpoint for the analysis is to evaluate the overall response rate (ORR) according to RECIST criteria.

The secondary efficacy endpoints are:

- Clinical Benefit Rate (CBR = CR+PR+SD≥24 weeks)
- Duration of response (DoR) and of Clinical Benefit (DoCB)
- Time to Tumour Progression (TTP)
- Time to Best Response (TBR)
- Effects of Lapatinib on tumour proliferation and MAP kinase activity in a subset of patients with EGFR positive CTCs only as demonstrated on PET scan

The secondary safety endpoints are:

- Laboratory and non-laboratory toxicity

Safety will be measured using adverse events

The translational research endpoints

- Evaluation of biological effects on EGFR and HER-2 in circulating tumor cells (CTC).
- Comparison between CTC and tumor samples from the primary and/or from the metastatic site in terms of biological marker expression profiles.
- Correlation between EGFR both total and phosphorylated baseline levels evaluated on CTC and response to study drug.

2. INTRODUCTION

2.1. Background

Role of CTCs and HER-2/*neu* extra cellular domain (ECD) in breast cancer

Despite recent progress in gene-expression profiling studies, the underlying biology of the various patterns of metastasis observed in different tumour types remains unclear. The detection and characterization of circulating tumour cells in cancer patients has provided important new information about the progression of metastatic events. This information has important implications for cancer prognosis and therapy.

Circulating tumour cells (CTC) can currently be detected from blood samples of metastatic breast cancer (MBC) patients. Recently, Cristofanilli M et al, tested a laboratory assay for isolating and counting circulating tumour cells detected in blood samples [Cristofanilli, 2005; Cristofanilli, 2004]. The major finding of their studies is the potential opportunity to predict a response to systemic therapies as early as three to four weeks after treatment initiation. This might lead to an important change in the decision-making process for patients with metastatic disease. The same study has also shown that the number of CTC detected before initiation of first-line therapy in patients with MBC is highly predictive of progression-free and overall survival.

Currently the use of targeted therapies, such as trastuzumab or hormonotherapy, is based on the view that metastatic cells are linear descendants of primary tumour cells and have conserved the same biologic features. However a hallmark of Breast Cancer (BC) is its genetic instability. It appears that, despite the advent of targeted therapies, we are careless about the expression of targets, the clone selection process, and the clonal expansion of cells which do not express the target. Indeed, CTCs may show different properties from primary tumour cells and biological characterization of CTCs might lead to the identification of appropriate treatments for advanced breast cancer patients. In a recent study published by Meng S. et coll., it was reported that 9 out of 24 (37.5%) advanced breast cancer patients whose primary tumour was HER-2 negative (FISH negative) acquired HER-2 gene amplification in their circulating tumour cells. Of note, 4 of the 9 patients were treated with trastuzumab-based therapies and 3 of the 4 treated patients had a remarkable clinical response although heavily pre-treated for advanced disease [Meng, 2004].

The HER-2/*neu* oncogene and its p185 receptor protein are indicators of a more aggressive form of breast cancer. The HER-2/*neu* extracellular domain (ECD) is shed from cancer cells into the circulation and is measurable by immunoassay. The prevalence of increased ECD serum levels in patients with primary breast cancer varied between 0% and 38% (mean 18.5%); whereas in metastatic disease the range was 23% to 80% (mean 43%) [Carney, 2004; Carney, 2003]. These data suggest that the prevalence of HER-2 protein overexpression might be higher in metastatic disease than in early disease.

Different from tissue testing that is a one-time event, monitoring ECD circulating levels provides a real-time assessment of the HER-2/*neu* status. Lipton A. et coll. evaluated whether patients with metastatic or locally advanced breast carcinoma, who have negative serum HER-2/*neu* status at the initiation of first-line hormone therapy, convert to positive serum HER-2/*neu* status at the time of disease progression and whether serum HER-2/*neu* serum conversion is associated with response to therapy and overall survival. They found that conversion to positive serum HER-2/*neu* status occurred in approximately 25% of patients and multivariate analysis revealed that conversion to positive serum HER-2/*neu* status was an independent factor associated with a poor survival rate [Lipton, 2005].

The ECD data, along with the CTC data reported by Meng et al, suggest that HER-2 status in MBC patients might change with time, particularly in those patients who have received previous systemic therapies for advanced disease.

Epidermal growth factor receptors (ErbB family) in breast cancer: focus on EGFR and HER-2

The ErbB/HER protein kinases, which include the epidermal growth factor receptor and HER-2, are among the most investigated treatment targets in the field of breast cancer research [Hanahan, 2000].

Expression of ErbB1 and/or overexpression of ErbB2 are reported in different epithelial malignancies, where they promote tumor cell growth/survival. Moreover, their expression correlates with a poor clinical outcome in some epithelial tumors. The phosphorylated tyrosine residues serve as docking sites for Src-homology 2 and phosphotyrosine binding domain-containing proteins that link activated ErbB receptors to downstream cell proliferation (mitogen-activated protein kinase [MAPK]) and survival (phosphotidylinositol-3-kinase [PI3K]) pathways. Hence, the key- role played by ErbB1 and ErbB2 in promoting growth and survival of various solid tumor types makes them attractive therapeutic targets [Yarden, 2001; Klapper, 2000].

The human epidermal growth factor receptor (HER-1) is the prototype of a family that consists of four known members (EGF receptor/HER-1, neu/erbB2/HER-2, erbB3/HER-3, and erbB4/HER-4). These receptor tyrosine kinases are characterized by an extracellular ligand-binding domain, an internal kinase domain, and a carboxyl-terminal domain that contains multiple tyrosine residues. Dysregulated EGFR expression, ligand production and signalling have been implicated in the development of various solid tumors. Recently, our understanding of EGFR and the other ErbB family receptors has grown rapidly and a substantial amount of data indicate that EGFR activation is associated with tumor cell proliferation, migration, angiogenesis, invasion, differentiation, adhesion, and inhibition of apoptosis.

Among the HER family members, HER-2 is most closely related to HER-1 and it has been found to be amplified in 10-35% of human breast carcinomas [Biscardi, 2000]. The HER-2/neu gene, when overexpressed, transforms normal cells into cancer cells and is thought to be involved in tumor initiation and early stages of progression. The HER-2 protein interacts with other HER family members, allowing HER-2 to serve as a co-receptor and to facilitate signal transduction via a heterodimer complex that is formed

after ligand binding. There is no known ligand for HER-2 itself. This suggests that the primary role of HER-2 is to modulate signals after ligand binding to other HER-family receptors [Yarden, 2001]. ErbB2 containing heterodimers exert potent growth and survival effects. Therefore, simultaneous inhibition of ErbB2 and ErbB1 is an appealing therapeutic strategy.

The role of lapatinib in those patients in whom there is evidence of EGFR expression in the absence of HER-2 is poorly understood. Although there have been reports of EGFR positive and HER-2 (3+) negative patients responding to gefitinib or lapatinib, these are infrequent and poorly documented. Further, there have been no studies principally designed to elucidate the changes in EGFR expression or activation (phosphorylation) after inhibitor therapy.

Therefore this study is designed to investigate the potential clinical activity and safety of Lapatinib in advanced breast cancer patients with HER-2 non-amplified primary tumours with positive HER-2 or EGFR CTCs.

2.2. Rational: Lapatinib and Breast Cancer

Lapatinib acts as a dual inhibitor of both EGFR and ErbB2 tyrosine kinase activity. As a member of the 4-anilinoquinazoline class of kinase inhibitors, lapatinib is thought to react with the ATP binding site of EGFR/ErbB2, resulting in inhibition of autophosphorylation and subsequent proliferative signaling [Shewchuk, 2000].

The ErbB family consists of four closely related growth factor receptor tyrosine kinases. The family is comprised of ErbB1 (EGFR/HER), ErbB2 (HER2/neu), ErbB3 (HER3), and ErbB4 (HER4). All members of the ErbB family share a common extracellular ligand-binding domain, a single membrane-spanning region and a cytoplasmic tyrosine kinase domain (reviewed in [Yarden, 2001; Burgess, 2003]). A ligand for ErbB2 has not been identified, while ErbB3 lacks tyrosine kinase activity. Ligand binding to EGFR, ErbB3 or ErbB4 induces these inactive monomers to undergo an array of homo-or heterodimerization with other members of the ErbB family. ErbB2 is the preferred heterodimeric partner for all ErbB receptors, resulting in a complex that is endocytosed at one half to one third the rate of other EGFR dimers [Graus-Porta, 1997; Hendriks, 2003]. ErbB dimerization leads to receptor autophosphorylation and subsequent activation of the tyrosine kinase domain. The signaling characteristics of the ErbB family are thought to be strongly interdependent.

EGFR expression by tumour cells has been linked with aggressive tumour growth, disease progression, poor survival, and poor response to therapy. Over-expression of EGFR has been reported in a number of epithelium-derived carcinomas including head and neck, colorectal, lung, esophageal, gastric, and breast carcinoma. In a similar manner, ErbB2 has been reported to be over-expressed in 15-30% of invasive ductal breast cancer and has been associated with increased proliferation, poor clinical outcome, and altered responsiveness to various adjuvant therapies [Allred, 1992]. Activation of either EGFR or ErbB2 initiates a series of signaling cascades that includes mitogen-activated protein kinase (MAPK), phosphoinositide 3-kinase (PI3K), Akt, and p70S6K.

Lapatinib has shown to be a potent and selective dual inhibitor of EGFR and ErbB2 tyrosine kinase activity with IC_{50} values of 10.2 and 9.8 nM, respectively [Rusnak, 2001]. Lapatinib has demonstrated selective growth inhibition of human cell lines (head and neck, breast, and gastric) *in vitro* (IC_{90} values <2.26 μM or 1313 ng/mL) with no outgrowth observed up to 18 days following cessation of treatment. Growth inhibition corresponded with the ability of lapatinib to inhibit phosphorylation of Akt. These studies suggested that inhibition of EGFR by lapatinib resulted preferentially in cell growth arrest, while inhibition of ErbB2 led to cell growth arrest and apoptosis after 72 hours. Treatment with lapatinib leads to arrest of tumour cell growth and/or apoptosis, even in the presence of saturating concentrations of epidermal growth factor (EGF). Treatment of tumour xenografts resulted in inhibition of activation of EGFR, erbB2, Erk1/2, and Akt [Xia, 2002].

Antitumour activity was observed in nonclinical studies. The ability of lapatinib to inhibit the growth of EGFR-over-expressing cell lines ($IC_{50} \leq 0.16$ μM) was observed to be equal to that of other EGFR inhibitors being tested in clinical trials (e.g. gefitinib/Iressa or erlotinib/Tarceva). With the exception of ErbB4, lapatinib was >300-fold more selective towards ErbB2 and EGFR kinase inhibition than to other kinases tested. Lapatinib demonstrated potent growth inhibition of human breast ductal (BT474) and head and neck (HN5) tumour xenografts in mice. A dose response inhibition was observed in both models receiving lapatinib (30 or 100 mg/kg twice daily orally for 21 days). Complete inhibition of tumour growth was seen in mice receiving 100 mg/kg [Rusnak, 2001].

In nonclinical studies lapatinib has been shown to inhibit Erk1/2 and Akt phosphorylation (pErk and pAkt) in both EGFR and erbB2-expressing cell lines (BT474 and HN5). The ability of lapatinib to inhibit pAkt was associated with a 23-fold increase in the percentage of cells undergoing apoptosis compared to control cells. Similarly, lapatinib treatment of BT474 and HN5 xenografts in mice also resulted in inhibition of Erk1/2 and Akt phosphorylation. These results suggested that lapatinib treatment of EGFR/erbB2 expressing tumours could lead to inhibition of downstream signalling events [Xia, 2002].

A study of human breast cancer cell lines that over-express EGFR or erbB2 (SUM102, SUM149, SUM185, and SUM225) reported that treatment with lapatinib resulted in inhibition of cell proliferation that was associated with inhibition of Erk phosphorylation. Inhibition of Erk phosphorylation also correlated with radiosensitization of cell lines pretreated with lapatinib [Zhou, 2003].

Toxicology

A range of toxicology studies has been conducted to support the oral administration of lapatinib to humans. Repeated oral dose toxicity studies have been completed in rats and dogs for up to 6 and 9 months, respectively. The effects of lapatinib on fertility in the rat and embryofetal development in the rat and rabbit have been investigated. A range of genetic toxicity studies has been performed in vitro and in vivo. The significant findings from the toxicology studies are summarized below.

Following single oral administration, lapatinib was well-tolerated by both CD-1 mice and Wistar Han rats at doses up to 2000 mg/kg. Treatment-related findings consisted of reversible changes in body weight and body weight gain as well as reversible GI effects.

A 13-week oral dose ranging pilot carcinogenicity study in mice showed that treatment with lapatinib at doses up to 200 mg/kg/day was generally well tolerated. Microscopic changes attributable to treatment with lapatinib were noted in the liver and preputial gland of males and large intestines (caecum and colon) of males and females.

Administration of lapatinib to rats and dogs for up to 6 months or 9 months resulted primarily in exaggerated pharmacologic effects and organ toxicities generally associated with degenerative and/or inflammatory epithelial changes (GI tract and accessory digestive organs, skin, mammary gland, liver and prostate). Other treatment-related effects included clinical signs, decreased body weight and food consumption, organ weight changes and alterations in clinical pathology parameters. Following the recovery period, treatment-related changes were either significantly improved or completely reversed.

Fetal malformations were not observed when lapatinib was administered to pregnant female rats or rabbits during the period of major organogenesis. At maternally toxic doses, lapatinib treatment was associated with growth retardation and developmental variations. There has been no evidence of genotoxicity or clastogenicity in mutagenic studies.

Pharmacokinetics

The pharmacokinetics of lapatinib are similar in healthy volunteers and patients, demonstrating that oral absorption is incomplete, highly variable, and sometimes delayed. After dosing, plasma concentrations rise to a peak at approximately 4 h and thereafter decline with measured half-lives averaging up to 14 h. However, accumulation with daily dosing achieves steady state in 6-7 days, which suggests a true elimination half-life on the order of 24 h. Administration of the same daily dose in a BID schedule results in 2-fold greater systemic exposure than a QD schedule. Despite this inconsistency, systemic exposure generally increases with increasing dose. Absorption is increased by ingestion with food. Elimination of lapatinib is predominantly through metabolism by CYP3A4/5 with negligible renal excretion. Significant changes in systemic exposure to lapatinib result from co-administration of drugs that are potent inhibitors or inducers of CYP3A.

In a Phase I trial (EGF10009), subjects receiving a combined regimen of paclitaxel 175mg/m2 intravenously every 3 weeks and lapatinib 1500mg orally once daily, plasma concentrations (AUC) of paclitaxel were increased approximately 25% and plasma concentrations (AUC and Cmax) of lapatinib were increased approximately 33% and 53%, respectively. These results are consistent with in vitro data showing that lapatinib inhibits CYP3A4 and 2C8-mediated metabolism of paclitaxel, and published data [Beluz-Riche, 2002] suggesting that paclitaxel inhibits CYP3A4, a major pathway of lapatinib metabolism. These results are consistent with the strong dependence of lapatinib elimination on CYP3A metabolism. Co-administration of lapatinib with-potent inhibitors or inducers of CYP3A4 is not recommended.

Safety Profile of Lapatinib

Preliminary serious adverse events (SAE) data are available for ongoing lapatinib studies (all phases) up to a cut-off date of 22 June 2005. At the time of the SAE cut-off, over 3109 subjects have received lapatinib in phase I, II and III studies. A total of 2620 SAEs were reported from 543 individual subjects. The most frequently reported SAE was diarrhoea, with a total of 123 reports, 66 of which were assessed as related to investigational product. Pyrexia, dyspnoea, vomiting and nausea were also amongst the most frequently reported SAEs regardless of relatedness. Overall, 32% of the SAEs reported were assessed as related to investigational product by the investigator. The majority (81%) of reports of neutropenia were from combination of lapatinib with paclitaxel, which is known to be associated with neutropenia. All but 2 of the remaining reports occurred in studies where subjects received combination therapy with chemotherapeutic agents such as capecitabine, cisplatin, irinotecan, leucovorin or fluorouracil, all of which are associated with neutropenia.

Left ventricular ejection fraction (LVEF) has been evaluated using MUGA scans or echocardiograms (Echos) during lapatinib phase I, II and III trials. As of 22 June 2005 a total of 38 subjects on the lapatinib program have experienced a total of 39 events of decreased LVEF, giving an approximate incidence for this event of 1.3% [Perez, 2006]. The majority (65%) of decreases in ejection fraction were detected by week 8 of treatment, i.e. at the week 8 MUGA/Echo evaluation. Three events (two for one subject) remain blinded, and three subjects did not receive lapatinib. For the 33 subjects who received lapatinib, where outcome was reported, 10 events resolved or improved on discontinuation of lapatinib (as demonstrated by repeat MUGA/Echo), 8 events resolved or improved whilst lapatinib was continued, and 12 events were unresolved. For 3 events outcome was unknown at the time of reporting. Three of the 38 subjects with decreased LVEF presented with symptoms of breathlessness, dyspnoea, and signs of cardiac failure, and were found as part of their examination to have LVEF reduction. Two of these subjects had co-morbidities which could have contributed to the symptoms (one with pericardial and pleural effusions; one with diabetes, coronary artery disease, hypertension and hypercholesterolemia). The third subject had previously received trastuzumab. The events resolved on discontinuation of lapatinib and were assessed as lapatinib-related by the investigator. Thirty-two subjects were asymptomatic and the events were discovered on routine study MUGA/Echo examination. Twenty-four of these asymptomatic events were assessed by the investigator as related to treatment with lapatinib, 7 events were assessed as unrelated and 2 events had unknown causality at the time of reporting. Approximately half of the subjects in the lapatinib trials had previously received treatment with either anthracyclines and/or trastuzumab, both of which have been associated with cardiotoxicity. This exposure therefore increases the risk of cardiotoxicity in the lapatinib subjects irrespective of any cardiotoxicity attributed to lapatinib.

A more recent analysis of patients treated with lapatinib to date revealed that approximately 1.6% of patients in 43 studies had a decrease in LVEF. The frequency of symptomatic LVEF decrease was 0.2%. A sub-analysis performed in patients who had previously received an anthracycline-based therapy but no trastuzumab (Group 1, n=698), patients who previously received anthracycline-based therapy followed by trastuzumab (Group 2, n= ~700) and patients with mostly non-breast tumours who were treated with

neither anthracyclines or trastuzumab (Group 3, n=2200) indicated that there is a consistent frequency of LVEF decrease of approximately 1.4-1.6% and symptomatic events occurred at a frequency of 0.1 – 0.3%. The cardiac events were generally reversible and of short duration with a median of approximately 13 weeks. These encouraging results are in the process of being confirmed. (GSK data on File).

As of 04 December 2005, nine pulmonary events have been reported: five subjects experienced interstitial lung disease and four subjects experienced pneumonitis. Given current enrolment of 3876 subjects in the lapatinib program, this gives an approximate incidence of 0.2% for ‘pneumonitis-type’ events. Six subjects were participating in combination studies and one of these subjects did not receive lapatinib. The remaining three reports were from lapatinib monotherapy studies. Five subjects (55%) who experienced pulmonary events recovered. Three of these five subjects required treatment, commonly methylprednisolone or prednisolone. For the remaining four subjects, two events were fatal, one event was ongoing at the time of the subject’s death due to disease progression, and one event was ongoing at the time of reporting.

As part of ongoing pharmacovigilance by GlaxoSmithKline, a review of all hepatobiliary events reported across the entire lapatinib clinical development programme has been performed. Two hundred sixteen reports of hepatic events were retrieved from the GSK safety database as of 31 December 2007 regardless of source (clinical trials, spontaneous/marketed use data). In 39 of the 216 cases, a causal association to lapatinib could not be ruled out: 38.5% (15/39) of these subjects received lapatinib monotherapy, 53.8% (21/39) of subjects received lapatinib in combination with other chemotherapies, such as capecitabine, and 3 cases were still blinded.

A total of 13 deaths were identified which contained hepatobiliary events. In 3 of these cases, an association with lapatinib could not be excluded. The remaining 10 cases were confounded by the patients underlying condition (progressive disease and/or progression of pre-existing liver metastases).

Based on an additional sub-analysis, of 18 clinical studies of lapatinib in breast cancer, using Hy’s Law (defined as AST or ALT >3 x ULN, and total bilirubin >2 x ULN, with no initial findings of cholestasis i.e.: ALP <2 x ULN) as a predictor for potential drug induced liver injury, the liver injury associated with lapatinib seems to be the result of a prolonged exposure to the drug. All the subjects whose events potentially met Hy’s Law received study medication for three months or longer. The majority of these cases appeared reversible. Most patients experienced a decline in liver enzymes with drug cessation.

Based on the results of this review, GSK concluded a causal relationship between hepatobiliary disorders (specifically transaminase elevations) and lapatinib cannot be excluded. As a consequence, hepatotoxicity was added to the core safety information (CSI) for lapatinib. In addition, for ongoing clinical trials, the monitoring interval for hepatic function has been increased to every 4-6 weeks during treatment, and stopping rules have been added for severe hepatic events. Lapatinib dosing should be discontinued if changes in liver function are severe and patients should not be retreated.

2.2.1. Rationale for Starting Dose in Monotherapy

Results from phase I studies support the use of 1500 mg po daily as achieving biologically active concentrations and target pathway inhibition with an acceptable toxicity profile.

Based on pre-clinical and phase I data, [Blackwell, 2005; Burnstein, 2005] performed two phase II trials in trastuzumab-refractory metastatic breast cancer. The primary objectives of these studies were the assessment of safety and efficacy of lapatinib monotherapy at the dose of 1500 mg daily. Preliminary side-effect data included events of rash, diarrhea, anorexia, nausea, vomiting and weight loss. Study drug-related adverse events were all grades 1-2 except for one grade 3 rash.

In this patient population lapatinib administered orally at 1500 mg daily provided evidence of efficacy with a clinical benefit rate of 22%. The results of these trials suggest activity of lapatinib in trastuzumab-pretreated patients.

Gomez HL et al. conducted a phase II randomized study of lapatinib as first-line treatment for patient with HER-2 FISH-amplified metastatic breast cancer. One hundred and thirty-eight patients were randomly assigned to receive lapatinib 1500 mg as a single daily dose (QD) or 500 mg twice daily (BID). A total of 138 patients were treated with lapatinib for a median of 17.6 weeks. The overall response rate (complete response [CR] plus partial response [PR]) was 24% in the intent-to-treat population, and 31% of patients derived clinical benefit (CR, PR, or stable disease for _ 24 weeks). The median time to response was 7.9 weeks, and the progression-free survival rates at 4 and 6 months were 63% and 43%, respectively. The most common lapatinib-related adverse events (AEs) were diarrhea, rash, pruritus, and nausea, and these events were primarily grade 1 or 2. There were no significant differences in clinical activity or the AE profile between the dosing schedules. The authors concluded that lapatinib appeared well tolerated and showed evidence of activity as first-line treatment for women with HER-2 amplified advanced breast cancer [Gomez, 2008].

Furthermore, in study EGF 100151, a Phase III trial evaluating treatment with capecitabine alone or in combination with lapatinib in 324 patients with HER2-positive, locally advanced or MBC refractory to trastuzumab, the median time to progression (TTP) of patients in the combination arm (oral lapatinib 1250 mg QD plus capecitabine 2000 mg/m2/day on days 1–14 every 3 weeks) was significantly higher than that in the group receiving capecitabine alone (2500 mg/m2/day on days 1–14 every 3 weeks); median TTP of 36.9 weeks with combination therapy vs 19.7 weeks with monotherapy ($p < 0.001$, HR = 0.51, CI = 0.35–0.74) [Geyer, 2006].

Based on presented data this study hypothesizes that approximately 25% of patients with HER-2 non-amplified primary breast cancer acquire a HER-2 amplified *status* in the metastatic setting, particularly after treatment with systemic therapies for advanced disease. This multicenter phase II clinical trial attempts to demonstrate that a simultaneous inhibition of ErbB2 and ErbB1 is clinically active in advanced breast cancer patients with HER-2 positive or EGFR positive CTCs and HER-2 non-amplified primary tumours.

3. OBJECTIVE(S)

3.1. Primary Objective

The primary objective of this study is to evaluate the efficacy of a daily dose of lapatinib in advanced breast cancer patients with HER-2 non-amplified primary tumours with HER-2 or EGFR positive circulating tumour cells.

3.2. Secondary Objectives

The secondary objectives of this study are as follows:

- To evaluate antitumour activitiy of lapatinib
- To evaluate the safety of lapatinib
- To determine the early response of lapatinib on proliferation and the MAP kinase cascade by PET in a substudy in patients with EGFR positive CTCs only.

3.3. Translational Research Objective

The translational research objective is to correlate response to lapatinib with HER-2 and EGFR protein levels and amplification evaluated on CTCs before starting treatment. A centralized review of the HER-2 status on the primary tumour will retrospectively be performed.

4. ENDPOINTS

4.1. Primary Endpoint

The primary endpoint for the analysis is to evaluate the overall response rate (ORR) according to RECIST criteria.

4.2. Secondary Endpoints

The secondary efficacy endpoints are:

- Clinical Benefit Rate (CBR = CR+PR+SD≥24 weeks)
- Duration of response (DoR) and of Clinical Benefit (DoCB)
- Time to Tumour Progression (TTP)
- Time to Best Response (TBR)
- Effects of lapatinib on tumour proliferation and MAP kinase activity in a subset of patients with EGFR positive CTCs as demonstrated on PET scan (Stratum 2 only)

The secondary safety endpoints are:

- Laboratory and non-laboratory toxicity

Safety will be measured using adverse events

4.3. Translational Research Endpoints

The translational research endpoints are:

- Evaluation of biological effects of lapatinib on EGFR and HER-2 in circulating tumor cells (CTC)
- Comparison between CTC and tumor samples from the primary and/or from the metastatic site in terms of biological marker expression profiles
- Correlation between EGFR both total and phosphorylated baseline levels evaluated on CTC before starting treatment and response to Lapatinib

5. INVESTIGATIONAL PLAN

5.1. Study Design

This study will be a multicenter open-label, phase II study to evaluate the efficacy and safety of daily dose of lapatinib in advanced breast cancer patients with HER-2 non amplified primary tumours. The patients will be allocated into one of the following two strata:

Stratum 1: Italian Study Group: Advanced breast cancer patients with HER-2 non-amplified primary tumours and HER-2 positive circulating tumour cells

Stratum 2: UK Study Group: Advanced breast cancer patients with HER-2 non-amplified primary tumours and EGFR positive circulating tumour cells.

The study will be conducted at approximately 15 centres in two countries, Italy and the UK. The HER-2 positive CTCs will be tested by the Italian group and EGFR CTCs will be tested by the UK group.

Patients enrolled in this study will be treated with oral lapatinib at the dose of 1500 mg daily on day 1 to 28 every 4 weeks (q 4 weeks).

Assigned therapy will be dispensed to the patient on day 1 after it has been confirmed that the patient meets all eligibility criteria and all screening assessments have been completed and the results reviewed. Patients enrolled in the study will return to the site every 4 weeks for additional supplies of lapatinib.

Treatment will be continued until disease progression, unacceptable toxicity or death whichever occurs first.

Patients will carefully be instructed on drug administration as far as lapatinib oral intake is concerned. A daily dose of lapatinib is six 250 mg tablets taken approximately at the same time each day. Lapatinib must be taken either either at least 1 hour before or after meal.

Dose adjustment, modification and delays are permitted according to procedures described in Section 7.3.

If vomiting occurs after lapatinib intake, the patient should be instructed not to retake the dose. If vomiting persists, then the patient should contact the investigator.

The study will consist of three study periods: (I) screening; (II) treatment and (III) follow-up.

1. Screening Period:

The screening period will include time from the timepoint in which eligible subjects sign the informed consent form until confirmation of eligibility from central laboratory based on CTCs ErbB1 and ErbB2 status. The screening phase can last up to 28 days prior to 1st dose.

2. Treatment Period:

The treatment period will start from the 1st study drug dose intake until disease progression, unacceptable toxicity or death whichever occurs first.

3. Follow up Period:

This period will commence 28 days from the time of last study drug dose intake. Post-study survival follow up status will be collected every 12 weeks and can be collected by phone if the patient cannot attend the hospital.

This study is also designed to examine the early effects of lapatinib on tumour proliferation and MAP kinase activity using PET. This sub-study will only be conducted in the UK centre. Patients must satisfy the additional eligibility criteria in order to enter this sub-study. The PET sub-study is outlined in Appendix 4.

Figure 1 Study Flow

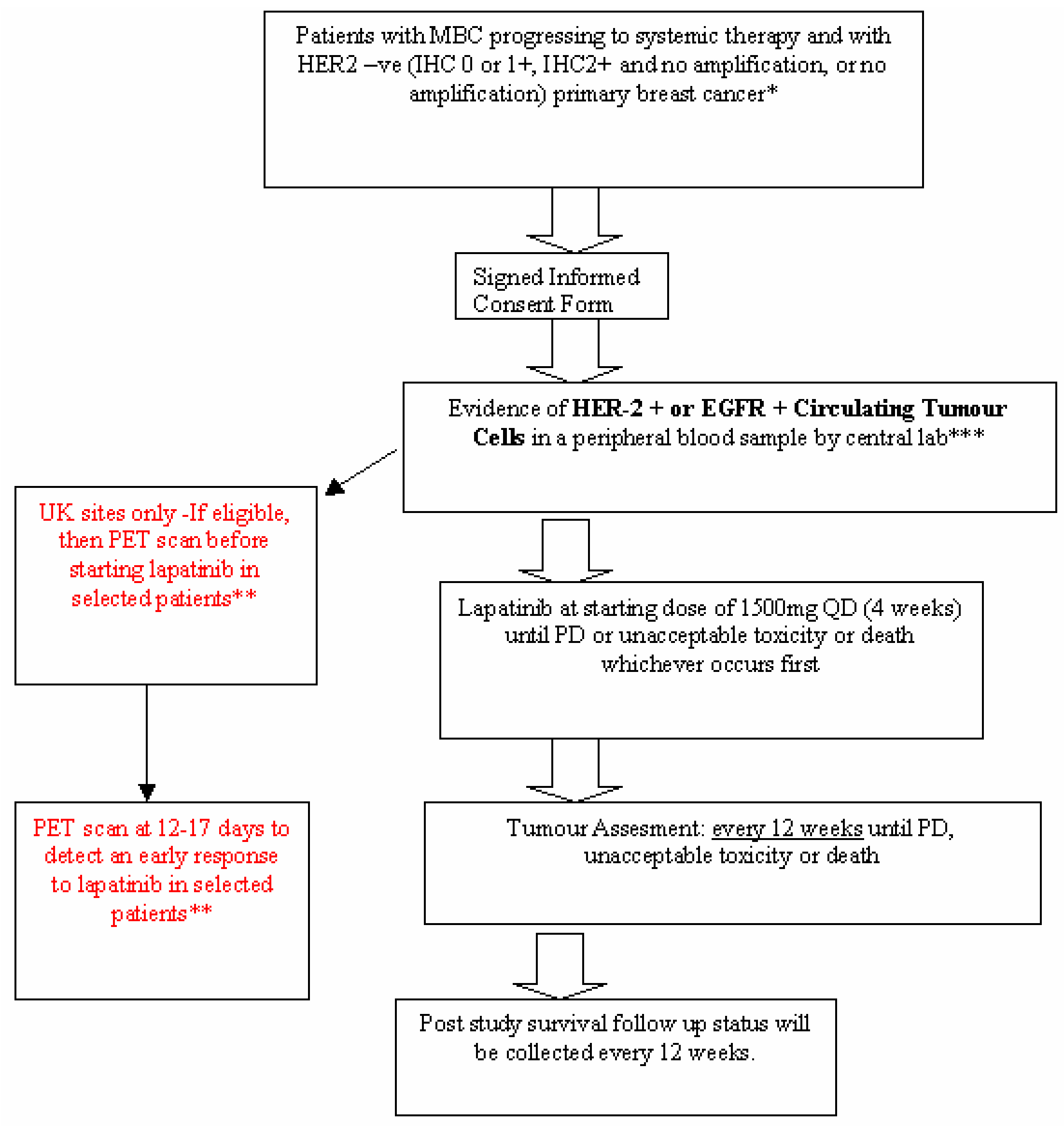

**PET sub-study will only be performed on paitents with EGFR positive CTCs who have met the additional entry criteria, see Appendix 4 for details.
***Central laboratory: Translational lab at Prato Hospital for Italian Study Group and Hammersmith Hospital for the UK Study Group

Supplementary study conduct information not mandated to be present in this protocol is provided in the Appendix 3.

6. SUBJECT SELECTION AND WITHDRAWAL CRITERIA

6.1. Strata

The subjects will be allocated into one of the following two strata:

Stratum 1) Italian study group: Advanced breast cancer patients with HER-2 non-amplified primary tumours and HER-2 positive circulating tumour cells

Stratum 2) UK study group: Advanced breast cancer patients with HER-2 non-amplified primary tumours and EGFR positive circulating tumour cells.

6.2. Number of Subjects

The study is designed as a two-stage three-outcome phase II trial in each stratum.

1st Stage:

Initially 16 eligible subjects in each stratum will be treated, and

- if no response is observed, the drug will be declared to be insufficiently active
- if 4 or more responses are observed, the drug will be declared to be sufficiently active
- if 1 to 3 responses are observed, study will proceed to the second stage as follows.

2nd Stage:

Treat up to 15 additional subjects in each stratum (up to 62 patients in total), and

- if up to 2 responses in total are observed, the drug will be declared to be insufficiently active
- if 4 or more responses in total are observed, the drug will be declared to be sufficiently active
- if 3 responses in total are observed, no final conclusion can be drawn from this study

Note that after accrual of 16 subjects in each stratum, the trial can terminate as soon as 4 responses are observed in that stratum. It is considered lapatinib has sufficient activity in the target population that when 4 or more responses are observed because it is expected that lapatinib should have no activity in HER-2 negative breast tumour.

6.3. Inclusion Criteria

This study attempts to prove the concept that patients who would never be treated with anti-Her-2 compounds can indeed be considered for this option. Specific information regarding warnings, precautions, contraindications, adverse events, and other pertinent

information on the investigational product that may impact subject eligibility is provided in the latest version of the lapatinib Investigator's Brochure or product labels.

Subjects eligible for enrolment in the study must meet all of the following criteria:

1. Signed written informed consent
2. Female patients, age ≥ 18 years
3. Histologically confirmed diagnosis of HER-2 negative (i.e. no gene amplification by FISH or IHC 2+ and no amplification by FISH or IHC 0/1+) infiltrating primary breast cancer.
4. Evidence of EGFR positive circulating tumour cells or HER-2 positive (≥50% CTCs or FISH positive) in a peripheral blood sample taken at screening visit.
5. Metastatic disease with at least one measurable lesion as per RECIST criteria classification [Therasse, 2000]
6. No brain metastasis requiring local therapy. Stable or asymptomatic subjects are allowed.
7. Availability of tumour block or at least six unstained 4 μ sections of the primary tumour to be sent to the central lab after eligibility confirmation for HER-2 central testing by FISH.
8. Eastern Cooperative Oncology Group (ECOG) score for performance status 0 to 3 (Appendix 1)
9. Life expectancy > 12 weeks
10. Adequate baseline organ function at screening visit:
    - Hematologic function:
        - ANC ≥ 1,000 / mm^3,
        - PLT ≥ 100,000 / mm^3 ,
        - Hb ≥ 10g / dL (after transfusion if needed)
    - Hepatic function:
        - GOT/GPT/alkaline phosphatase ≤ 3 ULN without liver metastases or ≤ 5 ULN if documented liver metastases;
        - total bilirubin: ≤1.5 ULN unless due to Gilbert's syndrome
    - Renal function:
        - serum creatinine ≤ 2.0mg/dL or Calculated Creatinine Clearance ≥ 25mL/min (Appendix 2)
11. Normal left ventricular ejection fraction (LVEF) by echocardiogram or MUGA.
12. Negative serum pregnancy test (only in childbearing potential female)
13. Patients with reproductive potential needs consistent and correct use of adequate non-hormonal methods of birth control (barrier methods or intrauterine device)

14. Previous treatment with anthracyclines and/or taxanes in the neo-adjuvant, adjuvant or advanced setting
15. At least one line of treatment for metastatic disease

Refer to Appendix 4 for additional inclusion criteria for patients who participate in the PET sub-study.

6.4. Exclusion Criteria

Subjects meeting <u>any</u> of the following criteria must not be enrolled in the study:

1. Any unstable systemic disease including active infections, significant cardiovascular disease, as well as myocardial infarction within the previous year, any significant hepatic (current active hepatic or biliary disease [with exception of patients with Gilbert's syndrome, asymptomatic gallstones, liver metastases or stable chronic liver disease per investigator assessment]), renal or metabolic disease, metabolic dysfunction, physical examination finding, or clinical laboratory finding that contraindicates the use of study medication or that might affect the interpretation of the results or render the patient at high risk from treatment complications.
2. Pregnant or lactating women
3. Dementia, altered mental status, or any psychiatric condition that could prohibit the understanding or rendering of informed consent
4. Lack of physical integrity of the upper gastrointestinal (GI) tract, poor absorption of the GI tract or inability to take oral medication
5. Other co-existing malignancy or malignancies within the last 5 years with the exception of basal cell carcinoma or in-situ carcinoma
6. Concurrent anti-cancer therapies (chemo or hormonal therapy) other than study drug
7. Concurrent treatment with an investigational agent or participation in any investigational drug study within 4 weeks preceding treatment start
8. Concurrent radiotherapy to the only target lesion. Concurrent bisphosphonates are allowed as long as this therapy has started before patient receives study medication.
9. Previous treatment with anti HER-2 or anti-EGFR therapies
10. Protocol specified treatment regimens that would be inappropriate for the management of the subject (See also concomitant medication Section 7.9).

Refer to Appendix 4 for additional exclusion criteria for patients who participate in the PET substudy.

6.5. Withdrawal Criteria

Treatment with investigational product should be discontinued if a subject experiences disease progression, unacceptable toxicities (as described Dose Adjustment, Modifications for Toxicities, Section 7.3), become pregnant, in the judgment of the investigator continuation of the treatment regimen is inappropriate for the clinical management of the subject or withdrawal of consent. In subjects presented with symptoms and signs of suggestive pneumonitis, lapatinib treatment should be withheld until diagnosis is confirmed. Patients diagnosed with pneumonitis possibly related to lapatinib should be removed from study.

Subjects with an NCI CTC AE Grade 3 or 4 left ventricular systolic dysfunction or interstitial pneumonitis must be withdrawn from treatment.

The investigator should perform all the assessments as listed for the Early Withdrawal in Table 8. The reason for withdrawal from study treatment must be documented in CRF.

A follow up visit should be performed in 28 days after last dose administration.

Subjects who are prematurely discontinued from the study will be not replaced.

7. STUDY TREATMENTS

7.1. Investigational Product

Lapatinib ditosylate monohydrate tablets, 250 mg, are oval, biconvex, orange, filmcoated tablets that are embossed on one side with FG HLS. Tablets contain 410 mg of lapatinib Ditosylate Monohydrate, equivalent to 250 mg lapatinib free base per tablet. Refer to the Investigator’s Brochure (IB) for information regarding the physical and chemical properties of the drug substance and list of excipients.

7.2. Dosage and Administration

Lapatinib should be given orally on an empty stomach (either 1 hour before or 1 hour after meals) at the dose of 1500 mg per day until PD, unacceptable toxicity or death whichever occurs first.

Lapatinib therapy should NOT be taken with grapefruit, grapefruit juice and these should not be taken at anytime during the study,

If vomiting occurs after lapatinib intake, the patient should be instructed not to retake the dose. If vomiting persists, then the patient should contact the investigator.

7.3. Dose Adjustment, Delays and Dose Modifications

Subjects will be treated until disease progression or withdrawal from study due to unacceptable toxicity or consent withdrawal. Every 4-week period subjects will be evaluated for evidence of drug-related toxicity. Throughout the study, the following criteria will be used to evaluate toxicity in haematology, blood chemistry and any non-hematologic toxicity.

Treatment will be **delayed if**:

Non-Hematologic Toxicity	Any Grade 3 or 4 drug-related toxicity
Hematology	ANC is < 1.0 x 109/L<br>Platelet count is < 75.0 x 109/L
Chemistry	Unresolved grade 3 or 4 toxicity (except bilirubin)<br>See Section 7.3.2 for liver function test abnormalities
Serum Creatinine and Calculated Creatinine Clearance[1]	≥2.0 mg/dL<br>≤ 25 mL/min

1. Calculated by the Cockcroft and Gault Method

Treatment may be delayed up to 2 weeks, to allow for resolution of toxicity except in the event of NCI CTCAE Grade 3 and 4 Left Ventricular Cardiac Dysfunction or NCI CTCAE Grade 3 and 4 interstitial pneumonitis, ***events that require patient's withdrawal.*** The investigator must consult with the Study Medical Monitor prior to continuing therapy for any subject requiring a delay of more than 2 weeks for unresolved toxicity, but in general, such subject should be withdrawn from the study.

If treatment is delayed for reasons other than toxicity (i.e. unplanned travel or vacation, or lack of transportation to the site) and the subject has insufficient investigational product available, the subject should resume the usual dosing schedule once the drug supply has been made available. However, if the subject has been off therapy for more than 2 weeks, the Investigator must consult with the Study Medical Monitor prior to continuing therapy.

Dose reduction for drug-related toxicity is permitted, however the Study Medical Monitor must be consulted prior to implementing any change in dosing. Once the dose has been reduced, subjects should not be rechallenged to a higher dose level. If a NCI CTCAE Grade 3 and 4 drug-related event (other than Left Ventricular Cardiac Dysfunction or Interstitial Pneumonitis) has occurred, the investigator may discuss with the GSK Medical Monitor whether a reduction of dose is appropriate.

Lapatinib starting dose and dose modifications for unacceptable toxicity are listed in Table 1. For toxicity that is thought to be related to lapatinib, the daily dose of lapatinib will be decreased according to the schedule displayed in the Table 2. Lapatinib dose adjustments are to be made according to the greatest degree of toxicity. Toxicities will be graded using NCI Common Terminology Criteria for Adverse Events (CTCAE) version 3.0.

Table 1 Lapatinib Starting Dose and Dose Reduction Schedule

Starting dose	1500 mg/day	
First dose reduction	1250 mg/day	If no recovery after 2 weeks of holding drug, patients must be withdrawn from the study unless in the opinion of the investigator & GSK Medical Monitor, there is reason to believe that the patient is still experiencing clinical benefit

Patients requiring a second dose reduction should be taken off study unless in the opinion of the investigator & GSK Medical Monitor, there is reason to believe that the patient is still experiencing clinical benefit

Table 2 Dose Reduction Criteria and Guidelines for Management of lapatinib Associated Toxicity

Toxicity	Grade	Guideline for Management	Lapatinib Dose Modification[1]
Diarrhea	1	stop all lactose containing products, drink 8-10 large glasses of clear liquids a day, eat frequent small meals	None
	2	Loperamide (4 mg at first onset, followed by 2 mg every 2–4 hrs until diarrhea free for 12 hrs)	Consider a dose reduction of lapatinib (discuss with medical monitor)
	≥ 3(*despite optimal use of loperamide)*		Hold until recovery to ≤ grade 1, up to 21 days[1]. And then reduce 1 dose level.
Rash	1	No intervention	None
	2	Any of the following: minocycline[3], topical tetracycline or clindamycin, topical silver sulfadiazine, diphenhydramine, oral prednisone (short course)	None; If unacceptable to patient or medically concerning then hold until recovery to ≤ grade 1, up to 21 days[1]. Restart at same dose[2].
	≥ 3		Hold until recovery to ≤ grade 1, up to 21 days[1]. And then reduce 1 dose level.
Other Toxicity (excluding left ventricular dysfunction and pneumonitis, see below)	1	No Intervention	None
	2	Treatment as appropriate	None; If unacceptable to patient or medically concerning then hold until recovery to ≤ grade 1, up to 21 days[1]. Restart at same dose[2].
	2 prolonged or clinically significant and grade ≥ 3	Treatment as appropriate	Hold until recovery to < grade 1, up to 21 days and then reduce 1 dose level[1]

1. if no recovery after 2 weeks of holding drug, patients must be withdrawn from unless in the opinion of the investigator & GSK Medical Monitor, there is reason to believe that the patient is still experiencing clinical benefit.
2. if dose has been previously held for grade 2 toxicity and grade 2 symptoms recur, OR if the patient finds the symptoms unacceptable, hold dose until recovery to < grade 1 and then reduce dose one level
3. recommended dose: 200mg po bid (loading dose), followed by 100mg po bid for 7-10 days

7.3.1. Criteria for Evaluating Asymptomatic Cardiac events

If a subject who is receiving full dose of Lapatinib experiences a ≥20% decrease in LVEF relative to baseline AND the LVEF is below the institution's lower limit of normal (LLN), another evaluation of LVEF must be performed 2 weeks later while still receiving study drug.

Upon completion of the first repeat evaluation of LVEF, the procedures described in Table 3, Table 4, Table 5, and Table 6 must be followed:

Table 3 Criteria for Continuing Study Drug Following First Repeat Cardiac Evaluation Performed 2 Weeks Later While Still Receiving Study Drug

Institution's Range	LVEF Relative Change From Baseline	Action to be Taken
below LLN	≥20%	temporarily withdraw study drug<br>repeat cardiac evaluation in 2 wks and follow procedures in Table 4
below LLN	<20%	reduce to 5 tablets (equivalent to 1250mg QD active drug)<br>repeat cardiac evaluation in 2 wks & follow procedures in Table 5
WNL	≥20%	continue study drug<br>repeat cardiac evaluation in 2 wks and follow procedures in Table 6
WNL	<20%	continue study drug<br>continue cardiac evaluation every 2 months; refer to Time & Events Table 8.

Abbreviations: LLN = lower limit of normal; LVEF = left ventricular ejection fraction; WNL = within normal limits

Table 4 Criteria for Continuing Study Drug Following Second Repeat Cardiac Evaluation in Patients with LVEF below LLN and ≥20% Relative Change from Baseline

Institution's Range	LVEF Relative Change From Baseline	Action to be Taken
below LLN	≥20%	permanently withdraw study drug<br>repeat cardiac evaluation every 4 wks for at least 16 wks or until resolution
below LLN	<20%	permanently withdraw study drug<br>repeat cardiac evaluation every 4 wks for at least 16 wks or until resolution
WNL	≥20%	permanently withdraw study drug<br>repeat cardiac evaluation every 4 wks for at least 16 wks or until resolution
WNL	<20%	reduce to 5 tablets (equivalent to 1250mg QD active drug)<br>continue cardiac evaluation every 3 months; refer to Time & Events Table 8.

Abbreviations: LLN = lower limit of normal; LVEF = left ventricular ejection fraction; WNL = within normal limits

Table 5 Criteria for Continuing Study Drug Following Second Repeat Cardiac Evaluation in Patients with LVEF below LLN and <20% Relative Change from Baseline

Institution's Range	LVEF Relative Change From Baseline	Action to be Taken
below LLN	≥20%	permanently withdraw study drug<br>repeat cardiac evaluation every 4 wks for at least 16 wks or until resolution
below LLN	<20%	permanently withdraw study drug<br>repeat cardiac evaluation every 4 wks for at least 16 wks or until resolution
WNL	≥20%	permanently withdraw study drug<br>repeat cardiac evaluation every 4 wks for at least 16 wks or until resolution
WNL	<20%	continue at reduced dose (1250mg OD)<br>continue cardiac evaluation every 2 months; refer to Time & Events Table 8

Abbreviations: LLN = lower limit of normal; LVEF = left ventricular ejection fraction; WNL = within normal limits

Table 6 Criteria for Continuing Study Drug Following Second Repeat Cardiac Evaluation in Patients with LVEF WNL and ≥20% Relative Change from Baseline

Institution's Range	LVEF Relative Change From Baseline	Action to be Taken
below LLN	≥20%	permanently withdraw study drug<br>repeat cardiac evaluation every 4 wks for at least 16 wks or until resolution
below LLN	<20%	permanently withdraw study drug<br>repeat cardiac evaluation every 4 wks for at least 16 wks or until resolution
WNL	≥20%	Reduce to 5 tablets (equivalent to 1250mg OD active drug)<br>continue cardiac evaluation every 2 months; refer to Time & Events Table 8.
WNL	<20%	continue study drug<br>continue cardiac evaluation every 3 months; refer to Time & Events Table 8.

Abbreviations: LLN = lower limit of normal; LVEF = left ventricular ejection fraction; WNL = within normal limits

NOTE: If **following a dose reduction**, a subject experiences a ≥20% decrease in LVEF relative to baseline **OR** the LVEF is below the institution's LLN, the subject will be permanently withdrawn from study drug and cardiac evaluations must be performed every 4 weeks for at least 16 weeks or until resolution.

A ≥ 20% relative decrease from baseline in LVEF (asymptomatic or symptomatic), that is below the institution's LLN is considered an SAE and must be reported to GSK (refer to Section 9 for definition of an SAE).

7.3.2. Liver Chemistry Stopping and Follow Up Criteria

7.3.2.1. Liver Chemistry Stopping Criteria

Liver chemistry stopping and follow up criteria have been designed to assure subject safety and evaluate liver event etiology. All subjects who meet liver chemistry criteria requiring permanent discontinuation of investigational product must continue to be followed for the study assessments and procedures as defined in Section 8. Study Assessments and Procedures and at the time points indicated in the Time & Events Table 8 in Section 8. Study Assessments and Procedures.

If a subject experiences ALT >3 × ULN and total bilirubin >2.0 × ULN (>35% direct; bilirubin fractionation required*), then the following actions must be taken:

- immediately and permanently discontinue investigational product;
- complete the SAE data collection tool, the liver event CRF, and the liver imaging and/or liver biopsy CRFs, if these tests are performed;
- in addition to the liver event follow up assessments defined in Section 7.3.2.2 Liver Chemistry Follow-up Criteria below, the following are suggested: specialist or hepatology consultation; anti-nuclear antibody, anti-smooth muscle antibody, and Type 1 anti-liver kidney microsomal antibodies; and liver imaging and/or liver biopsy to evaluate liver disease;
- promptly report the event to GSK within 24 hours of learning its occurrence (refer to Section 9.7.3. Prompt Reporting of Serious Adverse Events and other Events to GSK for guidance on prompt reporting to GSK);
- monitor every week until liver chemistries resolve, stabilize or return to within baseline values;
- do not re-challenge with investigational product.

***NOTE**: bilirubin fractionation should be performed if testing is available. If testing is unavailable and a subject meets the criterion of total bilirubin >2.0 × ULN, then the aforementioned actions must still be performed.

If a subject experiences:

- ALT >8 × ULN or
- ALT >5 × ULN persisting for ≥2 weeks : retest within 3 days from the first occurrence and then weekly to determine if ALT elevation persists or
- ALT >3 × ULN with signs or symptoms of hepatitis or hypersensitivity (the appearance or worsening of fatigue, nausea, vomiting, right upper quadrant pain or tenderness, fever, rash, or eosinophilia),

then hold investigational product for 2 weeks, repeat liver chemistry testing in 2 weeks, and then call the Medical Monitor to discuss the possibility of re-challenging with investigational product.

If the treatment is exhibiting efficacy **and** the subject wants to continue for potential benefit of lapatinib therapy after being informed of the results of liver chemistry testing, then the investigational product may be re-started at the reduced dose agreed upon by the investigator and the Medical Monitor. The liver event CRF should be completed and liver chemistries and aforementioned signs and symptoms should be monitored at a minimum of every 2 weeks until resolution, stabilization, or a return to baseline values, at which point monitoring should be continued per protocol.

If a subject experiences ALT >3 × ULN **but** <5 × ULN **and** total bilirubin ≤2 × ULN, without signs or symptoms of hepatitis or hypersensitivity, **and** who can be monitored weekly, then the following actions should be taken:

- continue investigational product;
- monitor weekly until liver chemistries resolve, stabilize, or return to within baseline, then monitor liver chemistries as per protocol assessment schedule;
  - if ALT >3 and < 5 × ULN for > 4 weeks , discontinue the treatment;
- if at any time this subject meets any of the aforementioned liver chemistry stopping criteria, then proceed as described above.

7.3.2.2. Liver Chemistry Follow up Criteria

For all subjects who meet any of the liver chemistry criteria described above, make every attempt to carry out the liver event follow up assessments described below:

- Viral hepatitis serology including:
  - Hepatitis A IgM antibody;
  - Hepatitis B surface antigen and Hepatitis B Core Antibody (IgM);
  - Hepatitis C RNA;
  - Cytomegalovirus IgM antibody;
  - Epstein-Barr viral capsid antigen IgM antibody (or if unavailable, obtain heterophile antibody or monospot testing);
  - Hepatitis E IgM antibody (if subject resides or has travelled outside USA or Canada in past 3 months);
- Serum creatine phosphokinase (CPK) and lactate dehydrogenase (LDH);
- Complete blood count with differential to assess eosinophilia;
- Record the appearance or worsening of clinical symptoms of hepatitis, or hypersensitivity, fatigue, decreased appetite, nausea, vomiting, abdominal pain, jaundice, fever, or rash as relevant on the AE report form;

- Record use of concomitant medications, acetaminophen, herbal remedies, other over the counter medications, or putative hepatotoxins, on the concomitant medications report form;
- Record alcohol use on the liver event alcohol intake case report form.

Refer to Section 15.5, Appendix 5 for a liver safety algorithm detailing stopping and follow up criteria.

A blood sample for PK analysis (approx. 3ml- see Study Manual) should be obtained for measurement of lapatinib concentration as soon as possible after the event is reported. The date, time, and amount of the last dose ingested by the subject, and the date and time at which the blood sample is obtained, should be recorded. Please see the Study Manual for PK sample instructions and collection details.

7.4. Packaging and Labeling

The contents of the label will be in accordance with all applicable regulatory requirements.

7.5. Handling and Storage

Lapatinib will be dispatched to a site only after receipt of required documents in accordance with applicable regulatory requirements and GSK procedures.

Investigational product must be stored in a secure area under the appropriate physical conditions for the product. Access to and administration of the investigational product will be limited to the investigator and designee. Lapatinib must be kept at room temperature (15-30°C) and a temperature log must be filled.

Investigational product must be dispensed or administered only to subjects enrolled in the study and in accordance with the protocol.

A Material Safety Data Sheet (MSDS) describing the occupational hazards and recommended handling precautions will be provided to site staff if required by local laws or will otherwise be available from GSK upon request.

7.6. Treatment Assignment

All subjects will receive open-label Lapatinib with 1500 mg QD dosing.

7.7. Product Accountability

In accordance with local regulatory requirements, the investigator, designated site staff, or head of the medical institution (where applicable) must document the amount of GSK investigational product dispensed and/or administered to study subjects, the amount returned by study subjects, and the amount received from and returned to GSK, when

applicable. Product accountability records must be maintained throughout the course of the study.

7.8. Treatment Compliance

A record of the number of tablets dispensed to and taken by each subject at each visit must be maintained and reconciled with the study medication and compliance records in the CRF.

At each site visit, the cause of any missed doses should be discussed. Any AE(s) associated with missed doses must be recorded in the CRF. Subjects should be instructed for the importance of compliance to study treatments.

7.9. Concomitant Medications and Non-Drug Therapies

7.9.1. Permitted Medications and Non-Drug Therapies

All concomitant medications taken during the study will be recorded in the CRF. The minimum requirement is that drug name and dates of administration are to be recorded. In addition, indication and dose information should also be captured on the CRF, if possible. If there are any questions on medications not listed below, please contact the medical monitor of GSK for further information and clarification.

The following will be recorded on the appropriate CRF pages:

- A complete list of prescription and over-the-counter medications that have been taken within 4 weeks prior to the first dose of study drug.
- All concomitant medications taken while subjects are receiving study drug.

Subjects should receive full supportive care during the study, including transfusion of blood and blood products, and treatment with antibiotics, antiemetics, antidiarrheals, and analgesics, as appropriate. Refer to Section 9.5 for supportive care guidelines for diarrhea.

Concomitant treatments with bisphosphonates and/or radiotherapy are allowed for non target bone lesion(s) prior to enrollment.

7.9.2. Prohibited Medications and Non-Drug Therapies

Lapatinib is a substrate for CYP3A4. Inducers and inhibitors of CYP3A4 may alter the metabolism of Lapatinib. Co-administration of lapatinib with weak or moderate inhibitors or inducers of CYP3A4 must be at the discretion of the prescribing physician and based on a risk/benefit assessment. The list of CYP3A4 inducers and inhibitors are prohibited from screening through discontinuation from study. Additionally, there may be potential interaction between Lapatinib and warfarin. Patients have experienced elevated INRs and bleeding with warfarin and quinazolines. Patients on warfarin and Lapatinib should have more frequent INR/PT determinations after starting Lapatinib (e.g. weekly

for the first month and weekly for a minimum of 2 weeks following discontinuation of Lapatinib).

All Herbal (Alternative) medicines are prohibited as well as medications that modify gastric pH.

Concurrent surgery and radiotherapy on target lesion (unless initiated as adjuvant therapy for treatment of the initial diagnosis of invasive breast cancer) is prohibited during the study treatment.

The list of prohibited medications from screening through discontinuation from study is in Table 7.

Table 7 Prohibited Medications

Drug Class	Agent	Wash-out[1]
CYP3A4 Inducers		
Antibiotics	all rifamycin class agents (e.g., rifampicin, rifabutin, rifapentine)	14 days
Anticonvulsants	phenytoin, carbamazepine, barbiturates (e.g., phenobarbital)	
Antiretroviral	efavirenz, nevirapine	
Glucocorticoids (oral)	cortisone (>50 mg), hydrocortisone (>40 mg), prednisone (>10 mg), methylprednisolone (>8 mg), dexamethasone (>1.5 mg)[2]	
Other	St. John's Wort, modafinil	
CYP3A4 Inhibitors		
Antibiotics	clarithromycin, erythromycin, troleandomycin	7 days
Antifungal	itraconazole, ketoconazole, fluconazole (>150 mg daily), voriconazole	
Antiretroviral, Protease Inhibitors	delaviridine, nelfinavir, amprenavir, ritonavir, indinavir, saquinavir, lopinivir	
Calcium channel blockers	verapamil, diltiazem	
Antidepressants	nefazodone, fluvoxamine	
GI Agents	cimetidine, aprepitant	
Other	grapefruit, grapefruit juice	
	amiodarone	6 months
Miscellaneous		
Antacids	Mylanta, Maalox, Tums, Rennies	1 hour before and after dosing
Herbal supplements[3]	Ginkgo biloba, kava, grape seed, valerian, ginseng, echinacea, evening primrose oil.	14 days

1. At the time of screening, if a patient is receiving any of the above listed medications/substances, the medication or substance must be discontinued (if clinically appropriate) for the period of time specified prior to administration of the first dose of study drug and throughout the study period in order for the patient to be eligible to participate in the study.
2. Glucocorticoid daily doses (oral) $\leq$ 1.5 mg dexamethasone (or equivalent) are allowed. Glucocorticoid conversions are provided in parentheses.
3. This list is not all-inclusive; therefore, for herbal supplements not listed, please contact the GSK Medical Monitor or Clinical Scientist.

NOTE: if future changes are made to the list of prohibited medications, a formal documentation will be created and stored with the study file. Any changes will be communicated to the investigative sites in the form of a letter.

7.10. Treatment after the End of the Study

When the subject is withdrawn from all investigational products, the subject will be treated as determined by the attending physician.

7.11. Treatment of Investigational Product Overdose

Subjects with suspected overdose should be monitored until investigational product can no longer be detected systemically (at least 5 half-lives). Follow-up physical examination with laboratory testing should be performed between 10 and 14 days after drug concentrations are undetectable and before the subject is discharged from the investigator's care. Any AEs or SAEs that occur as a result of an overdose should be reported to the GSK Medical Monitor.

8. STUDY ASSESSMENTS AND PROCEDURES

The study consists of three periods: screening, treatment and follow-up.

Table 8 Time and Events Table

Procedures	Screen/ Run-in	Treatment 4 week cycles			End of Therapy/Early Withdraw	Follow-up	Post Treatment (can be phone contacts)
	Up to 28 days prior 1st dose	Every cycle (4 weeks) on day 1 (-2/+1)	Every 2 cycles (8 weeks) (-2/+1)	Every 3 cycles (12 weeks) (-2/+1)		28 days after last drug admin.	Every 12 weeks
Written Informed Consent	X						
Subject Demography	X						
Medical History	X						
Disease History	X						
Therapy History	X						
Inclusion/Exclusion Criteria	X						
Evidence of HER-2 or EGFR positive CTCs	X						
HER-2 negative primary tumour[1]	X						
Clinical Tumour Assessment	X			x	x		
CAP CT or MRI scan[2]	X			x	x		
Brain CT or MRI scan[3]	(x)						
Bone scan	X			(x)	(x)		
Bone X-ray or CT[4]	(x)			(x)	(x)		
Survival Follow Up							x
PET substudy[5]	X	x					
Safety Assessments							
Concomitant Medication	X	x			x	x	
Physical Examination	X	x			x	x	x
Vital Signs	X	x			x	x	x
ECOG PS	X	x			x	x	x
12-lead ECG	X		x		x		
ECHO or MUGA	X		x		x		
Toxicity evaluation		x				x	

Continued

Table 8 Time and Events Table (Continued)

Procedures	Screen/ Run-in	Treatment 4 week cycles			End of Therapy/Early Withdraw	Follow-up	Post Treatment (can be phone contacts)
	Up to 28 days prior 1st dose	Every cycle (4 weeks) on day 1 (-2/+1)	Every 2 cycles (8 weeks) (-2/+1)	Every 3 cycles (12 weeks) (-2/+1)		28 days after last drug admin.	Every 12 weeks
Adverse Events[6]		x					
SAE[6]		x					
Hematology	X	x			x	x	
Chemistry	X	x			x	x	
Pregnancy Test [7]	S	As clinically indicated					
Investigational product							
Dispensing		x					
Assess compliance		x			x		

1. According to the local lab. It will be centrally re-assessed after patient registration
2. Allowable window for tumour imaging is ± 7 days
3. To be performed only if CSN or meningeal involvement are suspected
4. To be performed only if bone metastases are clinically indicated
5. PET substudy will be conducted in selected patients in the UK centre only.
6. AE and SAE should be assessed on Day one of every cycle and unscheduled visit.
7. Serum pregnancy test: serum within 14 day from 1st dose of Lapatinib

8.1. Critical Screening Assessments

All patients eligible for screening must sign an informed consent for the study before any study-related procedures are performed.

The screening period can least up to 28 days before first study drug administration.

The required observations are:

- Demography: date of birth, race,
- Medical an Therapy history
- Concomitant Medications
- Disease History: details of malignancy date of diagnosis, primary tumour histology, cancer stage, HER -2, EGFR and hormonal receptors status on primary tumours, previous cancer treatment for adjuvant and metastatic setting, ongoing toxicities related to previous treatment,

- Vital Signs: blood pressure, pulse rate, body temperature, body weight and height
- Physical Examination
- ECOG: it is recommended, wherever possible, that a patient's performance status will be assessed by the same person throughout the study.
- 12 lead ECG
- ECHO or MUGA
- Hematology, Chemestry (ANC, PLT, Hb, Total bilirubin, serum creatinine or Calculated Creatinine, ALT (SGPT), AST (SGOT), LDH, alkaline phosphatase)
- For all childbearing potential female a negative serum pregnancy test must be obtained within 14 days before starting Lapatinib treatment
- For the Italian sites (stratum 1) - Evaluation of HER-2 status on CTCs: 2 blood samples will be sent to the central laboratory (Prato hospital) for CTC evaluation.
- For the UK sites (stratum 2) - Evaluation of EGFR status on CTCs: 2 blood samples will be sent to the central laboratory (Hammersmith hospital) for CTC evaluation

For patients resulting CTC HER-2 + or EGFR+, a Paraffin-embedded tumour block or at least six unstained 4 μ sections of primary tumour will be collected and sent to the central laboratory. Italian samples should be sent to Prato hospital and UK samples should be sent to Hammersmith hospital.

NOTE: All laboratory assessments will be performed locally. Lab normal ranges must be provided to GSK before study start. For the purposes of this study, examinations or laboratory analyses that must have results provided by the day of the scheduled visit and recorded in CRF.

8.1.1. Procedures for blood and tumour tissue samples shipment to the central laboratory

All blood and tumour tissue samples must be clearly labelled with the patient number, protocol number, site number, and collection date. Details for the preparation and shipment of samples will be provided before the beginning of the study in a specific procedure manual.

Investigators should select patients whose primary tumour has been locally defined as HER-2 negative (i.e. no gene amplification or IHC 0/1+ or IHC 2+ and no amplification). Central confirmation of the HER-2 negative status will be required after study registration. Accordingly, during screening the investigator should verify that a paraffin-embedded tumour block or at least six unstained 4 μ sections of the primary tumour will be available for shipment at the central lab (Hospital of Prato, Oncology Unit for Italian sites and Hammersmith Hospital for the UK sites).

At the respective central lab, the HER-2 status on the primary tumour will be evaluated by FISH using standard criteria and manufacturer instructions for PathVysion Kit (Abbott Laboratories).

HER-2 and EGFR evaluation on CTC will be performed by the means of the CellSearch equipment (Immunicon, Huntingdon Valley, PA, USA). The CellSearch Circulating Tumour Cell Kit is intended for the enumeration of circulating tumour cells (CTC) of epithelial origin (CD45-, EpCAM+, and cytokeratins 8, 18+, and/or 19+) in whole blood.

The CellSearch HER-2 Tumour Phenotyping Reagent and the CellSearch EGFRTumour Phenotyping Reagent are used for identify those CTCs over-expressing HER-2 and EGFR respectively. For detail on methods refers to Appendix 3.

Flow-chart for blood and tumour tissue samples shipment to the central laboratory

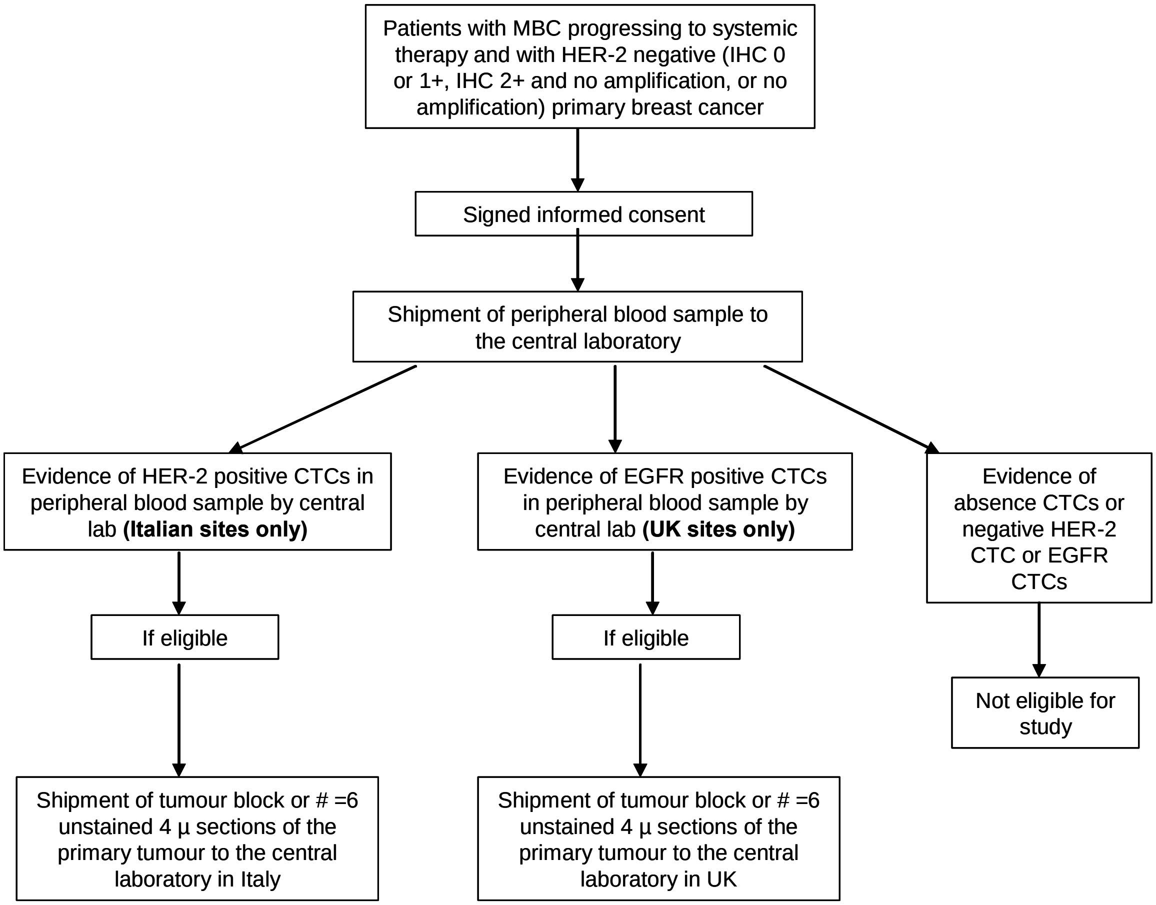

Two blood samples (10 +10 ml) will be shipped to the respective central laboratory for CTC evaluation. If the analysis is positive for presence of HER-2 over-expressing CTCs, *HER2* gene amplification will be evaluated by FISH. The patient will be eligible if the following criteria are met:

- For HER-2 positive CTCs
  - ≥ 50% CTCs are HER-2 positive and FISH in CTCs is positive or inconclusive
  - < 50% CTCs are HER-2 positive and FISH in CTCs is positive
- For EGFR positive CTCs
  - Presence of EGFR positive CTCs

8.2. Clinical Visit Schedules and Assessment during Study treatment and Follow up Periods

The Study Treatment Period is from start of the first dose of lapatinib on Day 1 until discontinuation of study treatment. Refer to the Time and Events Table 8 for the study assessments that need to be performed at each visit during the Study Treatment Period.

Follow up will commence from the time of stopping study drug. It consists of Follow up, 28 days after last dose of lapatinib and Survival follow up every 12 weeks after Follow up (can be assessed by phone contact if the patient cannot attend the visit). Refer to the Time and Events Table 8 for the study assessments that need to be performed.

8.3. Efficacy

8.3.1. Methods, Scope and Schedules of Disease Assessment

The following methods are acceptable for disease assessments in this study. The disease assessment scope and schedules are to be performed as clinically indicated.

CT and MRI: Conventional CT and MRI should be performed with contiguous cuts of 10mm or less in slice thickness. Spiral CT should be performed using a 5mm contiguous reconstruction algorithm; this specification applies to the tumours of the chest, abdomen, and pelvis. Tumours of head and neck, and extremities usually require specific protocols

NOTE: **x-ray, ultra sound, etc. are NOT PERMITTED by the protocol.**

Brain CT or MRI: are required only if CNS or meningeal involvement are suspected, to rule out brain or meningeal metastasis.

Bone Scan: Bone scan must be performed at screening (including evaluation of skull, total spine, clavicle, ribs, pelvis, and long bones). The exam must be repeated every 12 weeks (± 2 weeks) until progression if bone metastases have been diagnosed.

If a subject's bone-scan lesions are consistent with tumour metastasis, or are consistent with non-malignant lesions, confirmatory imaging assessments are not necessary. The anatomic sites and nature of the lesions must be documented in the CRF.

If bone scan lesions are equivocal for tumour metastasis, a confirmatory imaging assessment must be performed using X-ray, CT or MRI to determine the nature of the lesion. The lesion sites and nature of lesions must be documented in the CRF. For lesions that are confirmed as tumour metastasis, the same imaging modality must be used for all the subsequent follow-up bone lesion assessment.

Total tumour burden must be assessed within 4 weeks before starting first dose of lapatinib treatment. Follow-up tumour evaluations will be performed at 12 weekly intervals, unless for the disease progression is suspected, to confirm a partial or complete response. Allowable window for tumour imaging studies is ± 7 days.

If a patient inadvertently misses a prescribed tumour evaluation or a technical error prevents the evaluation, the patient may continue treatment until the next scheduled assessment, unless signs of clinical progression are present.

In cases where there is suspicion of progression before the next scheduled assessment, an unscheduled assessment is to be performed

8.3.1.1. Measurability of Tumour Lesions at Baseline

All measurements should be recorded in metric notation, using a ruler or calipers. All identified tumour lesions are to be classified as measurable or non-measurable per RECIST criteria [Therasse, 2000].

Measurable lesions: lesions that can be accurately measured in at least one dimension with the longest diameter ≥ 20 mm using conventional techniques, or ≥ 10 mm with spiral CT scan.

Non-measurable lesions: all other lesions, including lesions too small to be considered measurable (longest diameter < 20 mm using conventional techniques or < 10 mm with spiral CT scan), and the following lesions and disease sites: bone lesions, ascites, pleural/pericardial effusions, lymphangitis cutis/pulmonis, and cystic lesions.

Measurable disease: subjects presenting with at least one measurable lesion at baseline are identified as having **measurable disease.**

8.3.1.2. Determination of Target Lesion

At baseline, all the lesions must also be categorized as "Target" or "non-Target" lesions using the following RECIST guideline.

Target lesions: all measurable lesions up to a maximum of five lesions per organ and 10 lesions in total, representative of all involved organs should be identified as ***target lesions,*** and measured and recorded at baseline.

Target lesions should be selected on the basis of their size (lesions with the longest diameter) and their suitability for accurate repeated measurements (either by imaging techniques or clinically). Lesions that are present within a previously irradiated area cannot be selected as target lesions.

Non-target lesions: all other lesions (or sites of disease) should be identified as non-target lesions, and should also be recorded at baseline. Measurements of these lesions are not required, but the presence or absence of each should be noted throughout subsequent assessments.

8.3.1.3. Response Evaluation of Measurable Disease

A sum of the longest diameter (LD) for all target lesions will be calculated and reported as the baseline sum LD. The baseline sum LD will be used as reference by which to characterize the overall tumour response. During Treatment Assessments, any new lesion(s) must be recorded in the CRF. If the lesion(s) noted at baseline is not evaluated at the subsequent Treatment Assessment, this will be noted as 'not done' (ND) in the CRF.

RECIST Response criteria for target and non-target lesion(s) are presented in Table 9.

Table 9 RECIST Response Criteria for Target and non-Target Lesions

Evaluation of Target Lesions	
Complete response (CR)	Disappearance of all target lesions
Partial response (PR)	At least a 30% decrease in the sum of the LD of target lesions, taking as a reference the baseline sum LD
Progressive disease (PD)	At least a 20% increase in the sum of the LD of target lesions, taking as a reference the smallest sum LD recorded since the treatment started or the appearance of one or more new lesions
Stable disease (SD)	Neither sufficient shrinkage to qualify for PR nor sufficient increase to qualify for PD, taking as reference the smallest sum LD since the treatment started
Not evaluable (NE)	Any subject who cannot be classified by one of the four preceding definitions
Evaluation of non-Target Lesions	
Complete response (CR)	The disappearance of all non-target lesions
Incomplete response/Stable Disease (SD)	The persistence of one or more non-target lesion(s)
Progressive disease (PD)	The appearance of one or more new lesions and/or unequivocal progression of existing non-target lesions

New bone scan lesions that are equivocal will be considered as new malignant lesions if a confirmatory assessment using X-ray, CT or MRI is not available, and the subject will be determined as having progressive disease.

If a bone assessment is missing at baseline, any new bone lesions identified after the first dose that is either consistent with or equivocal for tumour metastasis will be considered as new bone (malignant) lesions and the subject will be considered as having progressive disease.

The best overall response is the best response recorded from the start of the treatment until disease progression/recurrence (taking as reference for PD the smallest measurements recorded since the treatment started).

Table 10 shows the overall response for all possible combinations of tumour responses in target and non-target lesions, with or without the appearance of new lesions.

Table 10 Evaluation of Best Overall Response

Target Lesions	Non-Target Lesions	New Lesions	Overall Response
CR	CR	No	CR
CR	Incomplete response/SD	No	PR
PR	Non-PD	No	PR
SD	Non-PD	No	SD
PD	Any	Yes or No	PD
Any	PD	Yes or No	PD
Any	Any	Yes	PD

Subjects with a global deterioration of health status requiring discontinuation of treatment without objective evidence of disease progression at that time should be classified as having "symptomatic deterioration". Every effort should be made to document the objective progression even after discontinuation of treatment.

In some circumstances it may be difficult to distinguish residual disease from normal tissue. When the evaluation of CR depends on this determination, it is recommended that the residual lesion be investigated (fine needle aspirate/biopsy) to confirm the CR status.

The purpose of this phase II trial is to reject lapatinib from further study in this indication if it was insufficiently active, and to accept it for further study if it was active. “Activity” is defined here as tumour response according to the RECIST criteria

Activity will be assessed from the time of starting the study drug until PD or unacceptable toxicity or death whichever occurs first. This will be performed by tumour assessment as above described.

8.4. Safety

8.4.1. Physical Examination

Physical examination is assessed at Screening, on Day 1, every Cycle (4 weeks) on day 1 until discontinuation of study treatment. Physical examination includes body weight and evaluation of the subject's medical conditions. Any new or worsening of medical conditions from the screening condition should be recorded in the AE or SAE CRF.

8.4.2. Vital Signs

Vital signs, including height, weight, heart rate, temperature and blood pressure should be obtained at Screening, on Day 1, every Cycle (4 weeks) on day 1 until discontinuation of study treatment.

8.4.3. ECOG PS

ECOG PS is assessed at Screening, on Day 1, every Cycle (4 weeks) on day 1 until discontinuation of study treatment.

8.4.4. Clinical Laboratory Assessment

Hematology, Chemistry (ANC, PLT, Hb, Total bilirubin, serum creatinine or Calculated Creatinine, ALT (SGPT), AST (SGOT), LDH, alkaline phosphatase) should be obtained at Screening, on Day 1, every Cycle (4 weeks) on day 1 until discontinuation of study treatment or as clinically needed.

Bilirubin fractionation is recommended if total bilirubin >2 x ULN when testing is available.

See Section 7.3.2 for details on Liver Chemistry Stopping and Follow-up criteria.

8.4.5. Criteria for Evaluating Asymptomatic Cardiac events

Cardiac Events will be obtained for at Screening and on Day 1, every 2 Cycles (8 weeks) until discontinuation of study treatment or as clinically needed.

If a subject experiences a ≥20% decrease in LVEF relative to baseline AND the LVEF is below the institution's lower limit of normal (LLN), another evaluation of LVEF must be performed 2 weeks later while still receiving study drug.

Upon completion of the first repeat evaluation of LVEF, the procedures described in Section 7.3.1 must be followed.

8.4.6. Toxicity Evaluation

Toxicity Evaluation should be obtained at Screening, on Day 1, every Cycle (4 weeks) on day 1 until discontinuation of study treatment

8.4.7. 12 Lead Electrocardiogram and ECHO or MUGA

12-lead ECG will be obtained for at Screening and on Day 1, every 2 Cycles (8 weeks) until discontinuation of study treatment or as clinically needed. Prior to each ECG test, the subject should be at rest for approximately 10 minutes. The subject should be in the semi-recumbent or supine position; the same position must be used for all subsequent ECG tests.

ECHO or MUGA will be obtained for at Screening and on Day 1, every 2 Cycles (8 weeks) until discontinuation of study treatment or as clinically needed. The same method must be used throughout the study.

8.5. Positron Emission Tomography (PET) Sub-study

A subgroup of subjects enrolled into stratum 2 (UK study group only) will be asked to participate in a sub-study using PET to quantify the potential early response to lapatinib treatment.

Response criteria for PET study: PET will be used to quantify response by calculating the change in PET uptake parameters like SUV (Standardised Uptake Value), Ki (constant for net irreversible uptake of tracer) and FRT (Fractional retention time). This will help in identifying early responders to treatment. PET will help in measuring MAP kinase activity and proliferation, both of which have been shown to be down regulated by lapatinib via its upstream blockade of EGFR and HER-2 receptor tyrosine kinases. Further details are provided in Appendix 4.

8.6. Long term remission (related to Amendment 01 – applicable only in UK)

In the study, there is not a defined stop for the study treatment. In the event of long term remission, even unlikely, the following screening regimen is applied for these subjects:

Investigation	Timeframe	Number of procedures
PET scan	Screening then 2 weeks	2
Bone Scintogram	Screening then 3, 6, 12 and 18 months	5
MUGA scan	Screening	1
CAP CT	Screening then 3, 6, 9, 12 and 18 months	6
Bone X-Ray	Screening then 3, 6, 9, 12 and 18 months	6

In the event of long term remission, any imaging performed after 18 months will only be done if clinically indicated (i.e. under standard IR(ME)R practice it is only done if the results will be of benefit to the patient's clinical care).

9. ADVERSE EVENTS (AE) AND SERIOUS ADVERSE EVENTS (SAE)

The investigator or site staff will be responsible for detecting, documenting and reporting events that meet the definition of an AE or SAE.

9.1. Definition of an AE:

Any untoward medical occurrence in a patient or clinical investigation subject, temporally associated with the use of a medicinal product, whether or not considered related to the medicinal product.

Note: An AE can therefore be any unfavorable and unintended sign (including an abnormal laboratory finding), symptom, or disease (new or exacerbated) temporally associated with the use of a medicinal product. For marketed medicinal products, this also includes failure to produce expected benefits (i.e. lack of efficacy), abuse or misuse.

Examples of an AE **include**:

- Exacerbation of a chronic or intermittent pre-existing condition including either an increase in frequency and/or intensity of the condition.
- New conditions detected or diagnosed after investigational product administration even though it may have been present prior to the start of the study.
- Signs, symptoms, or the clinical sequelae of a suspected interaction.
- Signs, symptoms, or the clinical sequelae of a suspected overdose of either investigational product or a concomitant medication (overdose per se will not be reported as an AE/SAE).
- "Lack of efficacy" or "failure of expected pharmacological action" per se will not be reported as an AE or SAE. However, the signs and symptoms and/or clinical sequelae resulting from lack of efficacy will be reported if they fulfill the definition of an AE or SAE.

Examples of an AE **does not** include a/an:

- Medical or surgical procedure (e.g. endoscopy, appendectomy); the condition that leads to the procedure is an AE.
- Situations where an untoward medical occurrence did not occur (social and/or convenience admission to a hospital).
- Anticipated day-to-day fluctuations of pre-existing disease(s) or condition(s) present or detected at the start of the study that do not worsen.
- The disease/disorder being studied, or expected progression, signs, or symptoms of the disease/disorder being studied, unless more severe than expected for the subject’s condition.

9.2. Definition of a SAE

A serious adverse event is any untoward medical occurrence that, at any dose:

a. Results in death

b. Is life-threatening

NOTE: The term 'life-threatening' in the definition of 'serious' refers to an event in which the subject was at risk of death at the time of the event. It does not refer to an event, which hypothetically might have caused death, if it were more severe.

c. Requires hospitalization or prolongation of existing hospitalization

NOTE: In general, hospitalization signifies that the subject has been detained (usually involving at least an overnight stay) at the hospital or emergency ward for observation and/or treatment that would not have been appropriate in the physician's office or out-patient setting. Complications that occur during hospitalization are AEs. If a complication prolongs hospitalization or fulfills any other serious criteria, the event is serious. When in doubt as to whether "hospitalization" occurred or was necessary, the AE should be considered serious.

d. Hospitalization for elective treatment of a pre-existing condition that did not worsen from baseline is not considered an AE.

e. Results in disability/incapacity,

NOTE: The term disability means a substantial disruption of a person's ability to conduct normal life functions. This definition is not intended to include experiences of relatively minor medical significance such as uncomplicated headache, nausea, vomiting, diarrhea, influenza, and accidental trauma (e.g. sprained ankle) which may interfere or prevent everyday life functions but do not constitute a substantial disruption.

f. Is a congenital anomaly/birth defect

g. Medical or scientific judgment should be exercised in deciding whether reporting is appropriate in other situations, such as important medical events that may not be immediately life-threatening or result in death or hospitalization but may jeopardize the subject or may require medical or surgical intervention to prevent one of the other outcomes listed in the above definition. These should also be considered serious. Examples of such events are invasive or malignant cancers, intensive treatment in an emergency room or at home for allergic bronchospasm, blood dyscrasias or convulsions that do not result in hospitalization, or development of drug dependency or drug abuse.

Any abnormal laboratory test results (haematology, clinical chemistry, or urinalysis) or other safety assessments (e.g., ECGs, radiological scans, vital signs measurements), including those that worsen from baseline, and felt to be clinically significant in the medical and scientific judgement of the investigator are to be recorded as AEs or SAEs.

However, any clinically significant safety assessments that are associated with the underlying disease, unless judged by the investigator to be more severe than expected for the subject's condition, are **not** to be reported as AEs or SAEs.

Additional protocol defined criteria:

- All grade 4 laboratory abnormalities
- Any signs or symptoms of pneumonitis that are ≥ Grade 3 (NCI CTCAE) (defined as radiographic changes and requiring oxygen). Refer to NCI CTCAE grading of pneumonitis/pulmonary infiltrates.

Cardiovascular events have been seen in subjects taking other compounds that inhibit ErbB2 when used in combination with or following anthracyclines and interstitial pneumonitis has been reported in subjects taking compounds that inhibit ErbB1. As a precaution, the following will be reported as an SAE:

- Cardiac dysfunction will be reported as an SAE and will be defined as any signs or symptoms of deterioration in left ventricular ejection fraction (LVEF) that are Grade 3 or 4 (NCI CTCAE) or a ≥ 20% relative decrease in LVEF from baseline which is also below the institution's LLN. Refer to NCI CTCAE grading of left ventricular cardiac function.

As a precaution, the following will be reported as SAE:

- Cardiac dysfunction will be reported as an SAE and will be defined as any signs or symptoms of deterioration in LVEF that are Grade 3 (NCI CTCAE) or a 20% decrease in LVEF from baseline, and cardiac ejection fraction is below the institution's lower limit of normal. Refer to NCI CTCAE grading of left ventricular cardiac function.
- Any signs or symptoms of pneumonitis that are Grade 3 (NCI CTCAE) (defined as radiographic changes and requiring oxygen). Refer to NCI CTCAE grading of pneumonitis/pulmonary infiltrates.
- Hepatobiliary events have been seen in subjects taking lapatinib and other tyrosine kinase inhibitors. As a precaution, the following will be reported as an SAE:
  - ALT >3 × ULN and total bilirubin >2.0 × ULN (>35% direct; bilirubin fractionation required).

  NOTE: bilirubin fractionation should be performed if testing is available. If testing is unavailable and a subject meets the criterion of total bilirubin >2.0 × ULN, then the event should still be reported as an SAE.

Other hepatic events should be documented as an AE or an SAE as appropriate.

Treatment modification in case of cardiac dysfunction or interstitial pneumonitis

Subjects who have a ≥20% decrease in left ventricular cardiac ejection fraction relative to baseline, and the ejection fraction is below the institution's lower limit of normal, should have a repeat evaluation of ejection fraction 1-2 weeks later while still receiving Lapatinib. If the repeat ejection fraction evaluation confirms a ≥20% decrease in left ventricular cardiac ejection fraction, and the ejection fraction is below the institution's lower limit of normal, then treatment should be temporarily discontinued. If the left

ventricular ejection fraction recovers during the next 3 weeks, after consultation with GSK, the subject may be restarted on treatment. In such a case, Lapatinib dose-reduction is indicated. For such subjects, monitoring of left ventricular ejection fraction will then be performed 2 weeks and 4 weeks after re-challenge, and then every 4 weeks thereafter. If repeat ejection fraction evaluation still shows a decrease of ≥20% in left ventricular ejection fraction relative to baseline, and the value is below the institution's lower limit of normal, then the subject should be withdrawn from treatment.

Subjects with an NCI CTC AE Grade 3 or 4 left ventricular systolic dysfunction or interstitial pneumonitis must be withdrawn from treatment.

9.3. Disease-Related Events and/or Disease-Related Outcomes Not Qualifying as SAEs

An event which is part of the natural course of the disease under study (e.g., disease progression) does not need to be reported as an SAE. Progression of the subject's neoplasia will be recorded in the clinical assessments in the CRF. Death due to progressive disease is to be recorded on the 'Record of Death' CRF page and not as an SAE. However, if the progression of the underlying disease is greater than that which would normally be expected for the subject, or if the investigator considers that there was a causal relationship between treatment study drug or protocol design/procedures and the disease progression, then this must be reported as an SAE. Any new primary cancer must be reported as an SAE.

9.4. Lack of Efficacy

"Lack of efficacy" per se will not be reported as an AE. The signs and symptoms or clinical sequelae resulting from lack of efficacy will be reported if they fulfill the AE or SAE definition (including clarifications).

9.5. Expected side effects

Rash: Skin rash (usually grade 1-2) has been observed during the first several days of treatment with EGFR inhibitors and has been observed to diminish in severity after 4 weeks of treatment in many patients. In some patients, this rash appeared to be treatable with standard acne therapies, including topical and oral antibiotics used to treat acne. Anecdotal reports of improvement have occurred with several agents. In patients with severe rash, treatment may need to be discontinued or the dose reduced. Anecdotal reports of improvement have occurred with any of the following: minocycline, topical tetracycline, topical clindamycin, topical silver sulfadiazine, diphenhydramine, oral prednisone (short course).

Diarrhea: These broad general management principles are recommended to proactively try and avoid more serious complications by active management of diarrhoea syndrome. Guidelines such as these should never replace sound clinical judgement. Experience thus far suggests that when lapatinib is used as monotherapy, uncomplicated Grade 1 or 2

diarrhea is most prevalent. These general management principles do not address comprehensive management of more serious or protracted diarrhea syndromes.

Common clinical sense with the onset of uncomplicated Grade 1-2 diarrhea: stop all lactose containing products: drink 8-10 large glasses of clear liquids a day; eat frequent small meals; for Grade 2 diarrhea, consider a dose reduction of lapatinib (discuss with medical monitor); administer standard doses of loperamide: initial dose 4 mg followed by 2 mg every 4 hours or after every unformed stool. It is suggested to continue loperamide until the subject is free from diarrhea for 12 hours.

For Grade 3 or 4 diarrhea or Grade 1 or 2 with complicating features (severe cramping, severe nausea/vomiting, decreased performance status, fever, sepsis, Grade 3 or 4 neutropenia, frank bleeding, dehydration) use intravenous fluids as appropriate, consider hospital administration. Use prophylactic antibiotics as needed (example flouroquinolones) especially if diarrhea is persistent beyond 24 hours or there is a fever or Grade 3-4 neutropenia, hold both cytotoxic chemotherapy and lapatinib and discuss with medical monitor.

Nausea: Routine premedication for nausea is not necessary, but symptomatic patients should be treated with standard antinausea/antiemetic therapy as necessary.

If the patient vomits after taking the tablets, the dose is replaced only if the tablets can actually be seen and counted.

Cardiac function: please refer to Section 7.3.1

9.6. Time Period and Frequency of Detecting AEs and SAEs

AE/SAE will be detected and recorded from the time a subject consents to participate in the study until he or she has completed the study (including a 28-day follow-up period). All SAEs assessed as related to study participation (e.g., protocol-mandated procedures, invasive tests, or change in existing therapy) or related to a GSK concomitant medication, will be reported promptly to GSK.

The investigator will monitor all ongoing AEs/SAEs after discontinuation of study treatment until the AE/SAE is resolved or until the subject is lost to follow-up.

9.7. Pregnancy

A screening serum β-HCG pregnancy test is mandatory for all women of child-bearing potential and should be done within 2 weeks prior to the first dose of Lapatinib. Thereafter, the urine or serum pregnancy test only needs to be repeated if clinically indicated or as required by local regulation.

9.7.1. Time period for collecting pregnancy information

The time period for collecting pregnancy information is identical to the time period for collecting AEs, as stated in Section 9 "Time Period, and Frequency of Detecting AEs and SAEs", from first dose of study treatment to 28 days after last dose.

9.7.2. Action to be taken if pregnancy occurs

If a female subject becomes pregnant while participating in the study, the study treatment must be immediately terminated. The investigator will collect pregnancy information record on the appropriate form and submit it to GSK within 2 weeks of learning of a subject's pregnancy. The subject will also be followed to determine the outcome of the pregnancy. Information on the status of the mother and child will be forwarded to GSK. Generally, follow-up will be no longer than 6 to 8 weeks following the estimated delivery date. Any premature termination of the pregnancy will be reported.

While pregnancy itself is not considered to be an AE or SAE, any pregnancy complication or elective termination of a pregnancy for medical reasons will be recorded as an AE or SAE (See Section 9 AE/SAE, of the protocol for definitions and a description of follow-up).

A spontaneous abortion is always considered to be an SAE and will be reported as such. Furthermore, any SAE occurring as a result of a post-study pregnancy and is considered reasonably related to the investigational product by the investigator, will be reported to GSK as described in Section 9. While the investigator is not obligated to actively seek this information in former study participants, he or she may learn of an SAE through spontaneous reporting.

9.7.3. Prompt Reporting of Serious Adverse Events and Other Events to GSK

SAEs, pregnancies, and liver function abnormalities meeting pre-defined stopping criteria will be reported promptly to GSK as described in the following table once the investigator determines that the event meets the protocol definition for that event.

	Initial Reports		Follow-up Information on a Previous Report	
Type of Event	Time Frame	Documents	Time Frame	Documents
All SAEs	24 hours	"SAE" data collection tool	24 hours	Updated "SAE" data collection tool
Pregnancy	2 Weeks	Pregnancy Notification Form	2 Weeks	Pregnancy Follow up Form
Liver chemistry abnormalities:				
ALT >3 × ULN and bilirubin[a] >2 × ULN (35% direct)	24 hours	Liver Event and Liver Imaging and/or Biopsy CRFs, if applicable	24 hours	Updated Liver Event CRF

a. bilirubin fractionation should be performed if testing is available. If testing is unavailable and a subject meets the criterion of total bilirubin >2.0 × ULN, then the event should still be promptly reported as defined

The method of detecting, recording, evaluating and follow-up of AEs and SAEs plus procedures for completing and transmitting SA reports to GSK are provided in the study procedure manual. Procedures for post-study AEs/SAEs are provided in the study procedure manual.

9.7.4. Regulatory Reporting requirements for SAEs

GSK has a legal responsibility to notify, as appropriate, both the local regulatory authority and other regulatory agencies about the safety of a product under clinical investigation. Prompt notification of SAEs by the investigator to the appropriate project contact for SAE receipt is essential so that legal obligations and ethical responsibilities towards the safety of other subjects are met.

The investigator, or responsible person according to local requirements, will comply with the applicable local regulatory requirements related to the reporting of SAEs to regulatory authorities, and the Institutional Review Board (IRB)/Independent Ethics Committee (IEC).

10. DATA MANAGEMENT

Data Management will identify and implement the most effective data acquisition and management strategy for each clinical trial protocol and deliver datasets which support the protocol objectives.

For this study subject data will be collected using GSK defined case report forms and if necessary combined with data provided from other sources (eg diary data, laboratory data) in a validated data system.

Clinical data management will be performed in accordance with applicable GSK standards and data cleaning procedures with the objective of removing errors and inconsistencies in the data which would otherwise impact on the analysis and reporting

objectives, or the credibility of the Clinical Study Report. Adverse events and concomitant medications terms will be coded using MedDRA and GSKDrug, an internal validated medication dictionary.

Original CRFs will be retained by GSK, while the investigator will retain a copy. In all cases, subject initials will not be collected nor transmitted to GSK.

11. DATA ANALYSIS AND STATISTICAL CONSIDERATIONS

11.1. Hypotheses

The purpose of this phase II trial is to reject Lapatinib from further study in this indication if it was insufficiently active, and to accept it for further study if it was active. "Activity" is defined here as tumour response according to the RECIST criteria. "Indication" is defined here as advanced breast cancer with HER-2 non-amplified primary tumours and HER-2 positive circulating tumour cells (stratum 1) or advanced breast cancer patients with HER-2 non-amplified and EGFR amplified primary tumours and EGFR positive circulating tumour cells (stratum 2). Each stratum will be analyzed separately.

The study is designed in each stratum as a two-stage three-outcome phase II trial with the following assumptions [Sargent, 2001]:

- the inactivity cut-off is chosen equal to 5%, the activity cut-off equal to 20%. Hence the hypotheses of interest are H_0: r ≤ 5% against H_A: r ≥ 20%, where r is the response rate
- the type I error rate (α, probability of accepting an insufficiently active treatment, a false positive outcome) is set to 10%
- the type II error rate (β, probability of rejecting an active treatment, a false negative outcome) is set to 5%
- the probabilities of correctly rejecting an inactive treatment, or of correctly accepting an active treatment, are both set to at least 80%

11.2. Study Design Considerations

11.2.1. Sample Size Assumptions

Given no response is typically expected with an anti-HER 2 treatment in the target patient population. It is believed that 10% response rate with lapatinib monotherapy will therefore be of interest in this exploratory study. Objective response will be assessed centrally by independent reviewers. Under these assumptions, the design consists of the following decision rule in each stratum:

treat 16 eligible subjects, and

- if no response is observed, declare the drug insufficiently active

- if 4 or more responses are observed, declare the drug sufficiently active
- if 1 to 3 responses are observed, proceed to the second stage as follows:

treat up to 15 additional subjects (up to 31 subjects in total), and

- if up to 2 responses in total are observed, declare the drug insufficiently active
- if 4 or more responses in total are observed, declare the drug sufficiently active
- if 3 responses in total are observed, no final conclusion can be drawn from this study

Note that after accrual of 16 subjects, the trial can terminate as soon as 4 responses are observed.

11.2.2. Sample Size Sensitivity

Not applicable.

11.2.3. Sample Size Re-estimation

Sample size re-estimation is not planned for this study.

11.3. Data Analysis Considerations

11.3.1. Analysis Populations

Per Protocol Population

The per protocol population will be defined as all subject fulfilling major entry inclusion/exclusion criteria who received at least 75 % of study drug doses up to first tumour assessment at week 12, and have at least one efficacy measurement available on the primary outcome. Subjects who progress prior to the week 12 assessment will also be included in this analysis. This population will be used for the primary objective of the study and for all efficacy analyses and summaries.

Intent-to-treat population

The intent-to-treat population will be defined as all subjects entered into the study. This population will be used to assess the sensitivity of the efficacy and safety analyses to protocol violations.

Safety Population

The safety population will be defined as all subjects who received at least one dose of study drug. The safety population will be used for all demographic, background and adverse event summaries and analyses. Subjects who do not receive treatment according to assignment will be grouped according to treatment received.

11.3.2. Analysis Data Sets

Not applicable.

11.3.3. Treatment Comparisons

This is a single-arm study with all eligible subjects receiving lapatinib, so there will be no comparisons between treatments.

11.4. Interim Analysis

An interim analysis will be performed as soon as 16 eligible subjects can be evaluated for response. The study recruitment will continue beyond 16 subjects until tumour response can be evaluated on the first 16 subjects.

11.5. Key Elements of Analysis Plan

This trial will use standard methodology applicable to oncology phase II trials. Details will be provided in the Statistical Analysis Plan. The final analysis will take place once all subjects have completed the study.

Withdrawal

Subjects will be treated until disease progression or withdrawal from study treatment due to unacceptable toxicity, consent withdrawal or other reasons. All data up to the time of withdrawal will be included in the analyses.

Subjects who are withdrawn prematurely from study treatment, but who are not withdrawn from the study at the same time, will be included in all analyses, regardless of the duration of treatment.

Missing Data

As the duration of treatment for a given subject will be dependent on efficacy and toxicity of study treatment, the duration of follow-up will vary between subjects. Consequently, there will be no imputation for missing data. Details will be given in the Statistical Analysis Plan. "Sub-sections" may be included or omitted for certain types of analyses, as applicable to the study purpose. Describe the following for each endpoint supportive of primary objective(s) using the relevant "sub-headings", including any specific requirements indicated in the "sub-sections" below:

- Details of any data derivation/transformation/pooling (e.g., of study sites) to be applied, and any subgroups to be analyzed.
- Methods for handling data from subjects, who are withdrawn from the study, investigational product, or both. Procedures for analyses if study is prematurely discontinued.

- Methods for handling unused, missing, spurious, unscheduled, or repeated data. For example, if missing data are imputed using a LOCF procedure, specify the methodology to be used.
- Planned methods used to summarize or analyze the data, giving details of models fitted, hypotheses, significance levels for main effects and interactions, covariates and planned comparisons. Omit any details already addressed in previous sections.
- The way to handle any anticipated analysis problems and report deviations from the planned analysis.
- Justification of models and/or reference to methodology considered uncommon or novel.
- Any applicable assessment windows, clinical concern/threshold values, or categorizations to be applied.
- Statement of any procedures to be applied for multiple tests (e.g., multiple endpoints/time points).

11.5.1. Efficacy Analyses

Primary Analysis-Overall Response Rate

The primary indicator of drug efficacy is the overall response rate (ORR), defined as the percentage of subjects achieving either a confirmed complete or partial tumour response. This will be based on confirmed responses from the independent assessment of best overall response. Subjects with unknown or missing response will be treated as non-responders, i.e., they will be included in the denominator when calculating the percentage.

A secondary (sensitivity) analysis of the primary endpoint will calculate the ORR based on subjects with evaluable tumour response only, i.e., the denominator will only include subjects with CR, PR, SD or PD and will not include those subjects with unknown or missing best response. Further details will be provided in the Statistical Analysis Plan.

Secondary Analyses

The following secondary endpoints will be analysed. Tumour response and disease progression will be based on the assessments from the independent review of objective evidence (e.g., radiological scans and medical photographs).

- **Clinical benefit response rate**: the percentage of subjects achieving a confirmed or partial response or stable disease for ≥24 weeks.
- **Duration of response**: for the subset of subjects who show a confirmed or partial tumour response, the time from first documented evidence of CR or PR until the first documented sign of disease progression or death due to breast cancer.
- **Time to tumour progression**: the time from the start of treatment until the earliest date of disease progression or death due to breast cancer, if sooner.

- **Time to best response**: the time from the start of treatment until first documented evidence of complete or partial tumour response. When best tumour response is confirmed at a repeat assessment, the time to best response will be taken to be the time that the best response was observed.

Further details of the analysis of secondary endpoints will be addressed in the Statistical Analysis Plan.

11.5.2. Safety Analyses

The ITT population will be used for the analysis of safety data.

Extent of Exposure

The number of subjects administered lapatinib will be summarised according to the duration of treatment. The daily dose and cumulative dose of lapatinib will also be summarised.

Adverse events

Adverse events (AEs) will be coded using the standard GlaxoSmithKline Medical Dictionary for Regulatory Activities (MedDRA) dictionary, and grouped by system organ class. AE terms may also be aggregated according to the Aggregation Guidelines for AE Terms in Lapatinib Studies.

Events will be summarised by frequency and proportion of total subjects, by system organ class and preferred term. Separate summaries will be given for all AEs, drug-related AEs, serious AEs leading to discontinuation of investigational product.

If the AE is listed in the NCI CTCAE table, the maximum grade will be summarised.

The incidence of deaths will also be reported and the primary cause of death summarised.

Clinical Laboratory Evaluations

Haematology and clinical chemistry data will be summarised at each scheduled assessment according to NCI CTCAE toxicity grade. The proportion of values lying outside the reference range will also be presented.

Other Safety Measures

The results of scheduled assessment of body weight, vital sign, 12-lead ECG, echocardiogram (or MUGA scan) and performance status will be summarised. Further details will be provided in the Statistical Analysis Plan.

12. STUDY CONDUCT CONSIDERATIONS

Prior to initiation of a study site, GSK will obtain approval from the appropriate regulatory agency to conduct the study in accordance with applicable country-specific regulatory requirements.

The study will be conducted in accordance with Good Clinical Practice (GCP), all applicable subject privacy requirements, and the guiding principles of the declaration of Helsinki, including, but not limited to:

- Institutional Review Board (IRB)/Independent Ethics Committee (IEC) review and approval of study protocol and any subsequent amendments.
- Subject informed consent.
- Investigator reporting requirements.

GSK will provide full details of the above procedures, either verbally, in writing, or both.

Written informed consent must be obtained from each subject prior to participation in the study.

12.1. Quality Control (Study Monitoring)

In accordance with applicable regulations, GCP, and GSK procedures, GSK monitors will contact the site prior to the start of the study to review with the site staff the protocol, study requirements, and their responsibilities to satisfy regulatory, ethical, and GSK requirements. When reviewing data collection procedures, the discussion will include identification, agreement and documentation of data items for which the CRF will serve as the source document.

GSK will monitor the study to ensure that the:

- Data are authentic, accurate, and complete.
- Safety and rights of subjects are being protected.
- Study is conducted in accordance with the currently approved protocol and any other study agreements, GCP, and all applicable regulatory requirements.

The investigator and the head of the medical institution (where applicable) agrees to allow the monitor direct access to all relevant documents

12.2. Quality Assurance

To ensure compliance with GCP and all applicable regulatory requirements, GSK may conduct a quality assurance audit of the site records, and the regulatory agencies may conduct a regulatory inspection at any time during or after completion of the study. In the event of an audit or inspection, the investigator (and institution) must agree to grant the auditor(s) and inspector(s) direct access to all relevant documents and to allocate their time and the time of their staff to discuss any findings/relevant issues.

12.3. Study and Site Closure

Upon completion or termination of the study, the GSK monitor will conduct site closure activities with the investigator or site staff (as appropriate), in accordance with applicable regulations, GCP, and GSK Standard Operating Procedures.

GSK reserves the right to temporarily suspend or terminate the study at any time for reasons including (but not limited to) safety issues, ethical issues, or severe noncompliance. If GSK determines that such action is required, GSK will discuss the reasons for taking such action with the investigator or head of the medical institution (where applicable). When feasible, GSK will provide advance notice to the investigator or head of the medical institution of the impending action.

If a study is suspended or terminated for safety reasons, GSK will promptly inform all investigators, heads of the medical institutions (where applicable),and/or institutions conducting the study. GSK will also promptly inform the relevant regulatory authorities of the suspension/termination along with the reasons for such action. Where required by applicable regulations, the investigator or head of the medical institution must inform the IRB/IEC promptly and provide the reason(s) for the suspension/termination.

12.4. Records Retention

Following closure of the study, the investigator or head of the medical institution (where applicable) must maintain all site study records (except for those required by local regulations to be maintained elsewhere) in a safe and secure location. The records must be easily accessible when needed (e.g., for a GSK audit or regulatory inspection) and must be available for review in conjunction with assessment of the facility, supporting systems, and relevant site staff.

Where permitted by local laws/regulations or institutional policy, some or all of the records may be maintained in a format other than hard copy (e.g., microfiche, scanned, electronic); however, caution must be exercised before such action is taken. The investigator must ensure that all reproductions are legible and are a true and accurate copy of the original. In addition, they must meet accessibility and retrieval standards, including regeneration of a hard copy, if required. The investigator must also ensure that an acceptable back-up of the reproductions exists and that there is an acceptable quality control procedure in place for creating the reproductions.

GSK will inform the investigator of the time period for retaining the site records in order to comply with all applicable regulatory requirements. The minimum retention time will meet the strictest standard applicable to a particular site, as dictated by local laws/regulations, GSK standard operating procedures, and/or institutional requirements, otherwise, the retention period will default to 15 years.

The investigator must notify GSK of any changes in the archival arrangements, including, but not limited to archival of records at an off-site facility or transfer of ownership of the records in the event that the investigator is no longer associated with the site.

12.5. Provision of Study Results and Information to Investigators

Where required by applicable regulatory requirements, an investigator signatory will be identified for the approval of the clinical study report. The investigator will be provided reasonable access to statistical tables, figures, and relevant reports and will have the opportunity to review the complete study results at a GSK site or other mutually-agreeable location.

Upon completion of the clinical study report, GSK will provide the investigator with the full summary of the study results. The investigator is encouraged to share the summary results with the study subjects, as appropriate.

12.6. Provision of Study Results and Information to Investigator

Where required by applicable regulatory requirements, an investigator signatory will be identified for the approval of the clinical study report. The investigator will be provided reasonable access to statistical tables, figures, and relevant reports and will have the opportunity to review the complete study results at a GSK site or other mutually-agreeable location.

Upon completion of the clinical study report, GSK will provide the investigator with the full summary of the study results. The investigator is encouraged to share the summary results with the study subjects, as appropriate.

13. PHARMACOGENETICS RESEARCH

13.1. Pharmacogenetics – Background

Pharmacogenetics (PGx) is the study of variability in drug handling or response due to hereditary factors in different populations. There is increasing evidence that an individual's genetic composition (i.e. his or her genotype) may impact drug handling or response, or clinical outcome. Genetic markers may be used to help understand and predict drug handling or response in terms of efficacy and/or safety and tolerability. Some reported examples of PGx analysis include:

Drug	Disease	Gene	Outcome
6-mercaptopurine (6-MP)	lymphoblastic leukemia	S-methyltransferase	Deficiency of the TPMT enzyme can be associated with toxicity and severe myelosuppression as patients are not able to sufficiently clear active thioguanine nucleotides (1, 2).
5-FU	Colorectal cancer	Dihydropyrimidine dehydrogenase (DPD)	Variants in the DPD gene result in little or no DPD and predisposition to toxicity to 5-FU (3-5)
Atomoxetine<br>Desipramine	ADHD<br>Depression	CYP2D6	Polymorphism in CYP2D6 result in different phenotypes; poor, intermediate, or extensive metabolizers. Poor metabolizing genotypes at risk of drug accumulation and associated toxicity (8-9)

A key component to successful PGx research is the collection of samples during the conduct of clinical studies. Collection of whole blood samples, even when no *a priori* hypothesis has been identified, may enable PGx analysis to be conducted if at any time it appears that there is a potential unexpected or unexplained variation in handling or response to GW572016.

13.2. Scope of PGx Analysis

PGx analysis may be conducted if unexplained variation in response to or handling of GW572016 (e.g., pharmacokinetics, efficacy and/or safety) that may be attributable to genetic variation are observed. In these circumstances, the analysis undertaken will be limited to PGx analysis of GW572016 handling or response and may include the evaluation of specific candidate genes, the conduct of a whole genome single nucleotide polymorphism (SNP) scan or other marker scan. Polymorphisms in target ErbB1, ErbB2, and other relevant genes previously associated with the adverse event in question will be evaluated. For the whole genome SNP scan approach, SNP or other genetic marker sets across the genome may be evaluated to identify those markers associated with differential drug handling or response.

The need to conduct PGx analysis may be identified after a study (or set of studies) of GW572016 has been completed and the study data reviewed. For this reason, samples may be kept for up to 15 years after the last subject completes the study or GSK may destroy the samples before then. In special cases, the samples may not be studied. This might happen if there are not enough subjects, if the study is stopped for other reasons, or if no questions are raised about how people respond to or handle GW572016.

In the performance of the PGx analysis, GSK may use subjects' medical information, samples and/or research results. This PGx research is not designed to determine whether other members of the subject's family are at risk of developing breast cancer or their response to or handling of GW572016.

Sample Quality Control (QC)

If deoxyribonucleic acid (DNA) is extracted from blood samples taken from the clinical study, the DNA may be subjected to sample quality control (QC) analysis. This analysis will involve the genotyping of several genetic markers to confirm the integrity of individual samples. If inconsistencies are noted in the analysis, then those samples may be destroyed.

13.3. Eligibility Criteria Considerations for PGx Participation

Any subject, who has given informed consent to participate in the clinical study, has met all the criteria required for entry into the clinical study may take part in the PGx research. Any subject who has received a bone marrow transplant must be excluded from the PGx research.

Subject participation in the PGx research is voluntary and refusal to participate will not indicate withdrawal from the clinical study. Refusal to participate will involve no penalty or loss of benefits to which the subject would otherwise be entitled.

No administration of investigational product beyond that detailed in the clinical study is associated with the PGx research.

Employees of GSK enrolled in the clinical study will not be eligible for participation in disease-based diagnostic research.

13.4. Pharmacogenetics Samples

In subjects who consent to PGx research, a 10 mL whole blood sample will be collected for the PGx research using a tube containing EDTA. The PGx sample is labeled (or "coded") with a study specific number that can be traced or linked back to the subject by the investigator or site staff. Coded samples do not carry personal identifiers (such as name or social security number). The blood sample will be taken on a single occasion unless a duplicate sample is required due to inability to utilize the original sample. It is recommended that the blood sample be taken at the first available opportunity, but may be taken at any time while the subject is participating in the clinical study. Sample collection and shipping instructions are provided in the Quest Manual.

13.5. Confidentiality of Subject's PGx Data

GSK advises that participation in this PGx research, withdrawal from this research, sample destruction, and/or PGx results should not be documented in the subject's medical records. Storage of information regarding the PGx research with source documents for the study is permissible if stored in the investigator study files.

Coded PGx samples and results will be associated with the subject's study specific number in computer databases. Coded PGx research results may be submitted to regulatory agencies as part of an investigational product submission and/or included in a research publication.

Individual genotype results will only be shared with a subject through the investigator if the subject requests to see their results. The results are for research purposes only, the clinically significance of which could be undetermined as such an early stage of research and not immediately useful to the subject's health. GSK will not release individual PGx results to anyone else (e.g., family members, primary care physicians, insurers, or employers) under any circumstance, unless required by law.

13.6. Subject Withdrawal

If a subject who has consented to participate in PGx research withdraws from the clinical study for any reason other than lost to follow-up, the subject will be given the following options concerning the PGx sample if already collected:

- PGx research continues per the subject's consent; or
- Any remaining sample is destroyed.

If a subject withdraws consent from the PGx research or requests sample destruction, the investigator must complete the appropriate documentation to request sample destruction within the timeframe specified by GSK and maintain the documentation in the site study records. In either case, GSK will only keep and study information collected/generated up to that point.

13.7. PGx Data Management

Data from the case report forms and PGx research using the coded sample will be stored electronically. International regulations for information on computers and relevant laws on processing personal information will be followed.

13.8. Pharmacogenetics Analyses

If at any time it appears that there is a potential unexpected or unexplained variation in drug handling or response (e.g., pharmacokinetics, efficacy and/or safety) that may be attributable to genetic variation, then PGx analysis may be conducted. In these circumstances the analysis undertaken will be limited to PGx analysis of response to or handling of GW572016.

Generally GSK will utilize two approaches to explore genetic variation in drug handling or response.

1. Hypothesis driven approach: A specific hypothesis is generated about sections of DNA (or individual single nucleotide polymorphisms (SNPs) or other genetic markers) that may be associated with differential drug handling or response. Specific sections of DNA may be selected from areas of the genome (e.g., candidate genes) known to encode the drug target, drug metabolizing enzymes, areas associated with mechanisms underlying adverse events, and those linked to study disease and, thus, linked to drug response.
2. Genome-wide approach utilizing polymorphic markers (e.g., SNPs): By evaluating large numbers of polymorphic markers throughout the genome, sets of markers may be identified that correspond to differential drug response or handling.

Analysis of genetic markers (e.g., whether within candidate genes or SNPs studied in a genome-wide analysis) will include the following considerations. The genotypic frequencies of each polymorphism will be evaluated for conformity to those expected under normal conditions by employing Hardy-Weinberg Equilibrium testing. Any departure from expectation will be taken into account, possibly signaling a data error or alternatively a connection between the polymorphism and breast cancer.

For pairs of polymorphisms, the degree to which alleles from the two sites are correlated (linkage disequilibrium) may also be evaluated. If the genotypes at two polymorphic sites within a gene are shown to be statistically associated with a response to investigational product, the degree of linkage disequilibrium will aid interpretation in that it will indicate the extent to which the two sites are exerting independent effects.

A decision regarding the construction and analysis of marker haplotypes -- combinations of alleles from different polymorphic sites that are inherited from one parent -- may be guided by the assessment of linkage disequilibrium. For example, if there is no linkage disequilibrium between polymorphic sites, then haplotype construction will be uninformative.

Differences in baseline clinical characteristics and potential contributing covariates may be summarized and compared among genotype (or haplotype) subgroups.

Analyses may be carried out to evaluate the degree of association between subject genotype (or haplotype) and selected parameters (e.g., efficacy and safety). Where such genotypic tests are inappropriate (for example, where the number of marker genotypes is too large and/or the frequency of individual genotypes too small), allelic tests may be conducted. Allelic tests evaluate whether the frequency of each marker allele is the same in responders and non-responders.

In addition to evaluating the main effects of the genotypes (haplotypes or alleles) on the selected parameters, the possibility of a treatment group by genotype (haplotype or allele) interaction may also be explored. If appropriate, the joint effects of multiple markers (gene-gene interactions) may also be evaluated.

Sample Size Considerations

The ability to detect differential drug response or handling among genotypes at a polymorphic site depends on the total number of subjects genotyped and the frequency distribution of the different genotypes. Consequently, genotyping analyses are plausible for those polymorphic sites where the number of subjects comprising the genotypic groups is sufficiently large; however, these frequencies will not be known until sufficient samples have been collected and genotyping is complete.

Estimates of sample sizes required to demonstrate genotype effects vary considerably, depending on the assumptions made about allele frequency, genetic effect size, and mechanism of inheritance [Cardon, 2000]. In the work by Palmer and Cookson [Palmer, 2001], which assumed a genotype relative risk of 1.5, it was estimated that more than 300 cases and 600 controls would be needed to conduct a genetic association analysis. In contrast, McCarthy and Hilfiker [McCarthy, 2000] showed that with a

genotype relative risk of 2.16 and a relatively commonly occurring genotype, only 30 cases and 30 controls would be needed to demonstrate an association. Consequently, it is quite possible that effects with relatively large genotype relative risks may be detectable in individual Phase I or Phase II studies. A range of examples exist to demonstrate robust and clinically relevant PGx data may be generated from PGx studies with sample sizes far less than the sizes proposed by Palmer and Cookson.

Published PGx examples include abacavir hypersensitivity reaction [Hetherington, 2002; Mallal, 2002] and tranilast induced hyperbilirubinemia [Roses, 2002] where genetic markers have been found to significantly associate with hypersensitivity reaction (abacavir) and hyperbilirubinemia (tranilast). These examples show that small sample sizes typically encountered in Phase I and Phase II studies may be sufficient to identify clinically relevant genetic associations.

14. REFERENCES

Allred DC, Clarck GM, Tandon AK, et al. HER-2/neu in node-negative breast cancer: Prognostic significance of overexpression influenced by the presence of in situ carcinoma. J Clin Oncol 1992; 10:599-605

Beluz-Riche D, Robert J, Riche C et al. Effects of paclitaxel, cyclophosphamide, ifosfamide, tamoxifen and cyclosporine on the metabolism of methoxymorpholinodoxorubin in human liver microsomes. Cancer Chemother Pharmacol. 2002; 49: 274- 280

Biscardi JS, Ishizawar RC, Silva CM et al Tyrosine kinase signalling in breast cancer Epidermal growth factor receptor and c-Src interactions in breast cancer. Breast Cancer Res 2000; 2(3): 203-10

Blackwell KL, Kaplan EH, Franco SX et al. A phase II, open-label, multicenter study of GW572016 in patients with trastuzumab-refractory metastatic breast cancer. Proc. American Society of Clinical Oncology meeting 2005, abstr. no. 3006

Burgess AW, Cho HS, Eigenbrot C, Ferguson KM, Garrett TP, Leahy DJ, Lemmon MA, Sliwkowski MX, Ward CW, Yokoyama S. An open-and-shut case? Recent insights into the activation of EGF/ErbB receptors. Mol Cell. 2003 Sep;12(3):541-52.

Burnstein H, Storniolo AM, Franco S, et al. A phase II, open-label, multicenter study of lapatinib in two cohorts of patients with advanced or metastatic breast cancer who have progressed while receiving trastuzumab-containing regimens. Proc. 29th European Society of Medical Oncology Meeting 2005, abstr. no. 104

Cardon LR, Idury RM, Harris TJR, Witte JS, Elston RC. Testing drug response in the presence of genetic information: sampling issues for clinical trials. Pharmacogenetics. 2000; 10:503-10.

Carney WP, Neumann R, Lipton A, Leitzel K, Ali S, Price CP. Monitoring the circulating levels of the HER2/neu oncoprotein in breast cancer. Clin Breast Cancer 2004 Jun; 5(2): 105-16

Carney WP, Neumann R, Lipton A, Leitzel K, Ali S, Price CP. Potential clinical utility of serum HER-2/neu oncoprotein concentrations in patients with breast cancer. Clin Chem. 2003 Oct; 49(10): 1579-98

Cristofanilli M, Budd GT, Ellis MJ et al. Circulating Tumour Cells, Disease Progression, and Survival in Metastatic Breast Cancer. N Engl J Med 2004 Aug 19; 351(8): 781-91

Cristofanilli M, Hayes DF, Budd GT et al. Circulating Tumour Cells: A Novel Prognostic Factor for Newly Diagnosed Metastatic Breast Cancer. J Clin Oncol. 2005 Mar 1; 23(7): 1420-30

Geyer CE, Forster J, Lindquist D, et al. Lapatinib and capecitabine for HER2-Positive Advanced Breast Cancer. . N Engl J Med 2006; 355: 2733 - 43

Gomez HL, Chavez MA, Doval DC et al. Efficacy and Safety of Lapatinib As First-Line Therapy for ErbB2-Amplified Locally Advanced or Metastatic Breast Cancer. JCO 2008, vol 26, page 2996- 3005.

Graus-Porta D, Beerli RR, Daly JM, Hynes NE. ErbB-2, the preferred heterodimerization partner of all ErbB receptors, is a mediator of lateral signaling.
EMBO J. 1997 Apr 1;16(7):1647-55

Hanahan D, Weinberg RA. The Hallmarks of Cancer. Cell 2000; 103: 211-25

Hendriks BS, Opresko LK, Wiley HS, Lauffenburger D. Quantitative analysis of HER2-mediated effects on HER2 and epidermal growth factor receptor endocytosis: distribution of homo- and heterodimers depends on relative HER2 levels.J Biol Chem. 2003 Jun 27; 278(26):23343-51. Epub 2003 Apr 9.

Hetherington S, Hughes AR, Mosteller M, Shortino D, Baker KL, Spreen W, Lai E, Davies K, Handley A, Dow DJ, Fling ME, Stocum M, Bowman C, Thurmond LM, Roses AD. Genetic variations in HLA-B region and hypersensitivity reactions to abacavir. Lancet. 2002; 359:1121-2.

Klapper LN, Kirschbaum MH, Sela M et al. Biochemical and clinical implications of the ErbB/HER signaling network of growth factor receptors. Adv Cancer Res 2000; 77: 25-79

Lipton A, Leitzel K, Ali SM, Demers L, Harvey HA, Chaudri-Ross HA, Evans D, Lang R, Hackl W, Hamer P, Carney W. Serum HER-2/neu conversion to positive at the time of disease progression in patients with breast carcinoma on hormone therapy. Cancer 2005 Jul 15; 104(2): 257-63

Mallal S, Nolan D, Witt C, Masel G, Martin AM, Moore C, Sayer D, Castley A, Mamotte C, Maxwell D, James I. Association between presence of HLA-B*5701, HLA-DR7, and HLA-DQ3 and hypersensitivity to HIV-1 reverse-transcriptase inhibitor abacavir. Lancet. 2002; 359:727-32.

McCarthy JJ, Hilfiker R. The use of single-nucleotide polymorphism maps in pharmacogenomics. Nat Biotechnol. 2000; 18:505-8.

Meng S, Tripathy D, Shete S et al. HER-2 gene amplification can be acquired as breast cancer progresses. Proc Natl Acad Sci 2004 Jun 22; 101(25): 9393-8

Palmer LJ, Cookson WO. Using single nucleotide polymorphisms as a means to understanding the pathophysiology of asthma. Respir Res. 2001; 2:102-12.

Perez EA, Byrne JA, Hammond R, et al. Results of an analysis of cardiac function in 2,812 patients treated with lapatinib.(abs 583). 2006 ASCO Annual Meeting Proceedings 24[18S].

Roses AD. Genome-based pharmacogenetics and the pharmaceutical industry. Nat Rev Drug Discovery. 2002; 1:541-9.

Rusnak DW,Lackey K, Affleck K et al. The Effects of the Novel, Reversible Epidermal Growth Factor Receptor/ErbB-2 Tyrosine Kinase Inhibitor, GW572016, on the Growth of Human Normal and Tumour-derived Cell Lines in Vitro and in Vivo. Mol Cancer Ther 2001 Dec; 1(2): 85-94

Sargent DJ, Chan V, Goldberg RM. A three-outcome design for phase II clinical trials. *Controlled Clinical Trials* 2001;22:117-125

Shewchuk LM, Hassell AM, Ellis B, Holmes WD, Davis R, Horne EL, Kadwell SH, McKee DD, Moore JT. Structure of the Tie2 RTK domain: self-inhibition by the nucleotide binding loop, activation loop, and C-terminal tail. Structure. 2000 Nov 15;8(11):1105-13.

Therasse P, Arbuck SG, Eisenhauer EA, Wanders J, Kaplan RS, Rubinstein L et al. New Guidelines to Evaluate the Response to Treatment in Solid Tumors. JNCI Cancer Spectrum. 2000;92(3):205.

Xia L, Yuan YZ, Xu CD, Zhang YP, Qiao MM, Xu JX. Effects of epidermal growth factor on the growth of human gastric cancer cell and the implanted tumour of nude mice.World J Gastroenterol. 2002 Jun;8(3):455-8.

Yarden Y, Sliwkowski MX. Untangling the ErbB signalling network. Nat Rev Mol Cell Biol 2001; 2: 127-137

Zhou BP, Hung MC. Dysregulation of cellular signaling by HER2/neu in breast cancer. Semin Oncol. 2003 Oct;30(5 Suppl 16):38-48

15. APPENDICES

15.1. Appendix 1: ECOG Performance Status Scale

Grade	ECOG
0	Fully active, able to carry on all pre-disease performance without restriction
1	Restricted in physically strenuous activity, but ambulatory and able to carry out work of a light or sedentary nature, e.g., light house work, office work
2	Ambulatory and capable of all selfcare, but unable to carry out any work activities. Up and about more than 50% of waking hours
3	Capable of only limited selfcare, confined to bed or chair more than 50% of waking hours
4	Completely disabled. Cannot carry on any selfcare. Totally confined to bed or chair
5	Dead

Abbreviations: ECOG = Eastern Cooperative Oncology Group

15.2. Appendix 2: Cockcroft and Gault Method for Calculated Creatinine Clearance

Creatinine clearance can be calculated from serum creatinine values by one of the two formulas below:

$$\text{Calculated creatinine clearance (mL/min)} = \frac{(140 - \text{age [yrs]}) \times \text{weight (kg)}}{72 \times \text{serum creatinine (mg/DL)}}$$

Female patients**:** multiply by 0.85

In order to use SI units for creatinine (μmol/L), the following formula may be used.

$$\text{Calculated creatinine clearance (mL/min)} = \frac{(140 - \text{age [yrs]}) \times \text{weight (kg)} \times 1.23}{\text{serum creatinine } (\mu\text{mol/L}))}$$

Female patients**:** multiply by 0.85

15.3. Appendix 3: MATERIAL and METHOD

1. Preparation of blood sample and isolation and enumeration of CTCs in advanced breast cancer patients.

Immunicon is developing and commercializing diagnostic and research products for tumour cell analysis. Reagent kits incorporating Immunicon proprietary technology are used to capture, count and characterize circulating tumour cells or circulating endothelial cells from whole blood.

The CellTracks AutoPrep System is an automated sample preparation system for immunomagnetic cell selection. Reagents are added to each specimen and magnetic incubations are precisely timed. Many steps are automated, minimizing user interaction. Depending on the processing protocol, samples may be analyzed using the CellTracks Analyzer II (a semi-automated fluorescence microscope used to count and characterize the cells isolated by the CellTracks AutoPrep System.), flow cytometry or processed off-line for molecular analysis.

Analysis of Circulating Tumour Cells (CTCs)

The CellSearch Circulating Tumour Cell Kit is intended for the enumeration of circulating tumour cells (CTC) of epithelial origin (CD45-, EpCAM+, and cytokeratins 8, 18+, and/or 19+) in whole blood.

The presence of CTC in the peripheral blood, as detected by the CellSearch Circulating Tumour Cell Kit, is associated with decreased progression free survival and decreased overall survival in patients treated for metastatic breast cancer. A CTC count of 5 or more per 7.5 mL of blood is predictive of shorter progression free survival and overall survival in metastatic breast cancer patients (Cristofanilli et al).

Products and method for Analysis of Circulating Tumour Cells (CTCs):

- CellSave Preservative Tubes, intended for the collection and preservation of circulating epithelial cells (tumour cells) in whole blood, to be used for enumeration and phenotyping. Samples are stable for 72 hours after collection.
- CellTracks AutoPrep System
- CellTracks Analyzer II
- CellSearch EGFr Tumour Phenotyping Reagent: a fluorescein conjugated monoclonal antibody anti -EGFr designed to be processed on the CellTracks AutoPrep System with the CellSearch Circulating Tumour Cell Kit and analyzed with the CellTracks Analyzer II.

- CellSearch Circulating Tumour Cell Kit (Veridex). The kit contains reagents and supplies for 16 tests (14 patients samples + 2 control samples) including:
  - Anti-EpCAM Ferrofluid: to capture epithelial cells
  - Nucleic Acid Dye: DAPI to identify nucleated cells
  - Capture Enhancement Reagent: to maximize capture efficiency regardless of variable EpCAM expression
  - Permeabilization Reagent
  - Cell Fixative: to fix cells in the final samples
  - Staining Reagent: Anti-cytokeratin-PE and anti-CD45-APC to label epithelial cells and leukocytes respectively. A 4th color may be added to further characterize the cells (markers for HER2, EGFr or MUC-1 analysis)
  - CellTracks AutoPrep sample tubes, caps and cartridges

This technology enables essentially all of the cells from the starting samples (7.5 ml) to be analyzed with minimal loss.

10 ml of patient's blood is collected into CellSave Preservative Tubes (Immunicon) to ensure that cell morphology and antigen expression are preserved. Fill tube must be gently invert 8 times to prevent clotting and store at room temperature (15-30°C) until processing.

Samples will be stable for up to 72 hours and must be processed within 72 hours of collection.

Each sample must be examined for clotting before processing on CellTracks AutoPrep System. Clotted samples should be discarded because clots interfere with the performance of the assay.

7.5 ml blood from CellSave Preservative Tubes (Immunicon) is transfed into the conical AutoPrep tube (Immunicon) and 6.5 ml Dilution Buffer is added. Sample is mixed by inversion 5 times and then centrifuged at 800xg for 10 minutes with brake off.

The sample must be processed on the CellTracks Autoprep System within 1 hour of sample preparation.

The conical AutoPrep tube (Immunicon) and reagent kit (CellSearch Circulating Tumour Cell Kit) are loaded on CellTracks AutoPrep System (Immunicon). The CellTracks AutoPrep System automates and standardizes complex sample preparation operations.(Ferrofluid and staining reagents are added to each specimen, magnetic incubations are precisely timed and the final, enriched sample is dispensed into the MagNest cell presentation device).

Ferrofluids conjugated to antibodies directed against EpCam protein are used for isolation of CTCs from 7.5 ml whole blood. After the cells are enriched from the patient sample and fluorescently labelled with staining reagent and nucleic acid dye. The CellSearch HER2 Tumour Phenotyping Reagent is added to identify those CTCs over-expressing HER2.

Magnetic incubations are precisely timed and the final enriched sample is dispensed into the MagNest(Immunicon) cell presentation device that exerts a magnetic field that causes the magnetically labeled cells to form a monolayer on one focal plane inside the reaction cartridge. The filled cartridge within the MagNest device should be allowed to incubate in the dark for minimum of 20 minutes and analyzed within 24 hours.

The CellTracks Analyzer II (Immunicon) scans the reaction cartridge and displays a gallery of images of candidate targets to the user for classification. Captured images containing PE and DAPI positive events are presented in a gallery for analysis and classification of cells by the user, so cells CK+/DAPI+/CD45 are classified as CTCs and an event is classified as a tumour cell expressing HER-2 when its morphological features are consistent with that of a tumour cell and it exhibits the phenotype EpCAM +/CK+/DAPI+/CD45- and HER2 + .

The CellSearch Circulating Tumour Cell Control Kit (Veridex) is used to check reagents, instruments and operator technique. Each single use bottle contains two populations of a cultured cell line at different concentrations (Low and High). The two cell populations are differentially stained with fluorescent dyes that are specific for each population to permit simultaneous enumeration of Low and high Controls in one assay.

2. Isolation of Circulating Tumour Cells (CTCs) by CellSearch Profile Kit

The presence of epithelial cells in peripheral blood in normal healthy patients is very rare, but they have been found in low number in several malignant disease.

The CellSearch Profile Kit (Veridex) is designed to immunomagnetically enrich cells of epithelial origin in whole blood in conjunction with the Celltracks AutoPrep system. When CTCs have been enriched from whole blood, researchers may wish to investigate RNA expression or genomic DNA in these cells. Recently, the ability to perform multigene RT-PCR profiling of CTCs as been demonstrated. Molecular profiling of CTCs should lead to improved characterization of CTCs and ultimately to develop of novel therapeutic strategies.

The CellSearch Profile Kit (Veridex) contains a ferrofluid-based capture reagent coated with antibodies targeting the Epithelial Cell Adhesion molecule (EpCAM) antigen for capturing CTC. The CellTrack AutoPrep System precisely dispenses reagents and performs magnetic incubation steps. The majority of leukocytes and other blood components are depleted from the final sample, rendering it ready for further analysis using the laboratory’s validated methods and minimizing background.

Products and method for isolation of Circulating Tumour Cells (CTCs):

- CellSave Preservative Tubes (cellular analysis) or EDTA tubes (molecular analysis) for blood collection
- CellTracks AutoPrep System
- CellSearch Profile Kit The kit contains reagents and supplies for 16 tests including:
    - Anti-EpCAM Ferrofluid: to capture epithelial cells
    - Capture Enhancement Reagent: to maximize capture efficiency regardless of variable EpCAM expression
    - Dilution Buffer
    - CellTracks AutoPrep sample tubes and caps

10 ml (at least 7.5 ml) of patient's blood is collected aseptically by venipuncture or from a venous port.

For cellular analysis of the epithelial cells must be used the CellSave Preservative Tubes because CTCs are fragile and require preservation for accurate analysis. Fill tube must be gently invert 8 times to prevent clotting and store at room temperature (15-30°C) until processing.

Samples will be stable for up to 72 hours and must be processed within 72 hours of collection.

Each sample must be examined for clotting before processing on CellTracks AutoPrep System. Clotted samples should be discarded because clots interfere with the performance of the assay.

7.5 ml blood from CellSave Preservative Tubes (Immunicon) or EDTA tubes is transfed into the conical AutoPrep tube (Immunicon) and 6.5 ml Dilution Buffer is added. Sample is mixed by inversion 5 times and then centrifuged at 800xg for 10 minutes with brake off.

The sample must be processed on the CellTracks Autoprep System within 1 hour of sample preparation. The CellTracks AutoPrep System delivers a final volume of 900 mcl of CTC enriched sample. The final sample is ready for further analysis using the laboratory's validated methods.

3. Assessment of HER2 status by FISH method on primary breast cancer tissue and on CTCs

FISH on primary tumour or metastatic slides and on CTCs will be performed using the PathVysion HER-2 DNA FISH Kit, (Vysis Inc, Downers Grove, IL) according to the manufacturer's instructions.

The PathVysion HER-2 DNA Probe Kit (PathVysion Kit) is designed to detect amplification of the HER-2/neu gene via fluorescence *in situ* hybridization (FISH) in formalin-fixed, paraffin-embedded human breast cancer tissue specimens

The kit incorporates two directly labelled fluorescence DNA probes: an orange probe directed against the *HER2* gene (HER2, 17q11.2-q12) and a green probe directed against the pericentromeric region of chromosome 17, (CEP 17, 17p11.1-q11.1), the chromosome in which the *HER2* gene resides. To breakdown formaldehyde cross-links, acid pretreatment and protease digestion were performed (Vysis paraffin pre-treatment kit; Vysis Inc), followed by standard saline citrate (SSC) and formamide denaturation (72°C, 5 minutes). After dehydration, the HER2/CEP 17 probe cocktail was added, and coverslips were applied and sealed with rubber cement. Slides were incubated in a humidity chamber overnight for 18 hours at 37°C. On the following day, slides were washed in a stringency buffer (SSC, NP40), air-dried in the dark and incubated with 4,6 diamidino-2- phenylindole (DAPI) for nuclear identification. Slides were stored in the dark, at 20°C.

The pathologist counts HER-2 gene copy numbers in interphase nuclei of malignant cells. Since the "natural biological standard" for normal cells dictates that they contain two copies of HER-2 gene, interpretation of HER-2 status using PathVysion FISH can be measured precisely and with objectivity. The cut-off between normal and abnormal is set by biology; more than one copy of the HER-2 gene per chromosome 17 is abnormal. Determination of the ratio of HER-2 to chromosome 17 copy number is useful in the discrimination of aneusomy of chromosome 17 from true HER-2 gene amplification. This allows for the proper assessment of amplification levels, especially low level amplification

15.4. Appendix 4: PET Scan Sub-Study

All patients who satisfy the protocol and additional eligibility criteria will be invited to enrol for this sub-study looking at the early effects of lapatinib on tumour proliferation and MAP kinase activity. All PET scans will be performed at HAMMERSMITH IMANET at Hammersmith Hospital, Du Cane Road, London by one of the principal investigators. All patients will be consented separately for the PET procedure.

Title: Imaging modulation of MAPkinase activity using [^{11}C]choline and [^{18}F]FLT PET.

Background. Growth receptor activation of EGFR and HER-2 receptors is responsible for reactivation of ERά transcription through various signal transduction pathways following endocrine therapy. This leads to relapse of breast tumours associated with aggressive progression and poor outcomes [Nicholson, 2003]. Downstream signalling from these receptors is mainly by the MAPkinase cascade and the PI3kinase pathway. The MAPkinase cascade activation results in ERK1/2 phosphorylation which in turn causes ER phosphorylation at Ser-118 position leading to its ligand-independent phosphorylation [Gee, 2001].

Rationale: There is an unmet need to quantify early response of new investigational agents as well as finding biomarkers of response to newer therapies like lapatinib. The use of PET would lead to a rapid way of testing the effectiveness of new therapies. We plan to quantify the early effects of lapatinib reflected in modulating MAPkinase activity using the radioactive PET tracer [^{11}C]choline and proliferation of tumour using [^{18}F]Fluorothymidine(FLT). Previous work by our group has shown that inhibition of MAP kinase(ERK1/2) results in a dose dependent and time dependent inhibition of phosphocholine production in-vitro [Liu, 2002]. [^{18}F]Fluorothymidine(FLT) is a tracer which measures proliferation by imaging in-vivo Thymidine Kinase-1 activity which is the enzyme responsible for phosphorylation of thymidine before its incorporation into DNA. [^{18}F]FLT is phosphorylated by thymidine kinase-1(TK1). This leads to intracellular trapping of the tracer and prevents its further incorporation into DNA. Thus this tracer provides a readout of proliferative activity as shown previously in our group [Kenny, 2005].

Methods: This PET substudy is planned to recruit up to 14 patients (using the same sample size calculation as in our previous study with similar tracers) who have diagnosed metastatic breast cancer or metastatic disease which is progressing in spite of treatment. All patients would need to be ER positive. The other receptor status will be as per the main study. Patients should also have received some form of adjuvant endocrine treatment in the past for breast cancer. All patients will undergo a pre-treatment single session PET scan using [^{11}C]choline and [^{18}F]FLT. Three 10mls venous blood samples would be taken during each PET scan (totalling 30mls per PET scan) to calculate radioactivity counts and metabolite of the [11C] choline radiotracer**.** They will then be asked to start 1500mg/day of oral lapatinib. At the end of approximately two weeks of treatment, the same PET scan will be repeated to find out if early change in uptake of both tracers is demonstrable, reflecting early activity of lapatinib.

Statistical considerations: From reproducibility studies using [18F]FLT in untreated and metastatic breast cancer conducted by the same team the calculated standard deviation (SD) for Ki (constant for net irreversible uptake of [18F]FLT) was 2.0 and 3.61 in untreated and metastatic patients and the mean was 3.6 and 7.3 respectively. Preliminary results for [11C]choline are not available but are expected to be similar.

A sample size of approximately 14 in each group will have 80% power to detect a difference in means of 3.7 (the difference between the metastatic group mean, m1, of 7.3 and the primary group mean, m2, of 3.6) assuming that the metastatic group standard deviation, s1, is 3.61 and the primary group standard deviation, s2, is 2 (ratio of primary group to metastatic group standard deviation is 0.554) using a Wilcoxon (Mann-Whitney) rank-sum test with a 0.05 two-sided significance level. Statistics power calculation done by Dr Joseph Eliahoo, Imperial College Statistics Service.

Results: Dynamic images of PET will be analysed by drawing ROI (region of interest) around the tumours. A population arterial input function will be applied to the image data to derive various parameters of tracer delivery (K1) and irreversible uptake (Ki). Other parameters of uptake obtained will be SUV (standardised uptake value) and AUC (Area under tracer concentration-time curve). A significant change in uptake parameters from pre to post-treatment would imply inhibition of MAP kinase activity and proliferation due to the effect of lapatinib.

Primary End Point: Change in uptake of of [11C]choline and [18F]FLT from pre to post treatment with lapatinib.

The ability to image MAP kinase modulation using PET would serve as a marker of the pharmacological early effect of lapatinib.

Secondary End Point: None.

Radiation dosimetry:

For [11C]choline-effective dose=1.036mSv per scan [Kobori, 1999]

For[18F]FLT -effective dose=7.326mSv per scan [Vesselle, 2003]

Therefore the total effective dose per patient for two PET scans is 16.724mSv. The radiation dose is not significant as all patients will be exposed to radiation from Xrays, CT scans and bone scans which form part of routine investigations.

Inclusion criteria:

This will be as per the main study criteria. Additional criteria are:-

1. The tumour should be ER positive from any biopsy done before.
2. Metastatic region to be imaged should be at least 20mm in longest dimension using conventional means of measuring like X-ray, mammography, CT scan, callipers.

Exclusion criteria:

This will include all criteria as per the main study. Additional exclusions specific to the study are:-

1. Metastatic lesion in bone and/or liver only. This is because the physiological uptake of [11C]choline and [18F]FLT uptake is very high in these tissues, masking any uptake by tumour. However a patient having bone and/or liver metastasis along with metastatic deposit elsewhere is eligible eg: lungs, chest wall, axilla, breast, lymph nodes etc.
2. Pregnant, breast feeding and lactating ladies, due to possible radiation exposure to foetus/child.

Response criteria for PET study: PET will be used to quantify response by calculating the change in PET uptake parameters like SUV (Standardised Uptake Value), Ki (constant for net irreversible uptake of tracer) and FRT (Fractional retention time). This will help in identifying early responders to treatment. PET will help in measuring MAP kinase activity and proliferation, both of which have been shown to be down regulated by lapatinib via its upstream blockade of EGFR and HER-2 receptor tyrosine kinases.

References

Gee, J. M. Phosphorylation of ERK1/2 mitogen-activated protein kinase is associated with poor response to anti-hormonal therapy and decreased patient survival in clinical breast cancer. Int J Cancer 2001; 95: 247-254.

Kenny, L. M., D. M. Vigushin, et al. Quantification of cellular proliferation in tumor and normal tissues of patients with breast cancer by [18F]fluorothymidine-positron emission tomography imaging: evaluation of analytical methods. Cancer Res 2005; 65(21): 10104-12.

Kobori, O., Y. Kirihara, et al. Positron emission tomography of esophageal carcinoma using (11)C-choline and (18)F-fluorodeoxyglucose: a novel method of preoperative lymph node staging. Cancer; 1999; 86(9): 1638-48.

Liu, D., O. C. Hutchinson, et al. Use of radiolabelled choline as a pharmacodynamic marker for the signal transduction inhibitor geldanamycin. Br J Cancer 2002; 87(7): 783-9.

Nicholson, R. I., J. M. Gee, et al. The biology of antihormone failure in breast cancer. Breast Cancer Res Treat 2003; 80 Suppl 1: S29-34; discussion S35.

Vesselle, H., J. Grierson, et al. 18F-Fluorothymidine radiation dosimetry in human PET imaging studies. J Nucl Med 2003; 44(9): 1482-8.

15.5. Appendix 5: Liver Chemistry Stopping and Follow up Criteria

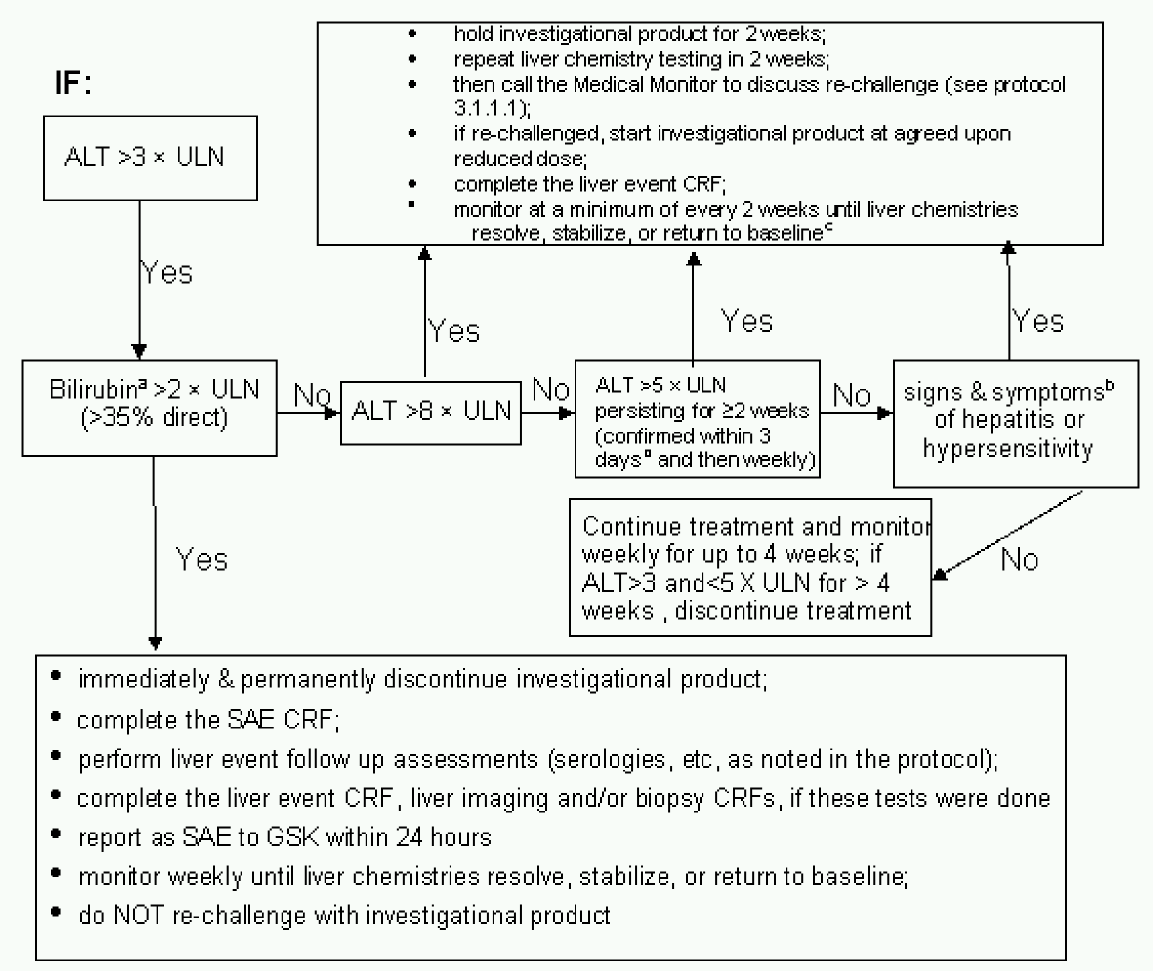

a. bilirubin fractionation should be performed if testing is available. If testing is unavailable and a subject meets the criterion of total bilirubin >2.0 × ULN, then the event should still be reported as an SAE and actions taken as described
b. the appearance or worsening of fatigue, nausea, vomiting, right upper quadrant pain or tenderness, fever, rash, or eosinophilia
c. once liver chemistries resolve, stabilize, or return to baseline, then continue monitoring per the protocol assessment schedule
d. retest within 3 days from the first occurrence and then weekly to determine if ALT elevation persists

15.6. Appendix 6: Changes to the protocol

Amendment 01 was filed in UK only and Amendment 02 was filed in Italy only. Details of these two amendments are included here.

This current amendment number 03 will amalgamate both amendments 01 and 02 with some further changes made. This amendment is a global amendment for both UK and Italy.

AMENDMENT 01

The original protocol dated 18 Apr 2007 has been changed to amend the following points. This Amendment will apply only in UK.

Reason for change

Section 8 of the protocol has been amended to include a section 8.6 following a request by the UK site to clarify the treatment related to the unlikely event of long term remission in, at least, one patient.

Original Text

No previous text

Amended text

Section 8.6 - Long term remission

In the study, there is not a defined stop for the study treatment. In the event of long term remission (i.e., patient has no progressive disease after 18 months of therapy), even unlikely, the following screening regimen is applied for these subjects:

Investigation	Timeframe	Number of procedures
PET scan	Screening then 2 weeks	2
Bone Scintogram	Screening then 3, 6, 12 and 18 months	5
MUGA scan	Screening	1
CAP CT	Screening then 3, 6, 9, 12 and 18 months	6
Bone X-Ray	Screening then 3, 6, 9, 12 and 18 months	6

In the event of long term remission, any imaging performed after 18 months will only be done if clinically indicated (i.e. under standard IR(ME)R practice it is only done if the results will be of benefit to the patient's clinical care).

Reason for change

Appendix 4 (Pet Scan sub-study) of the protocol has been amended in order to correct some errors put in the previous version of this section.

Original Text

Methods: […] Two 10ml venous blood samples would be taken during the scan (totalling 150ml of arterial blood) to calculate metabolite of the [11C]choline radiotracer. […]

Amended text

Methods: […] Three 10mls venous blood samples would be taken during each PET scan (totalling 30mls per PET scan) to calculate radioactivity counts and metabolite of the [11C] choline radiotracer. […]

AMENDMENT 02

The original protocol dated 18 Apr 2007 has been changed to amend the following points in accordance with the Italian Central Ethic Committee advice.

This Amendment will apply only in ITALY.

- page 26, section 6.3, from line 2 to line 5: delete the sentences: " From the ethical point of view this option is acceptable even as first-line treatment for metastatic disease because patients will receive other treatments after progression. In clinical practice breast cancer is always treated sequentially with various agents."

- page 28, section 6.3, after point n° 14: addition of point n° 15: "At least one line of treatment for metastatic disease"

In addition, taking advantage from this amendment the following safety changes are made:

- page 27, section 6.3, point n° 10, line 3: PLT≥ 100.000/mm³ (instead of ≥50.000/mm³)

- page 27, section 6.3, point n° 10, line 4: Hb≥ 10 g/dL (instead of ≥ 9 g/dL)

As well as the following typing errors are corrected:

- page 38, table 8, section "Safety assessment", line 7: deletion of "x" from column "Every 2 cycles" and from column "Every 12 weeks"

- page 38, table 8, section "Safety assessment", line 8: deletion of "x" from column "Up to 28 days prior 1st dose" and addition of "x" in column "Every cycles"

- page 38, table 8, section "Safety assessment", line 9: deletion of "x" from column "Up to 28 days prior 1st dose" and addition of "x" in column "Every cycles"

- page 74, section 3, title: …on primary breast cancer tissue and… (instead of: …on primary or metastatic breast cancer tissue and…)

AMENDMENT 03

Amendment 03 is a global amendment incorporating both previous amendments 01 and 02 with some further changes made as described below:

Sponsor Information page

Original text

SPONSOR INFORMATION PAGE

Clinical Study Identifier:	105594
Sponsor	GlaxoSmithKline<br>Greenford Road<br>Greenford, Middlesex, UB6 0HE, UK<br>Telephone: +44 (0)20 8 422 3434
Medical Monitor	Dr. Cristina Oliva<br>GlaxoSmithKline<br>Greenford Road<br>Greenford, Middlesex, UB6 0HE, UK<br>Telephone: +44 (0)20 8 422 3434<br>Mobile: +39 348 040 3200<br>E mail: cristina.r.oliva@gsk.com

Regulatory Agency Identifying Number(s): EudraCT Number 2007-002155-17

Amended Text

SPONSOR INFORMATION PAGE

Clinical Study Identifier:	105594
Sponsor	GlaxoSmithKline<br>Stockley Park West<br>Uxbridge<br>Middlesex<br>UB11 1BT<br>Tel. +44(0)20 8990 9000<br>Fax. +44(0)208990 4321

Medical Monitor Dr. Cristina Oliva
Stockley Park West
Uxbridge
Middlesex
UB11 1BT
Tel. +44 (0)20 8587 5453
Mobile +44 (0)7920567726
E mail: cristina.r.oliva@gsk.com

Regulatory Agency Identifying Number(s): EudraCT Number 2007-002155-17

Abbreviations

Following abbreviation has been added to the Abbreviations table

ER	Estrogen Receptor

Protocol Summary, section 5.1 Study Design and section 6.1 Strata

Original text

Stratum 2) UK study group: Advanced breast cancer patients with HER-2 non-amplified and EGFR amplified primary tumours and EGFR positive circulating tumour cells.

Amended Text

Stratum 2) UK study group: Advanced breast cancer patients with HER-2 non-amplified primary tumours and EGFR positive circulating tumour cells.

Background

2.2 Safety Profile of Lapatinib

New text added at end of section:

As part of ongoing pharmacovigilance by GlaxoSmithKline, a review of all hepatobiliary events reported across the entire lapatinib clinical development programme has been performed. Two hundred sixteen reports of hepatic events were retrieved from the GSK safety database as of 31 December 2007 regardless of source (clinical trials, spontaneous/marketed use data). In 39 of the 216 cases, a causal association to lapatinib could not be ruled out: 38.5% (15/39) of these subjects received lapatinib monotherapy, 53.8% (21/39) of subjects received lapatinib in combination with other chemotherapies, such as capecitabine, and 3 cases were still blinded.

A total of 13 deaths were identified which contained hepatobiliary events. In 3 of these cases, an association with lapatinib could not be excluded. The remaining 10 cases were confounded by the patients underlying condition (progressive disease and/or progression of pre-existing liver metastases).

Based on an additional sub-analysis, of 18 clinical studies of lapatinib in breast cancer, using Hy's Law (defined as AST or ALT >3 x ULN, and total bilirubin >2 x ULN, with no initial findings of cholestasis i.e.: ALP <2 x ULN) as a predictor for potential drug induced liver injury, the liver injury associated with lapatinib seems to be the result of a prolonged exposure to the drug. All the subjects whose events potentially met Hy's Law received study medication for three months or longer. The majority of these cases appeared reversible. Most patients experienced a decline in liver enzymes with drug cessation.

Based on the results of this review, GSK concluded a causal relationship between hepatobiliary disorders (specifically transaminase elevations) and lapatinib cannot be excluded. As a consequence, hepatotoxicity was added to the core safety information (CSI) for lapatinib. In addition, for ongoing clinical trials, the monitoring interval for hepatic function has been increased to every 4-6 weeks during treatment, and stopping rules have been added for severe hepatic events. Lapatinib dosing should be discontinued if changes in liver function are severe and patients should not be retreated.

2.2.1 Rationale for Starting Dose in Monotherapy

Original text

Gomez HL et al. conducted a phase II randomized study of lapatinib as first-line treatment for patient with HER-2 FISH-amplified metastatic breast cancer. One hundred and thirty patients were randomly assigned to receive lapatinib 1500 mg as a single daily dose (QD) or 500 mg twice daily (BID). An interim analysis was performed on the first 40 randomized patients. No unexpected toxicity was reported, and no grade 3/4 treatment-related adverse events were reported. Efficacy by dose schedule appeared comparable, and confirmed partial responses (PR) were demonstrated in 14 pts (35%) confirmed by RECIST criteria. Stable disease (SD) lasting at east 8 weeks was also demonstrated in 14 patients (35%). Median progression free survival was 16.4 weeks on 500mg BID and 20.0 weeks on 1500mg daily. The authors concluded that lapatinib appeared well tolerated and showed evidence of activity as first-line treatment for women with HER-2 amplified advanced breast cancer [Gomez, 2005].

Amended text

Gomez HL et al. conducted a phase II randomized study of lapatinib as first-line treatment for patient with HER-2 FISH-amplified metastatic breast cancer. One hundred and thirty-eight patients were randomly assigned to receive lapatinib 1500 mg as a single daily dose (QD) or 500 mg twice daily (BID). A total of 138 patients were treated with lapatinib for a median of 17.6 weeks. The overall response rate (complete response [CR] plus partial response [PR]) was 24% in the intent-to-treat population, and 31% of patients derived clinical benefit (CR, PR, or stable disease for _ 24 weeks). The median time to response was 7.9 weeks, and the progression-free survival rates at 4 and 6 months were 63% and 43%, respectively. The most common lapatinib-related adverse events (AEs) were diarrhea, rash, pruritus, and nausea, and these events were primarily grade 1 or 2. There were no significant differences in clinical activity or the AE profile between the dosing schedules. The authors concluded that lapatinib appeared well tolerated and

showed evidence of activity as first-line treatment for women with HER-2 amplified advanced breast cancer [Gomez, 2008].

3.3 Translational Research Objective

Original text

The translational research objective is to correlate response to lapatinib with HER-2 and EGFR protein levels and amplification evaluated on CTCs before starting treatment. A centralized review of the HER-2 status and EGFR status on the primary tumour will retrospectively be performed.

Amended text

The translational research objective is to correlate response to lapatinib with HER-2 and EGFR protein levels and amplification evaluated on CTCs before starting treatment. A centralized review of the HER-2 status on the primary tumour will retrospectively be performed.

Figure 1 Study Flow

Original text removed

*UK centre will only select patients with HER-2 negative EGFR amplified primary tumour.

6.3 Inclusion Criteria

Original text

Specific information regarding warnings, precautions, contraindications, adverse events, and other pertinent information on the investigational product that may impact subject eligibility is provided in lapatinib Investigator's Brochure Version 6.0 dated 30 March 2006 or product labels.

Amended text

Specific information regarding warnings, precautions, contraindications, adverse events, and other pertinent information on the investigational product that may impact subject eligibility is provided in the latest version of the lapatinib Investigator's Brochure or product labels.

Inclusion criteria 3

Original text

Histologically confirmed diagnosis of HER-2 negative (i.e. no gene amplification by FISH or IHC 2+ and no amplification by FISH or IHC 0/1+) infiltrating primary breast cancer. UK centres will additionally require patients with EGFR positive primary breast cancer (i.e., IHC 3+ or FISH)

Amended text

Histologically confirmed diagnosis of HER-2 negative (i.e. no gene amplification by FISH or IHC 2+ and no amplification by FISH or IHC 0/1+) infiltrating primary breast cancer.

Inclusion criteria 4

Original text

Evidence of HER-2 or EGFR positive circulating tumour cells (≥50% CTCs or FISH positive) in a peripheral blood sample taken at screening visit.

Amended text

Evidence of EGFR positive circulating tumour cells or HER-2 positive (≥50% CTCs or FISH positive) in a peripheral blood sample taken at screening visit.

Inclusion criteria 8

Original text

Eastern Cooperative Oncology Group (ECOG) score for performance status 0 to 2 (Appendix 1)

Amended text

Eastern Cooperative Oncology Group (ECOG) score for performance status 0 to 3 (Appendix 1)

Inclusion Criteria 10

Original text

Renal function:

- serum creatinine ≤ 2.0mg/dL or Calculated Creatinine (Appendix 2)
- Clearance ≥ 25mL/min

Amended Text

Renal function:

- serum creatinine ≤ 2.0mg/dL or Calculated Creatinine Clearance ≥ 25mL/min (Appendix 2)

Renal function:

serum creatinine = 2.0mg/dL or Calculated Creatinine Clearance ≥ 25mL/min

6.4 Exclusion criteria

Exclusion criteria #1

Original text

Any unstable systemic disease including active infections, significant cardiovascular disease, as well as myocardial infarction within the previous year, any significant hepatic, renal or metabolic disease, metabolic dysfunction, physical examination finding, or clinical laboratory finding that contraindicates the use of study medication or that might affect the interpretation of the results or render the patient at high risk from treatment complications.

Amended text

Any unstable systemic disease including active infections, significant cardiovascular disease, as well as myocardial infarction within the previous year, any significant hepatic (current active hepatic or biliary disease [with exception of patients with Gilbert's syndrome, asymptomatic gallstones, liver metastases or stable chronic liver disease per investigator assessment]), renal or metabolic disease, metabolic dysfunction, physical examination finding, or clinical laboratory finding that contraindicates the use of study medication or that might affect the interpretation of the results or render the patient at high risk from treatment complications.

Exclusion criteria #8

Original text

Concurrent radiotherapy to the only target lesion or concurrent bisphosphonates if bone metastases are the only target lesions

Amended text

Concurrent radiotherapy to the only target lesion. Concurrent bisphosphonates are allowed as long as this therapy has started before patient receives study medication.

Section 7.3 Dose Adjustment, Delays and Dose Modifications, Table 1 and 2

Original text

Table 1 Lapatinib Starting Dose and Dose Reduction Schedule

Starting dose	1500 mg/day	
First dose reduction	1250 mg/day	If no recovery after 3 weeks of holding drug, patients must be withdrawn from the study unless in the opinion of the investigator & GSK Medical Monitor, there is reason to believe that the patient is still experiencing clinical benefit

Patients requiring a second dose reduction should be taken off study unless in the opinion of the investigator & GSK Medical Monitor, there is reason to believe that the patient is still experiencing clinical benefit

Table 2 Dose Reduction Criteria and Guidelines for Management of lapatinib Associated Toxicity

Toxicity	Grade	Guideline for Management	Lapatinib Dose Modification[1]
Diarrhea	1	stop all lactose containing products, drink 8-10 large glasses of clear liquids a day, eat frequent small meals	None
	2	Loperamide (4 mg at first onset, followed by 2 mg every 2–4 hrs until diarrhea free for 12 hrs)	Consider a dose reduction of lapatinib (discuss with medical monitor)
	≥ 3(*despite optimal use of loperamide)*		Hold until recovery to ≤ grade 1, up to 21 days[1]. And then reduce 1 dose level.
Rash	1	No intervention	None
	2	Any of the following: minocycline[3], topical tetracycline or clindamycin, topical silver sulfadiazine, diphenhydramine, oral prednisone (short course)	None; If unacceptable to patient or medically concerning then hold until recovery to ≤ grade 1, up to 21 days[1]. Restart at same dose[2].
	≥ 3		Hold until recovery to ≤ grade 1, up to 21 days[1]. And then reduce 1 dose level.
Other Toxicity (excluding left ventricular dysfunction and pneumonitis, see below)	1	No Intervention	None
	2	Treatment as appropriate	None; If unacceptable to patient or medically concerning then hold until recovery to ≤ grade 1, up to 21 days[1]. Restart at same dose[2].
	2 prolonged or clinically significant and grade ≥ 3	Treatment as appropriate	Hold until recovery to < grade 1, up to 21 days and then reduce 1 dose level[1]

1. if no recovery after 3 weeks of holding drug, patients must be withdrawn from unless in the opinion of the investigator & GSK Medical Monitor, there is reason to believe that the patient is still experiencing clinical benefit.
2. if dose has been previously held for grade 2 toxicity and grade 2 symptoms recur, OR if the patient finds the symptoms unacceptable, hold dose until recovery to < grade 1 and then reduce dose one level
3. recommended dose: 200mg po bid (loading dose), followed by 100mg po bid for 7-10 days

Amended Text

Table 1 Lapatinib Starting Dose and Dose Reduction Schedule

Starting dose	1500 mg/day	
First dose reduction	1250 mg/day	If no recovery after 2 weeks of holding drug, patients must be withdrawn from the study unless in the opinion of the investigator & GSK Medical Monitor, there is reason to believe that the patient is still experiencing clinical benefit

Patients requiring a second dose reduction should be taken off study unless in the opinion of the investigator & GSK Medical Monitor, there is reason to believe that the patient is still experiencing clinical benefit

Table 2 Dose Reduction Criteria and Guidelines for Management of lapatinib Associated Toxicity

Toxicity	Grade	Guideline for Management	Lapatinib Dose Modification[1]
Diarrhea	1	stop all lactose containing products, drink 8-10 large glasses of clear liquids a day, eat frequent small meals	None
	2	Loperamide (4 mg at first onset, followed by 2 mg every 2–4 hrs until diarrhea free for 12 hrs)	Consider a dose reduction of lapatinib (discuss with medical monitor)
	≥ 3(*despite optimal use of loperamide)*		Hold until recovery to ≤ grade 1, up to 21 days[1]. And then reduce 1 dose level.
Rash	1	No intervention	None
	2	Any of the following: minocycline[3], topical tetracycline or clindamycin, topical silver sulfadiazine, diphenhydramine, oral prednisone (short course)	None; If unacceptable to patient or medically concerning then hold until recovery to ≤ grade 1, up to 21 days[1]. Restart at same dose[2].
	≥ 3		Hold until recovery to ≤ grade 1, up to 21 days[1]. And then reduce 1 dose level.
Other Toxicity (excluding left ventricular dysfunction and pneumonitis, see below)	1	No Intervention	None
	2	Treatment as appropriate	None; If unacceptable to patient or medically concerning then hold until recovery to ≤ grade 1, up to 21 days[1]. Restart at same dose[2].
	2 prolonged or clinically significant and grade ≥ 3	Treatment as appropriate	Hold until recovery to < grade 1, up to 21 days and then reduce 1 dose level[1]

1. if no recovery after 2 weeks of holding drug, patients must be withdrawn from unless in the opinion of the investigator & GSK Medical Monitor, there is reason to believe that the patient is still experiencing clinical benefit.
2. if dose has been previously held for grade 2 toxicity and grade 2 symptoms recur, OR if the patient finds the symptoms unacceptable, hold dose until recovery to < grade 1 and then reduce dose one level
3. recommended dose: 200mg po bid (loading dose), followed by 100mg po bid for 7-10 days

Section 7.3 Dose Adjustment, Delays and Dose Modifications

Original text

Non-Hematologic Toxicity	Any Grade 3 or 4 drug-related toxicity
Hematology	ANC is < 1.0 x 109/L<br>Platelet count is < 75.0 x 109/L
Chemistry	Unresolved grade 3 or 4 toxicity (except bilirubin)<br>Bilirubin is >5 N if documented liver metastases or >3 N without metastases
Serum Creatinine and Calculated Creatinine Clearance[1]	≥2.0 mg/dL<br>≤ 25 mL/min

1. Calculated by the Cockcroft and Gault Method

Amended text

Non-Hematologic Toxicity	Any Grade 3 or 4 drug-related toxicity
Hematology	ANC is < 1.0 x 109/L<br>Platelet count is < 75.0 x 109/L
Chemistry	Unresolved grade 3 or 4 toxicity (except bilirubin)<br>See Section 7.3.2 for liver function test abnormalities
Serum Creatinine and Calculated Creatinine Clearance[1]	≥2.0 mg/dL<br>≤ 25 mL/min

1. Calculated by the Cockcroft and Gault Method

Section 7.3.1 Criteria for Evaluating Asymptomatic Cardiac events Table 3 and Table 6

Original Text

If a subject who is receiving full dose of Lapatinib experiences a ≥20% decrease in LVEF relative to baseline AND the LVEF is below the institution's lower limit of normal (LLN), another evaluation of LVEF must be performed 2 weeks later while still receiving study drug.

Upon completion of the first repeat evaluation of LVEF, the procedures described in Table 3, Table 4, Table 5, and Table 6 must be followed:

Table 3 Criteria for Continuing Study Drug Following First Repeat Cardiac Evaluation Performed 2 Weeks Later While Still Receiving Study Drug

Institution's Range	LVEF Relative Change From Baseline	Action to be Taken
below LLN	≥20%	temporarily withdraw study drug<br>repeat cardiac evaluation in 2 wks and follow procedures in Table 4
below LLN	<20%	reduce to 4 tablets (equivalent to 1000mg QD active drug)<br>repeat cardiac evaluation in 2 wks & follow procedures in Table 5
WNL	≥20%	continue study drug<br>repeat cardiac evaluation in 2 wks and follow procedures in Table 6
WNL	<20%	continue study drug<br>continue cardiac evaluation every 2 months; refer to Time & Events Table 8.

Abbreviations: LLN = lower limit of normal; LVEF = left ventricular ejection fraction; WNL = within normal limits

Table 4 Criteria for Continuing Study Drug Following Second Repeat Cardiac Evaluation in Patients with LVEF below LLN and ≥20% Relative Change from Baseline

Institution's Range	LVEF Relative Change From Baseline	Action to be Taken
below LLN	≥20%	permanently withdraw study drug<br>repeat cardiac evaluation every 4 wks for at least 16 wks or until resolution
below LLN	<20%	permanently withdraw study drug<br>repeat cardiac evaluation every 4 wks for at least 16 wks or until resolution
WNL	≥20%	permanently withdraw study drug<br>repeat cardiac evaluation every 4 wks for at least 16 wks or until resolution
WNL	<20%	reduce to 4 tablets (equivalent to 1000mg QD active drug)<br>continue cardiac evaluation every 3 months; refer to Time & Events Table 8.

Abbreviations: LLN = lower limit of normal; LVEF = left ventricular ejection fraction; WNL = within normal limits

Table 5 Criteria for Continuing Study Drug Following Second Repeat Cardiac Evaluation in Patients with LVEF below LLN and <20% Relative Change from Baseline

Institution's Range	LVEF Relative Change From Baseline	Action to be Taken
below LLN	≥20%	permanently withdraw study drug<br>repeat cardiac evaluation every 4 wks for at least 16 wks or until resolution
below LLN	<20%	permanently withdraw study drug<br>repeat cardiac evaluation every 4 wks for at least 16 wks or until resolution
WNL	≥20%	permanently withdraw study drug<br>repeat cardiac evaluation every 4 wks for at least 16 wks or until resolution
WNL	<20%	continue at reduced dose (1000mg OD)<br>continue cardiac evaluation every 2 months; refer to Time & Events Table 8

Abbreviations: LLN = lower limit of normal; LVEF = left ventricular ejection fraction; WNL = within normal limits

Table 6 Criteria for Continuing Study Drug Following Second Repeat Cardiac Evaluation in Patients with LVEF WNL and ≥20% Relative Change from Baseline

Institution's Range	LVEF Relative Change From Baseline	Action to be Taken
below LLN	≥20%	permanently withdraw study drug<br>repeat cardiac evaluation every 4 wks for at least 16 wks or until resolution
below LLN	<20%	permanently withdraw study drug<br>repeat cardiac evaluation every 4 wks for at least 16 wks or until resolution
WNL	≥20%	Reduce to 4 tablets (equivalent to 1000mg OD active drug)<br>continue cardiac evaluation every 2 months; refer to Time & Events Table 8.
WNL	<20%	continue study drug<br>continue cardiac evaluation every 3 months; refer to Time & Events Table 8.

Abbreviations: LLN = lower limit of normal; LVEF = left ventricular ejection fraction; WNL = within normal limits

Amended text

7.3.1 Criteria for Evaluating Asymptomatic Cardiac events

If a subject who is receiving full dose of Lapatinib experiences a ≥20% decrease in LVEF relative to baseline AND the LVEF is below the institution's lower limit of normal (LLN), another evaluation of LVEF must be performed 2 weeks later while still receiving study drug.

Upon completion of the first repeat evaluation of LVEF, the procedures described in Table 3, Table 4, Table 5, and Table 6 must be followed:

Table 3 Criteria for Continuing Study Drug Following First Repeat Cardiac Evaluation Performed 2 Weeks Later While Still Receiving Study Drug

Institution's Range	LVEF Relative Change From Baseline	Action to be Taken
below LLN	≥20%	temporarily withdraw study drug<br>repeat cardiac evaluation in 2 wks and follow procedures in Table 4
below LLN	<20%	reduce to 5 tablets (equivalent to 1250mg QD active drug)<br>repeat cardiac evaluation in 2 wks & follow procedures in Table 5
WNL	≥20%	continue study drug<br>repeat cardiac evaluation in 2 wks and follow procedures in Table 6
WNL	<20%	continue study drug<br>continue cardiac evaluation every 2 months; refer to Time & Events Table 8.

Abbreviations: LLN = lower limit of normal; LVEF = left ventricular ejection fraction; WNL = within normal limits

Table 4 Criteria for Continuing Study Drug Following Second Repeat Cardiac Evaluation in Patients with LVEF below LLN and ≥20% Relative Change from Baseline

Institution's Range	LVEF Relative Change From Baseline	Action to be Taken
below LLN	≥20%	permanently withdraw study drug<br>repeat cardiac evaluation every 4 wks for at least 16 wks or until resolution
below LLN	<20%	permanently withdraw study drug<br>repeat cardiac evaluation every 4 wks for at least 16 wks or until resolution
WNL	≥20%	permanently withdraw study drug<br>repeat cardiac evaluation every 4 wks for at least 16 wks or until resolution
WNL	<20%	reduce to 5 tablets (equivalent to 1250mg QD active drug)<br>continue cardiac evaluation every 2 months; refer to Time & Events Table 8.

Abbreviations: LLN = lower limit of normal; LVEF = left ventricular ejection fraction; WNL = within normal limits

Table 5 Criteria for Continuing Study Drug Following Second Repeat Cardiac Evaluation in Patients with LVEF below LLN and <20% Relative Change from Baseline

Institution's Range	LVEF Relative Change From Baseline	Action to be Taken
below LLN	≥20%	permanently withdraw study drug<br>repeat cardiac evaluation every 4 wks for at least 16 wks or until resolution
below LLN	<20%	permanently withdraw study drug<br>repeat cardiac evaluation every 4 wks for at least 16 wks or until resolution
WNL	≥20%	permanently withdraw study drug<br>repeat cardiac evaluation every 4 wks for at least 16 wks or until resolution
WNL	<20%	continue at reduced dose (1250mg OD)<br>continue cardiac evaluation every 2 months; refer to Time & Events Table 8

Abbreviations: LLN = lower limit of normal; LVEF = left ventricular ejection fraction; WNL = within normal limits

Table 6 Criteria for Continuing Study Drug Following Second Repeat Cardiac Evaluation in Patients with LVEF WNL and ≥20% Relative Change from Baseline

Institution's Range	LVEF Relative Change From Baseline	Action to be Taken
below LLN	≥20%	permanently withdraw study drug<br>repeat cardiac evaluation every 4 wks for at least 16 wks or until resolution
below LLN	<20%	permanently withdraw study drug<br>repeat cardiac evaluation every 4 wks for at least 16 wks or until resolution
WNL	≥20%	Reduce to 5 tablets (equivalent to 1250mg OD active drug)<br>continue cardiac evaluation every 2 months; refer to Time & Events Table 8.
WNL	<20%	continue study drug<br>continue cardiac evaluation every 2 months; refer to Time & Events Table 8.

Abbreviations: LLN = lower limit of normal; LVEF = left ventricular ejection fraction; WNL = within normal limits

Section 7.3.2 added:

Liver Chemistry Stopping and Follow Up Criteria

Liver Chemistry Stopping Criteria

Liver chemistry stopping and follow up criteria have been designed to assure subject safety and evaluate liver event etiology. All subjects who meet liver chemistry criteria requiring permanent discontinuation of investigational product must continue to be followed for the study assessments and procedures as defined in Section 8.0 Study Assessments and Procedures and at the time points indicated in the Time & Events Table 8 in Section 8.0 Study Assessments and Procedures .

If a subject experiences ALT >3 × ULN and total bilirubin >2.0 × ULN (>35% direct; bilirubin fractionation required*), then the following actions must be taken:

- immediately and permanently discontinue investigational product;
- complete the SAE data collection tool, the liver event CRF, and the liver imaging and/or liver biopsy CRFs, if these tests are performed;
- in addition to the liver event follow up assessments defined in Section 7.3.2.2 Liver Chemistry Follow-up Criteria below, the following are suggested: specialist or hepatology consultation; anti-nuclear antibody, anti-smooth muscle antibody, and Type 1 anti-liver kidney microsomal antibodies; and liver imaging and/or liver biopsy to evaluate liver disease;

- promptly report the event to GSK within 24 hours of learning its occurrence (refer to Section 9.7.3 Prompt Reporting of Serious Adverse Events and other evens to GSK for guidance on prompt reporting to GSK);
- monitor every week until liver chemistries resolve, stabilize or return to within baseline values;
- do not re-challenge with investigational product.

***NOTE**: bilirubin fractionation should be performed if testing is available. If testing is unavailable and a subject meets the criterion of total bilirubin >2.0 × ULN, then the aforementioned actions must still be performed.

If a subject experiences:

- ALT >8 × ULN or
- ALT >5 × ULN persisting for ≥2 weeks : retest within 3 days from the first occurrence and then weekly to determine if ALT elevation persists or
- ALT >3 × ULN with signs or symptoms of hepatitis or hypersensitivity (the appearance or worsening of fatigue, nausea, vomiting, right upper quadrant pain or tenderness, fever, rash, or eosinophilia),

then hold investigational product for 2 weeks, repeat liver chemistry testing in 2 weeks, and then call the Medical Monitor to discuss the possibility of re-challenging with investigational product.

If the treatment is exhibiting efficacy **and** the subject wants to continue for potential benefit of lapatinib therapy after being informed of the results of liver chemistry testing, then the investigational product may be re-started at the reduced dose agreed upon by the investigator and the Medical Monitor. The liver event CRF should be completed and liver chemistries and aforementioned signs and symptoms should be monitored at a minimum of every 2 weeks until resolution, stabilization, or a return to baseline values, at which point monitoring should be continued per protocol.

If a subject experiences ALT >3 × ULN **but** <5 × ULN **and** total bilirubin ≤2 × ULN, without signs or symptoms of hepatitis or hypersensitivity, **and** who can be monitored weekly, then the following actions should be taken:

- continue investigational product;
- monitor weekly until liver chemistries resolve, stabilize, or return to within baseline, then monitor liver chemistries as per protocol assessment schedule;
    - if ALT >3 and < 5 × ULN for > 4 weeks , discontinue the treatment;
- if at any time this subject meets any of the aforementioned liver chemistry stopping criteria, then proceed as described above.

Liver Chemistry Follow up Criteria

For all subjects who meet any of the liver chemistry criteria described above, make every attempt to carry out the liver event follow up assessments described below:

- Viral hepatitis serology including:
  - Hepatitis A IgM antibody;
  - Hepatitis B surface antigen and Hepatitis B Core Antibody (IgM);
  - Hepatitis C RNA;
  - Cytomegalovirus IgM antibody;
  - Epstein-Barr viral capsid antigen IgM antibody (or if unavailable, obtain heterophile antibody or monospot testing);
  - Hepatitis E IgM antibody (if subject resides or has travelled outside USA or Canada in past 3 months);
- Serum creatine phosphokinase (CPK) and lactate dehydrogenase (LDH);
- Complete blood count with differential to assess eosinophilia;
- Record the appearance or worsening of clinical symptoms of hepatitis, or hypersensitivity, fatigue, decreased appetite, nausea, vomiting, abdominal pain, jaundice, fever, or rash as relevant on the AE report form;
- Record use of concomitant medications, acetaminophen, herbal remedies, other over the counter medications, or putative hepatotoxins, on the concomitant medications report form;
- Record alcohol use on the liver event alcohol intake case report form.

Refer to Section 15.5, Appendix 5 for a liver safety algorithm detailing stopping and follow up criteria.

A blood sample for PK analysis (approx. 3ml- see Study Manual) should be obtained for measurement of lapatinib concentration as soon as possible after the event is reported. The date, time, and amount of the last dose ingested by the subject, and the date and time at which the blood sample is obtained, should be recorded. Please see the Study Manual for PK sample instructions and collection details.

Original text

Two blood samples (10 +10 ml) will be shipped to the respective central laboratory for CTC evaluation. If the analysis is positive for presence of HER-2 over-expressing CTCs, *HER2* gene amplification will be evaluated by FISH. The patient will be eligible if the following criteria are met:

- For HER-2 positive CTCs
  - ≥ 50% CTCs are HER-2 positive and FISH in CTCs is positive or inconclusive

- < 50% CTCs are HER-2 positive and FISH in CTCs is positive
- For EGFR positive CTCs
  - ≥ 50% CTCs are EGFR positive and FISH in CTCs is positive or inconclusive
  - < 50% CTCs are EGFR positive and FISH in CTCs is positive

Amended text

Two blood samples (10 +10 ml) will be shipped to the respective central laboratory for CTC evaluation. If the analysis is positive for presence of HER-2 over-expressing CTCs, *HER2* gene amplification will be evaluated by FISH. The patient will be eligible if the following criteria are met:

- For HER-2 positive CTCs
  - ≥ 50% CTCs are HER-2 positive and FISH in CTCs is positive or inconclusive
  - < 50% CTCs are HER-2 positive and FISH in CTCs is positive
- For EGFR positive CTCs
  - Presence of EGFR positive CTCs

Table 8 Time and Events Table

Original Text

4 To be performed only if bone metastasses have been diagnosed

Amended text

4 To be performed only if bone metastases are clinically indicated.

Section 8.3.1. Efficacy-Methods, Scope and Schedules

Original text

Total tumour burden must be assessed within 1 week before starting first dose of lapatinib treatment

Amended Text

Total tumour burden must be assessed within 4 weeks before starting first dose of lapatinib treatment

Section 8.4.4. Clinical Laboratory Assessment

New text added:

Bilirubin fractionation is recommended if total bilirubin >2 x ULN when testing is available.

See Section 7.3.2 for details on Liver Chemistry Stopping and Follow-up criteria.

Section 9.2 Definition of a SAE

New text added:

- Hepatobiliary events have been seen in subjects taking lapatinib and other tyrosine kinase inhibitors. As a precaution, the following will be reported as an SAE:
    - ALT >3 × ULN and total bilirubin >2.0 × ULN (>35% direct; bilirubin fractionation required).

  NOTE: bilirubin fractionation should be performed if testing is available. If testing is unavailable and a subject meets the criterion of total bilirubin >2.0 × ULN, then the event should still be reported as an SAE.

Other hepatic events should be documented as an AE or an SAE as appropriate.

Section 9.7.3 Prompt recording of serious adverse Events and Other Events to GSK

Original text:

SAEs and pregnancies will be reported promptly to GSK as described in the following table once the investigator determines that the event meets the protocol definition for that event.

	Initial Reports		Follow-up Information on a Previous Report	
Type of Event	Time Frame	Documents	Time Frame	Documents
All SAEs	24 hours	“SAE” data collection tool	24 hours	Updated “SAE” data collection tool

Amended text:

SAEs, pregnancies, and liver function abnormalities meeting pre-defined stopping criteria will be reported promptly to GSK as described in the following table once the investigator determines that the event meets the protocol definition for that event.

	Initial Reports		Follow-up Information on a Previous Report	
Type of Event	Time Frame	Documents	Time Frame	Documents
All SAEs	24 hours	“SAE” data collection tool	24 hours	Updated “SAE” data collection tool
Pregnancy	2 Weeks	Pregnancy Notification Form	2 Weeks	Pregnancy Follow up Form
Liver chemistry abnormalities:				
ALT >3 × ULN and bilirubin[a] >2 × ULN (35% direct)	24 hours	Liver Event and Liver Imaging and/or Biopsy CRFs, if applicable	24 hours	Updated Liver Event CRF

a. bilirubin fractionation should be performed if testing is available. If testing is unavailable and a subject meets the criterion of total bilirubin >2.0 × ULN, then the event should still be promptly reported as defined

Next figure added:

Section 15.5 Appendix 5: Liver Chemistry Stopping and Follow up Criteria

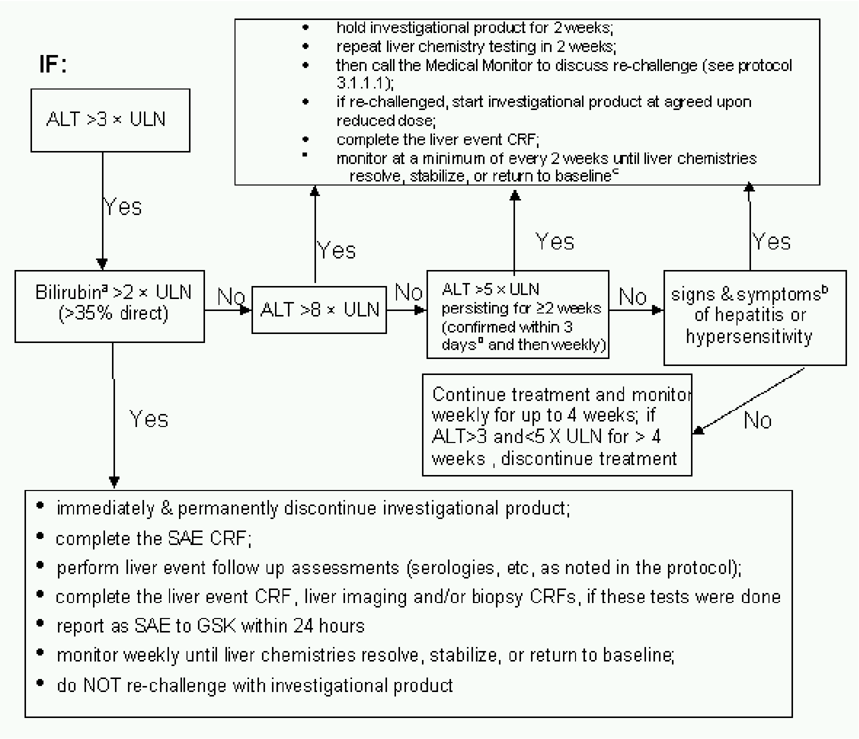

a. bilirubin fractionation should be performed if testing is available. If testing is unavailable and a subject meets the criterion of total bilirubin >2.0 × ULN, then the event should still be reported as an SAE and actions taken as described
b. the appearance or worsening of fatigue, nausea, vomiting, right upper quadrant pain or tenderness, fever, rash, or eosinophilia
c. once liver chemistries resolve, stabilize, or return to baseline, then continue monitoring per the protocol assessment schedule
d. retest within 3 days from the first occurrence and then weekly to determine if ALT elevation persists

Appendix 4- names of investigators removed. Names of current investigators will be kept in Study Files.

Original Text

Chief Investigators:	Prof R.C Coombes
Principal Investigators:	Prof Eric Aboagye<br>Mr K.B Contractor<br>Dr J Stebbing

Amended text

N/A
